# Supplementary material for: Avian Pattern Recognition Receptor Sensing and Signaling
Source: Vet Sci. 2020 Jan 27;7(1):14. doi: 10.3390/vetsci7010014 (PMC7157566; doi:10.3390/vetsci7010014)
Supplement: Supplementary file 1 [file vetsci-07-00014-s001.pdf]

Table S1: Accession numbers of PRR protein sequences used for multiple sequence alignments and reviewed in the present manuscript.

PRR protein	Chicken	Duck	Goose
TLR2t1	NP_989609.1	XP_005021002.2	XP_013030624.1
TLR2t2	NP_001155122.1	NP_001297277.1	-
TLR1t1	NP_001007489.4	NP_001297353.1	XP_013041417.1
TLR1t2	XP_025005535.1	XP_027312049.1	-
TLR3	NP_001011691.3	NP_001297711.1	XP_013035229.1
TLR4	NP_001025864.1	NP_001297342.1	XP_013038385.1
TLR5	NP_001019757.1	NP_001297753.1	XP_013029406.1
TLR7	NP_001011688.1	XP_005029235.1	XP_013046996.1
TLR21	NP_001025729.1	KY829021	AMB20882.1
TLR15	NP_001032924.1	XP_005018927.2	XP_013046493.1
RIG-I	-	NP_001297309.1	NP_001298119.1
MDA-5	NP_001180567.1	NP_001297740.1	XP_013026596.1
LGP2	NP_001305337.1	XP_027301625.1	XP_013055203.1
DDX1	NP_989894.1	XP_027311055.1	XP_013028107
DDX21	XP_001232052	XP_027316557.1	XP_013030257.1
DHX36	XP_015147310.1	XP_027320349.1	XP_013048509.1
DDX3X	NP_001025971.1	XP_005012555.2	XP_013046370.1
DDX23	XP_025001441.1	XP_021135293.1	XP_013054789.1
DDX24	NP_065147.1	XP_025006699.1	XP_027314331.1
DDX60	XP_004940975.1	XP_027312235.1	XP_013030722.1
DDX41	NP_001336637.1	XP_027324165.1	XP_013047316.1
DHX36	XP_015147310.1	XP_027320349.1	XP_013048508.1
OASL	XP_015148492.1	ARS01326.1	XP_013047372.1
cGAS	XP_419881.4	XP_027310983.1	XP_013027663.1
STING	XP_025010645.1	XP_027323921.1	XP_013057484.1
ERADp	NP_001185786.1	XP_027323010.1	XP_013042385.1

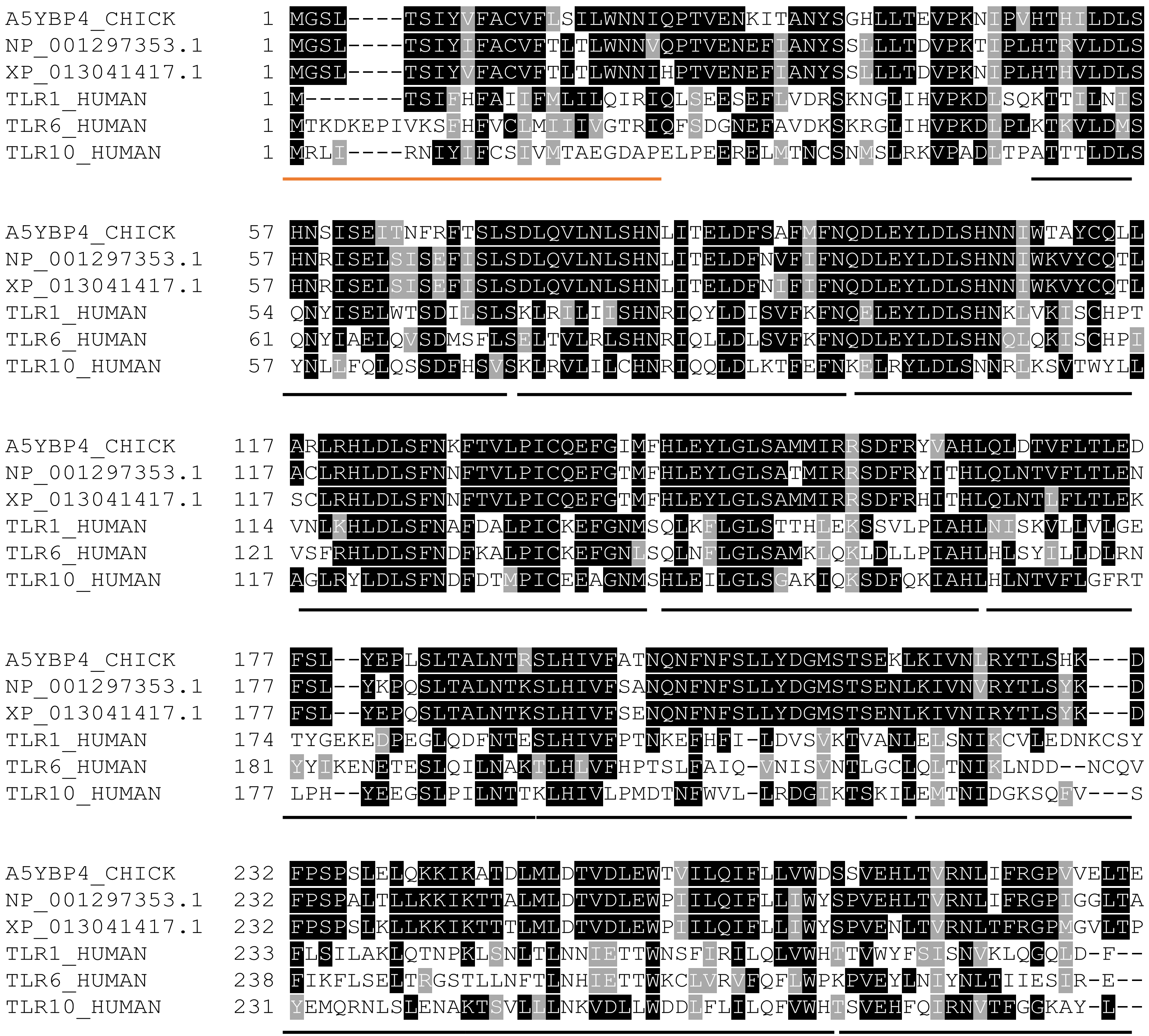
A5YBP4_CHICK 1 MGSL----TSIYVFACVFLSILWNNIQPTVENKITANYSGHLLTEVPKNIPVHTHILDLS
NP_001297353.1 1 MGSL----TSIYIFACVFTLTLWNNVQPTVENEFIANYSSLLLTDVPKTIPLHTRVLDLS
XP_013041417.1 1 MGSL----TSIYVFACVFTLTLWNNIHPTVENEFIANYSSLLLTDVPKNIPLHTHVLDLS
TLR1_HUMAN 1 M-------TSIFHFAIIFMLILQIRIQLSEESEFLVDRSKNGLIHVPKDLSQKTTILNIS
TLR6_HUMAN 1 MTKDKEPIVKSFHFVCLMIIIVGTRIQFSDGNEFAVDKSKRGLIHVPKDLPLKTKVLDMS
TLR10_HUMAN 1 MRLI----RNIYIFCSIVMTAEGDAPELPEERELMTNCSNMSLRKVPADLTPATTTLDLS
A5YBP4_CHICK 57 HNSISEITNFRFTSLSDLQVLNLSHNLITELDFSAFMFNQDLEYLDLSHNNIWTAYCQLL
NP_001297353.1 57 HNRISELSISEFISLSDLQVLNLSHNLITELDFNVFIFNQDLEYLDLSHNNIWKVYCQTL
XP_013041417.1 57 HNRISELSISEFISLSDLQVLNLSHNLITELDFNIFIFNQDLEYLDLSHNNIWKVYCQTL
TLR1_HUMAN 54 QNYISELWTSDILSLSKLRILIISHNRIQYLDISVFKFNQELEYLDLSHNKLVKISCHPT
TLR6_HUMAN 61 QNYIAELQVSDMSFLSELTVLRLSHNRIQLLDLSVFKFNQDLEYLDLSHNQLQKISCHPI
TLR10_HUMAN 57 YNLLFQLQSSDFHSVSKLRVLILCHNRIQQLDLKTFEFNKELRYLDLSNNRLKSVTWYLL
A5YBP4_CHICK 117 ARLRHLDLSFNKFTVLPICQEFGIMFHLEYLGLSAMMIRRSDFRYVAHLQLDTVFLTLED
NP_001297353.1 117 ACLRHLDLSFNNFTVLPICQEFGTMFHLEYLGLSATMIRRSDFRYITHLQLNTVFLTLEN
XP_013041417.1 117 SCLRHLDLSFNNFTVLPICQEFGTMFHLEYLGLSAMMIRRSDFRHITHLQLNTIFLTLEK
TLR1_HUMAN 114 VNLKHLDLSFNAFDALPICKEFGNMSQLKFLGLSTTHLEKSSVLPIAHLNISKVLLVLGE
TLR6_HUMAN 121 VSFRHLDLSFNDFKALPICKEFGNLSQLNFLGLSAMKLQKLDLLPIAHLHLSYILLDLRN
TLR10_HUMAN 117 AGLRYLDLSFNDFDTMPICEEAGNMSHLEILGLSGAKIQKSDFQKIAHLHLNTVFLGFRT
A5YBP4_CHICK 177 FSL--YEPLSLTALNTRSLHIVFATNQNFNFSLLYDGMSTSEKLKIVNLRYTLSHK---D
NP_001297353.1 177 FSL--YKPQSLTALNTKSLHIVFSANQNFNFSLLYDGMSTSENLKIVNVRYTLSYK---D
XP_013041417.1 177 FSL--YEPQSLTALNTKSLHIVFSENQNFNFSLLYDGMSTSENLKIVNIRYTLSYK---D
TLR1_HUMAN 174 TYGEKEDPEGLQDFNTESLHIVFPTNKEFHFI-LDVSVKTVANLELSNIKCVLEDNKCSY
TLR6_HUMAN 181 YYIKENETESLQILNAKTLHLVFHPTSLFAIQ-VNISVNTLGCLQLTNIKLNDD--NCQV
TLR10_HUMAN 177 LPH--YEEGSLPILNTTKLHIVLPMDTNFWVL-LRDGIKTSKILEMTNIDGKSQFV---S
A5YBP4_CHICK 232 FPSPSLELQKKIKATDLMLDTVDLEWTVILQIFLLVWDSSVEHLTVRNLIFRGPVVELTE
NP_001297353.1 232 FPSPALTLLKKIKTTALMLDTVDLEWPIILQIFLLIWYSPVEHLTVRNLIFRGPIGGLTA
XP_013041417.1 232 FPSPSLKLLKKIKTTTLMLDTVDLEWPIILQIFLLIWYSPVENLTVRNLTFRGPMGVLTP
TLR1_HUMAN 233 FLSILAKLQTNPKLSNLTLNNIETTWNSFIRILQLVWHTTVWYFSISNVKLQGQLD-F--
TLR6_HUMAN 238 FIKFLSELTRGSTLLNFTLNHIETTWKCLVRVFQFLWPKPVEYLNIYNLTIIESIR-E--
TLR10_HUMAN 231 YEMQRNLSLENAKTSVLLLNKVDLLWDDLFLILQFVWHTSVEHFQIRNVTFGGKAY-L--

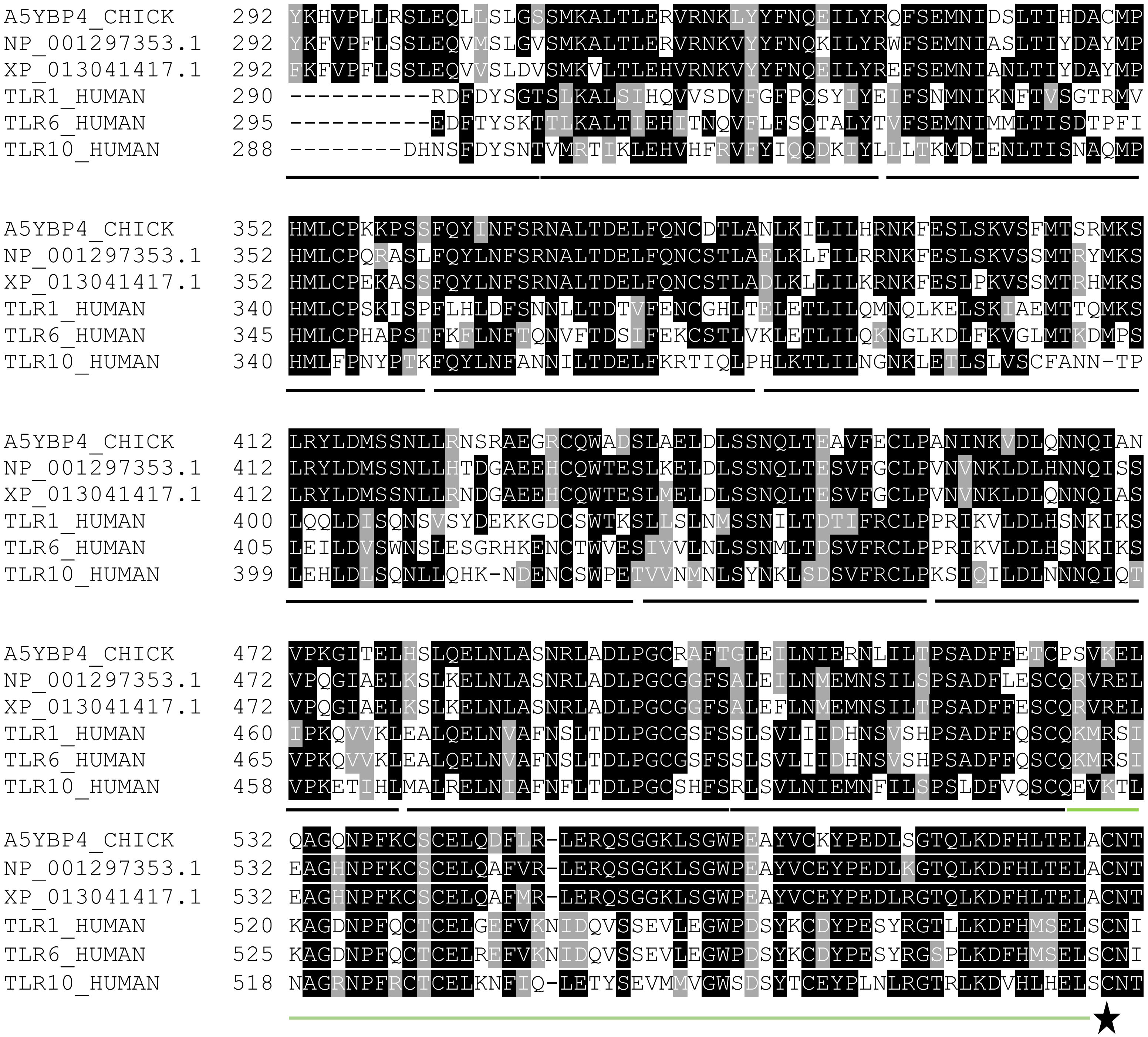
A5YBP4_CHICK 292 YKHVPLLRSLEQLLSLGSSMKALTLERVRNKLYYFNQEILYRQFSEMNIDSLTIHDACMP
NP_001297353.1 292 YKFVPFLSSLEQVMSLGVSMKALTLERVRNKVYYFNQKILYRWFSEMNIASLTIYDAYMP
XP_013041417.1 292 FKFVPFLSSLEQVVSLDVSMKVLTLEHVRNKVYYFNQEILYREFSEMNIANLTIYDAYMP
TLR1_HUMAN 290 ----------RDFDYSGTSIKALSIHQVVSDVFGFPQSYIYEIFSNMNIKNFTVSGTRMV
TLR6_HUMAN 295 ----------EDFTYSKTTLKALTIEHITNQVFLFSQTALYTVFSEMNIMMLTISDTPFI
TLR10_HUMAN 288 --------DHNSFDYSNTVMRTIKLEHVHFRVFYIQQDKIYLLLTKMDIENLTISNAQMP
A5YBP4_CHICK 352 HMLCPKKPSSFQYINFSRNALTDELFQNCDTLANLKILILHRNKFESLSKVSFMTSRMKS
NP_001297353.1 352 HMLCPQRASLFQYLNFSRNALTDELFQNCSTLAELKLFILRRNKFESLSKVSSMTRYMKS
XP_013041417.1 352 HMLCPEKASSFQYLNFSRNALTDELFQNCSTLADLKLLILKRNKFESLPKVSSMTRHMKS
TLR1_HUMAN 340 HMLCPSKISPFLHLDFSNNLLTDTVFENCGHLTELETLILQMNQLKELSKIAEMTTQMKS
TLR6_HUMAN 345 HMLCPHAPSTFKFLNFTQNVFTDSIFEKCSTLVKLETLILQKNGLKDLFKVGLMTKDMPS
TLR10_HUMAN 340 HMLFPNYPTKFQYLNFANNILTDELFKRTIQLPHLKTLILNGNKLETLSLVSCFANN-TP
A5YBP4_CHICK 412 LRYLDMSSNLLRNSRAEGRCQWADSLAELDLSSNQLTEAVFECLPANINKVDLQNNQIAN
NP_001297353.1 412 LRYLDMSSNLLHTDGAEEHCQWTESLKELDLSSNQLTESVFGCLPVNVNKLDLHNNQISS
XP_013041417.1 412 LRYLDMSSNLLRNDGAEEHCQWTESLMELDLSSNQLTESVFGCLPVNVNKLDLQNNQIAS
TLR1_HUMAN 400 LQQLDISQNSVSYDEKKGDCSWTKSLLSLNMSSNILTDTIFRCLPPRIKVLDLHSNKIKS
TLR6_HUMAN 405 LEILDVSWNSLESGRHKENCTWVESIVVLNLSSNMLTDSVFRCLPPRIKVLDLHSNKIKS
TLR10_HUMAN 399 LEHLDLSQNLLQHK-NDENCSWPETVVNMNLSYNKLSDSVFRCLPKSIQILDLNNNQIQT
A5YBP4_CHICK 472 VPKGITELHSLQELNLASNRLADLPGCRAFTGLEILNIERNLILTPSADFFETCPSVKEL
NP_001297353.1 472 VPQGIAELKSLKELNLASNRLADLPGCGGFSALEILNMEMNSILSPSADFLESCQRVREL
XP_013041417.1 472 VPQGIAELKSLKELNLASNRLADLPGCGGFSALEFLNMEMNSILTPSADFFESCQRVREL
TLR1_HUMAN 460 IPKQVVKLEALQELNVAFNSLTDLPGCGSFSSLSVLIIDHNSVSHPSADFFQSCQKMRSI
TLR6_HUMAN 465 VPKQVVKLEALQELNVAFNSLTDLPGCGSFSSLSVLIIDHNSVSHPSADFFQSCQKMRSI
TLR10_HUMAN 458 VPKETIHLMALRELNIAFNFLTDLPGCSHFSRLSVLNIEMNFILSPSLDFVQSCQEVKTL
A5YBP4_CHICK 532 QAGQNPFKCSCELQDFLR-LERQSGGKLSGWPEAYVCKYPEDLSGTQLKDFHLTELACNT
NP_001297353.1 532 EAGHNPFKCSCELQAFVR-LERQSGGKLSGWPEAYVCEYPEDLKGTQLKDFHLTELACNT
XP_013041417.1 532 EAGHNPFKCSCELQAFMR-LERQSGGKLSGWPEAYVCEYPEDLRGTQLKDFHLTELACNT
TLR1_HUMAN 520 KAGDNPFQCTCELGEFVKNIDQVSSEVLEGWPDSYKCDYPESYRGTLLKDFHMSELSCNI
TLR6_HUMAN 525 KAGDNPFQCTCELREFVKNIDQVSSEVLEGWPDSYKCDYPESYRGSPLKDFHMSELSCNI
TLR10_HUMAN 518 NAGRNPFRCTCELKNFIQ-LETYSEVMMVGWSDSYTCEYPLNLRGTRLKDVHLHELSCNT

```
A5YBP4_CHICK     591 TLLIVTALLL--TLVLVAVVAFLCIYLDVPWYVRMLWQWTQTKRRAWHDCPEERETALQF
NP_001297353.1   591 TLLIVTALLLLLTLVLVGVVAFLCIYLDVPWYVRMLWQWTQTKRRAWHECPEERETVLQF
XP_013041417.1   591 TLLIVTALLL--TLVLVGVVAFLCIYLDVPWYVRMLWQWTQTKRRAWHECPEERETVLQF
TLR1_HUMAN       580 TLLIVTIVAT--MLVLAVTVTSLCSYLDLPWYLRMVCQWTQTRRRARNIPLEELQRNLQF
TLR6_HUMAN       585 TLLIVTIGAT--MLVLAVTVTSLCIYLDLPWYLRMVCQWTQTRRRARNIPLEELQRNLQF
TLR10_HUMAN      577 ALLIVTIVVI--MLVLGLAVAFCCLHFDLPWYLRMLGQCTQTWHRVRKTTQEQLKRNVRF

A5YBP4_CHICK     649 HAFISYSERDSLWVKNELIPNLEKGEGCIQLCQHERNFIPGKSIVENIINCIEKSYKSIF
NP_001297353.1   651 HAFISYSERDSVWVKTELIPNLEKGEGSVRLCQHERNFVPGKSIVENIINCIDKSYKSIF
XP_013041417.1   649 HAFISYSERDSVWVKNELIPNLEKGEGSVQLCQHERNFVPGKSIVENIINCIDKSYKSIF
TLR1_HUMAN       638 HAFISYSGHDSFWVKNELLPNLEKEG--MQICLHERNFVPGKSIVENIITCIEKSYKSIF
TLR6_HUMAN       643 HAFISYSEHDSAWVKSELVPYLEKED--IQICLHERNFVPGKSIVENIINCIEKSYKSIF
TLR10_HUMAN      635 HAFISYSEHDSLWVKNELIPNLEKEDGSILICLYESYFDPGKSISENIVSFIEKSYKSIF

A5YBP4_CHICK     709 VLSPNFVQSEWCHYELYFAHHKLFSENSNSLILILLEPIPPYVIPARYHKLKALMAKRTY
NP_001297353.1   711 VLSPNFVQSEWCHYELYFAHHKLFSENCNSLILILLEPIPQYIIPARYHKLKALMAKRTY
XP_013041417.1   709 VLSPNFVQSEWCHYELYFAHHKLFSENCNSLILILLEPIPQYIIPARYHKLKALMAKRTY
TLR1_HUMAN       696 VLSPNFVQSEWCHYELYFAHHNLFHEGSNSLILILLEPIPQYSIPSSYHKLKSLMARRTY
TLR6_HUMAN       701 VLSPNFVQSEWCHYELYFAHHNLFHEGSNNLILILLEPIPQNSIPNKYHKLKALMTQRTY
TLR10_HUMAN      695 VLSPNFVQNEWCHYEFYFAHHNLFHENSDHIILILLEPIPFYCIPTRYHKLKALLEKKAY

A5YBP4_CHICK     769 LEWPKERSKHALFWANLRAAISINLSVADEQNRTEV-----------------------
NP_001297353.1   771 LEWPKERSKRALFWANLRAAINVNLPMSFEGNEEEN---------DVTFTDSITQPLIK
XP_013041417.1   769 LEWPKERSKRALFWANLRAAININLPKSFEANEEEN---------DVTSTGSITQPLIK
TLR1_HUMAN       756 LEWPKEKSKRGLFWANLRAAINIKLTEQAK-----K-----------------------
TLR6_HUMAN       761 LQWPKEKSKRGLFWANIRAAFNMKLTIVTENNDVKS-----------------------
TLR10_HUMAN      755 LEWPKDRRKCGLFWANLRAAINVNVLATREMYELQTFTELNEESRGSTISLMRTDCL--
```

Figure S1: The protein sequences of chTLR1t1, duTLR1t1, goTLR1, hTLR1, hTLR6, and hTLR10 aligned using the ClustalW program. Conserved amino acids and synonymous substitutions are shadowed in black and gray, respectively. The cytoplasmic (TIR) domain, the TM region, the extracellular domain with the conserved cysteine-rich CT region (LRR-CT), LRR1–19 and signal peptide are indicated below the sequence in orange, grey, blue, light green, black and orange respectively. Conserved proline and glycine in the BB loop of TIR domain that facilitates interaction with TLR2 to promote signaling are highlighted with black triangles. The asterisk at position 588 marks the conserved cysteine residue.

```
NP_001032924.1    1 MRILIGSLYFYFISFLFSKVNGFLTQRTSPVSSFPFYNYSYLNLSSVSQAQAPKTARALN
XP_005018927.2    1 MGILIRSLHFYFISFLLSRANGFLTWRT-TESAFPFYNYSYLNLSSVSEAQAPKMARALN
XP_013046493.1    1 MGILIRSLHFYFTSFLFSRANGFLTWRTPTESAFPFYNYSYLNLSSVSQAQAPKMARALN

NP_001032924.1   61 FSYNAIEKITKRDFEGFHVLEVLDLSHNHIKDIEPGAFENLLSLVSVDLSFNDKNLLVSG
XP_005018927.2   60 FSHNIIEKITKRDFEGFDALEVLDLSYNQIKDIEPGTFEILLSLVSVNLSFNDKKLLVSG
XP_013046493.1   61 FSHNIIEKITKRDFEGFDALEVLDFSYNRIKDIEPSTFETMLSLVSVNLTFNDKKLLVSG

NP_001032924.1  121 LAPHLKLIPTSGASGPSQIYMYFQKSAEAALEPSAPAELLPHLEDPPNPGNVNPRFRQ--
XP_005018927.2  120 LAPHLKLLATSRAS---------DKSTEAALEPPASAEELPHSGSPPDLQKINLRLRRST
XP_013046493.1  121 LAPHLKLLATSRAS---------EKSTEAALEPPASAEELPHSGSPPDLQKVNMRLRRST

NP_001032924.1  179 ---RRTEENKTSPPAATLRPDLCGAPINGLLDLSRTKLSNEELTAKLDADLCQAQLGTVL
XP_005018927.2  171 RHLRRAEENTMVSPTATLRPNRCGLPINGTLDLSKSKLSEEELTEKLDPDLCQAQLDGVL
XP_013046493.1  172 RHLPRAEENTTVSPTATLRPNRCGLPINGTLDLSKSKLSEEELMEKLDPDLCQAQLDSVL

NP_001032924.1  236 EFNISHSDLEMDLLSLFILFLPMKDIQSVDASYNRITINNIDVEAICHFPFSNFSFLNIS
XP_005018927.2  231 ELDISHNDLEMDLLSLFILFLPMKNVQSIDVSYNNITINNIDVEAICRFPFSNLSFVNIS
XP_013046493.1  232 ELDISHNDLEMDLLSLFILFLPMKNVQSIDASYNNITIDNIDVEAICRFPFSNLSFVNIS

NP_001032924.1  296 NNPINSLETVCLPASITVIDLSFTNISTIPANFAKKLSKLERMYVQGNQLIYTVRPENPS
XP_005018927.2  291 NNPLNNLETICLPPTITVIDLSFTNISVIPQNFAKKLFNLENMYVQGNHFIYTVHPKPNN
XP_013046493.1  292 NNPLNNLETICLPPTITVIDLSFANVSVIPQNFAKKLFNLENMYVQGNHFIYTAHPKPTN

NP_001032924.1  356 ATPRPPPGTVQISAISLVRNQAGTPIESLPESVKHLKVSNCSIVELPEWFANRMQELLFL
XP_005018927.2  351 TAPKFAPGTVHISAISLVRNQAGTPIESLPEKVKHLKISNCSIVELPEWFANRMQELLFL
XP_013046493.1  352 TAPKFAPGTVHISAISFVRNQAGTPIESLPEKVKHLKISNCSIVELPEWFANRMQELLFL

NP_001032924.1  416 DLSSNRISMLPDLPISLQQLDISNSDIKIIPPRFKSLSNVTVFNIQNNKLTEMHPEYFPS
XP_005018927.2  411 DLSSNRISMLPDLPISLQHLDISNSDIKIIPPSFKSLPNLTVFNIQNNKVTDMHPEYFPL
XP_013046493.1  412 DLSSNRISMLPDLPVSLQHLDISNSDIKIIPPSFKSLSNLTVFNIQNNKVTDMHPEYFPL
```

```
NP_001032924.1  476 TLTTCDISKNKLKVLSLTKALENLESLNVSGNLITRLEPACQLPSLTNLDSSHNLISELP
XP_005018927.2  471 TLTKCDISKNKLSVLSLTKTIGKLEFLNVSRNLITRLEPTSQLRLLTNLDGSHNLISELP
XP_013046493.1  472 TLTKCDISKNKLNVLSLTKTIGKLEFLNVSRNLITRLEPTSQFHLLTNLDGSHNLISELP

NP_001032924.1  536 DHLGQSLLMLKHFNLSGNKISFLQRGSLPASLEELDISDNAITTIVQDTFGQLTSLSVLT
XP_005018927.2  531 DHFGKSLPMLKYFNLSGNKISFLQRGSLPVSLMELDISDNAITTIAEDTFGQLTSLSILT
XP_013046493.1  532 DRFGKSFPMLKYFNLSGNKISFLQRGSLPVSLMELDISNNAITTIVEDTFGQLTSLSILT

NP_001032924.1  596 VQGKHFFCNCDLYWFVNIYIRNPHLQINGKDDLRCSFPPDRRGSLVKSSNLTLLHCSLGI
XP_005018927.2  591 VQGKHFFCNCDLYWFVNVYVHTPHLQINGKENLRCSFPPDRRGSLVEGSNLTLLHCSLGI
XP_013046493.1  592 VQGKHFFCNCDLYWFVNVYIHIPYLQINGKENLRCSFPPDRRGSLVEGSNLTLLHCSLGI

NP_001032924.1  656 QMAITACMAILVVLVLTGLCWRFDGLWYVRMGWYWCMAKRRQYKKRPENKPFDAFI-SYS
XP_005018927.2  651 QMAITACVAVLVVLVLTGLCWRFDGLWYVKMGWYWCMAKRKQYKKRPENKPFDAF-ISYS
XP_013046493.1  652 QMAITACVAVLVVLVLTGLCWHFDGLWYVRMGWYWCTAKRKQYKKKPENKPFDAFDASYS

NP_001032924.1  715 EHDADWTKEHLLKKLETDGFKICYHERDFKPGHPVLGNIFYCIENSHKVLFVLSPSFVNS
XP_005018927.2  710 EHDANWMKENLLVRLETDGFKICYHERDFKPGHPVLGNIFNCIENSHKVLFVLSPSFVNS
XP_013046493.1  712 EHDAHWTKENLLERLETDGFKICYHERDFKPGHPVLGNIFYCIENSHKVLFVLSPSFVNS

NP_001032924.1  775 CWCQYELYFAEHRVLDENQDSLIMVVLEDLPPDSVPQKFSKLRKLLKRKTYLKWSPEEHK
XP_005018927.2  770 CWCQYELYFAEHQVLNENQDSLIMIVLEDLPPNSIPQKFSKLRKLLKRKTYLKWSPEEHK
XP_013046493.1  772 CWCQYELYFAEHRVLNENQDSLIMIVLEDLPLNSVPQKFSKLRKLLKRKTYLKWSPEEHK

NP_001032924.1  835 QKIFWHQLAAVLKTTNEPL-VRAENGPNEDVIEME
XP_005018927.2  830 QKIFWHQLAAVLKTTNEPFIVRAENGPTQDMYEME
XP_013046493.1  832 QKIFWHQLAAVLKTTNERFVVRAENGSTQDMYEME
```

Figure S2: Schematic alignment of TLR15 protein sequences of chicken, duck and goose. Depicted are the predicted signal sequence, the N-terminal LRR (LRR-NT), 19 LRRs, the C-terminal LRR (LRR-CT), the transmembrane domain (TM), and the TIR domain in blue, red, black, orange, green and purple underlining respectively. The proline-rich loop (aa 359–363) is boxed in black.

```
XP_004948095.1    1 MSWAMQYHGCLPWGSWT---RLCGSQPSRRAARCVYLCTASGPGAAGSCFHRKILHIGG-
XP_027300205.1    1 MSRAVQGRGCLPWACLG-----------------PYLRAASLPGAAGGSFHGKILSGAS-
XP_013042726.1    1 ---------------------------------------------AGGSFRGKILSGDS-
NP_001269072.1    1 MRWGHH----LPRASWGSGFRRALQRPDDR---IPFLIHWSWP-----------LQGERP

XP_004948095.1   57 TSLPSRSCVRYKGGSQENRAQRSSSHHGLDHLRNVASSDAIKKHQKSLSAWFSNQPNEER
XP_027300205.1   43 ISLP-RSCVRHQGGPQGPPAQRGSSRHGLDHLRTVASSDAIKKHQKSLAAWFSNQPSEER
XP_013042726.1   15 GSLP-RSCVRHQGGSQGPPAQRGSSRHGLDHLRTVASSDAIKKHQKSLAAWFSNQPNEER
NP_001269072.1   43 FGPP-RAFIRHHGSSVDSAP--PPGRHGR-LFPSASATEAIQRHRRNLAEWFSRLPREER

XP_004948095.1  117 QFGPSFSLDAIHVDPVIRESSLEQILKPSPDLTIQHQLQQPCRQVISLQNLFDVDACGRQ
XP_027300205.1  102 QFGPSFSLDAVHVDPVIRESSLEQILKPSPELTIRHQLQRSSRRVISLQNLFEADACGRQ
XP_013042726.1   74 QFGPSFSLDAVHVDPVIRESSLEQILKPSPELTIQHQLQRSSRQVISLQNLFDVDACGRQ
NP_001269072.1   99 QFGPTFALDTVHVDPVIRESTPDELLRPPAELALEHQPPQAGLPPLALSQLFNPDACGRR

XP_004948095.1  177 VKNVVLYGTVGTGKSTLIKKMVVDWCHGLLPRFELVIPFSCEDLSH-SHVPISLRRLITK
XP_027300205.1  162 VKNVVLYGTVGTGKSTLIKKMVVDWCHGLLPRFELVIPFSCEDLSH-SRVPISLRRLVTK
XP_013042726.1  134 VKNVVLYGTVGTGKSTLIKKMVVDWCHGLLPRFELVIPFSCEDLSH-SRVPISLRRLVTK
NP_001269072.1  159 VQTVVLYGTVGTGKSTLVRKMVLDWCYGRLPAFELLIPFSCEDLSSLGPAPASLCQLVAQ

XP_004948095.1  236 KYQHLRDVVPLLGSSNLKVLFILNGLERLNLDFRLAGTELCCDANEPVPPSAIVVNLLRK
XP_027300205.1  221 KYQHLRDVAPLLGASNLKVLFILNGLERLNLDFRLAGTELCCDPSEPIAPSAIVVNLLRK
XP_013042726.1  193 KYQHLRDVAPILGASNLKVLFILNGLERLNLDFRLAGTELCCDPGEPIAPSAIVVNLLRK
NP_001269072.1  219 RYTPLKEVLPLMAAAGSHLLFVLHGLEHLNLDFRLAGTGLCSDPEEPQEPAAIIVNLLRK

XP_004948095.1  296 YLLPEASIIVTTRPSAVRRIPGKYVGRYAEICGFSDTNLQKLYFQLRLSQPGCDGE---N
XP_027300205.1  281 YLLPEASIIVTARPSAVRRIPGKYVGRYAEICGFSDTNLQKLYFQMRLSQPGCDGE----
XP_013042726.1  253 YLLPEASIIVTTRPSAVRRIPGKYVGRYAEICGFSDTNLQKLYFQMRLSQPGCGGG---D
NP_001269072.1  279 YMLPQASILVTTRPSAIGRIPSKYVGRYGEICGFSDTNLQKLYFQLRLNQPYCGYAVGGS
```

```
XP_004948095.1  353 SVASRSGEQDNLVEMLSRNLERQNQIAAACFLPSYCWLVCTTLHFLYFTRTVPPSQTLSG
XP_027300205.1  337 ----GSGEQENLVEMLWRNLERQNQIAAACFLPSYCWLVCTTLHFLYFTRTVPPSQTLTG
XP_013042726.1  310 G--EGSGEQDNLVEMLSRNLEHQNQIAAACFLPSYCWLVCTTLHFLYFTRTVPPSQTLTG
NP_001269072.1  339 GVSATPAQRDHLVQMLSRNLEGHHQIAAACFLPSYCWLVCATLHFLHA--PTPAGQTLTS

XP_004948095.1  413 IYTSFLRLNFSGEVLDSTDPTKISMMKYVAKTVGKLAHEGVMSRKTSFTEEDLQQCFEVE
XP_027300205.1  393 IYTSFLRLNFSGEVLDSSEPTAVSMMKYVAKTVGKLAHEGVMSRQTCFSEEDLRQCFEVE
XP_013042726.1  368 IYTSFLRLNFSGEVLDSTDPTDISMMKYVAKTVGKLAHEGVMSRKTCFSEEDLRQCFEVE
NP_001269072.1  397 IYTSFLRLNFSGETLDSTDPSNLSLMAYAARTMGKLAYEGVSSRKTYFSEEDVCGCLEAG

XP_004948095.1  473 MKTESELNQLEVFRSDVFRFFLTPCVQPGKEHTFVFTIPAMQEYLAALYVVLGEKKTLVQ
XP_027300205.1  453 MKTESELNLLEVFRSDVFPFFLTPCVQPGREHTFVFTIPAMQEYLAALYVVLGEKKTLAQ
XP_013042726.1  428 MKTESELNLLEVFRSDVFRFFLTPCVQPGKEHTFVFTIPAMQEYLAALYVVLGEKKTLAQ
NP_001269072.1  457 IRTEEEFQLLHIFRRDALRFFLAPCVEPGRAGTFVFTVPAMQEYLAALYIVLGLRKTTLQ

XP_004948095.1  533 KVGKEVSEIIGKVSEDAAVVLSIISKVLPLRFLPVLFNLLKMFPRFFSRLSGKGRDTIAR
XP_027300205.1  513 KVGKELSELIGKVSEDAAVVLGIVSKVLPLRFLPVLFNLLKIFPRFFSRLSGKDRDTIAR
XP_013042726.1  488 KVGKELSELIGKVSEDAAVVLGIVSKVLPLRFLPVLFNLLKIFPRFFSRLSGKDRDTIAR
NP_001269072.1  517 KVGKEVAELVGRVGEDVSLVLGIMAKLLPLRALPLLFNLIKVVPRVFGRMVGKSREAVAQ

XP_004948095.1  593 TMAEELFKEEDYYNDDVLDQINSSILGVEGPMRHPDEAADDEVFELFPIFMGGILSRRNR
XP_027300205.1  573 TMAEELFKEEDYYNDDVLDQINSSILGVEGPLRHPDEAPDDEVFELFPIFMGGLLSRRNR
XP_013042726.1  548 TMAEELFKEEDYYNDDVLDQINSSILGVEGPMRHPDEAPDDEVFELFPIFMGGLLSRRNR
NP_001269072.1  577 AMVLEMFREEDYYNDDVLDQMGASILGVEGPRRHPDEPPEDEVFELFPMFMGGLLSAHNR

XP_004948095.1  653 AILEQLGCSIKNLAAFEIAKAMKKTVIRNSRKGLPPSELMDYLFFLHEFQNERFTAEAVR
XP_027300205.1  633 ALLEQLGCSIKNLAALEIAEAMKKTVIRNSRKGLPPSELMDYLFFLHEFQNERFTAEAVR
XP_013042726.1  608 AILEQLGCSIKNLAAFEIAEAMKKTVIRNSRKGLPPSELMDYLFFLHEFQNERFTAEAVR
NP_001269072.1  637 AVLAQLGCPIKNLDALENAQAIKKKLGKLGRQVLPPSELLDHLFFHYEFQNQRFSAEVLS
```

```
XP_004948095.1  713 SLRTVNLSSVKMTPLKCCVLASVMGSTSHEVEELNLTSCNLDASSLRTLFPVLLRCKFLH
XP_027300205.1  693 SLRTVNLSSVKLTPLKCSVLASVLGAACHEVDELNLTSCNLDAASLRTLFPVLLRCKVLH
XP_013042726.1  668 SLRTVNLSSVKLTPLKCSVLASVMGTACHEVDELNLTSCNLDAGSLRTLFPVLLRCKVLQ
NP_001269072.1  697 SLRQLNLAGVRMTPVKCTVVAAVLGSGRHALDEVNLASCQLDPAGLRTLLPVFLRARKLG

XP_004948095.1  773 LQLNSLGPDACKEIRDLLLHDKCAVSNLRLGNNPIGEQGAQYLAEALAGNRSLTHLSLLH
XP_027300205.1  753 LQLNSLGPDACEELRALLLHAKCAVSDLRLSNNPVGVQGARHLAEALAGNRSLSRLSLLH
XP_013042726.1  728 XVPLLLPLGSAQGLGS---PRLCR-GTERAGKGRNALSVLRAVPEDYSARRRSSPRSLLH
NP_001269072.1  757 LQLNSLGPEACKDLRDLLLHDQCQITTLRLSNNPLTAAGVAVLMEGLAGNTSVTHLSLLH

XP_004948095.1  833 TALGDRGVELLAQHLAENQQLQELNLGYNSLTDTAALHVVEVAKKHATLDKVHLYFNDIS
XP_027300205.1  813 AALGDRGAEEIARRLPENQQLQELNLGYNSLTDASALSVVEVAKKHATLDKVHLYFNDIS
XP_013042726.1  784 AALGDRGVEEIAGRLADNQQLQELNLGYNALTDAAALRVVEVAKKHATLDKVHLYFNDIS
NP_001269072.1  817 TGLGDEGLELLAAQLDRNRQLQELNVAYNGAGDTAALALARAAREHPSLELLHLYFNELS

XP_004948095.1  893 EDGKRALDSLRMDR-DGVRALVFLTAGTDVSDYWSSILNVVHKNLPFWDRERVRQHLTII
XP_027300205.1  873 EEGKRALHSLRMER-DGVRALVFLTAGTDVSDYWSNILSTVQRNLPFWDRERVRQHLALL
XP_013042726.1  844 EEGKRALHSLRMDR-DGVRALVFLTAGTDVSDYWSSILNTVHRNLPFWDRERVRQHLALL
NP_001269072.1  877 SEGRQVLRDLGGAAEGGARVVVSLTEGTAVSEYWSVILSEVQRNLNSWDRARVQRHLELL

XP_004948095.1  952 LQDLESSRRQTVNPWRKAKFLRVESEVKKMLGKLQHGTL
XP_027300205.1  932 LQDLESSRRQTGNPWRKAKFLRVESEVKRMLAKLQNGTL
XP_013042726.1  903 LQDLESSRRQTANPWRKAKFLRVESEVKRMLGKLQQGTL
NP_001269072.1  937 LRDLEDSRGATLNPWRKAQLLRVEGEVRALLEQLGSSGS
```

Figure S3: Schematic alignment of NLRX1 protein sequences of chicken, duck goose and human comprising the N-terminal X domain, NACHT, and the C-terminal LRR. The LC3-interacting region is highlighted in grey.

```
XP_025006433.1     1 ------------------------------------------------------------
XP_005030015.2     1 MRADRRGNPAGSLLRAFQGGLQFSCRDRSAAATSSRLYHFRDTAGSGFPCPEPALPPEQH
XP_013053801.1     1 ------------------------------------------------------------
AAI43363.1         1 ------------------------------------------------------------

XP_025006433.1     1 ------------------------------------------MAGEES--TILLEALEGL
XP_005030015.2    61 QVPSLHPGAAASPEAGSKEPGRGEASLAHRLEAAAGDFPHPVMAGEGSAFTVLLGALEGL
XP_013053801.1     1 -----------------------------------------MMAGEGSAFTVLLAALEGL
AAI43363.1         1 ------------------------------------------MKM-ASTRCKLARYLEDL

XP_025006433.1    17 TLEDFQEFKKKLPHT-DIKGGWNIGRDELEKVTHPSSLISYMGDSYGEGAAMDIAISLFE
XP_005030015.2   121 TLEGFQEFKTKLSHV-HTKRGWNIPEDALVEATHPSTLVNCMGKSYGEDAAMDIAIGLFE
XP_013053801.1    20 TLEGFQEFKTKLSHV-HTKGGWNIPEDVLVEATHPSTLVNCMGNSYGEDAAVDIAIGLFE
AAI43363.1        18 EDVDLKKFKMHLEDYPPQKGCIPLPRGQTEKADHV-DLATLMIDFNGEEKAWAMAVWIFA

XP_025006433.1    76 EMNQRDLAEKILDEK-----------------------------------------VKEY
XP_005030015.2   180 EMNQRDLAEKILDEK-----------------------------------------VKEY
XP_013053801.1    79 EMNQRDLAEKILDEK-----------------------------------------VKEY
AAI43363.1        77 AINRRDLYEKAKRDEPKWGSDNARVSNPTVICQEDSIEEEWMGLLEYLSRISICKMKKDY

XP_025006433.1    95 KQKYTEHVAREFLQYKEANSCLGENLSVRDRYTNLTIARKSWDQHGDEPGD--V--S---
XP_005030015.2   199 KQKYREHVAREFLQYKEVNSCLGENLSVSSRYTALTITKKPWSQRGGEPEGADV--SWGC
XP_013053801.1    98 KQKYREHVAREFLRYKELNSCLGENLSVSSRYTALTIAKKPWSQSGGEPEGADV--SWGC
AAI43363.1       137 RKKYRKYVRSRFQCIEDRNARLGESVSLNKRYTRLRLIKEHRSQQEREQELLAIGKTKTC

XP_025006433.1   148 ---SDTVTTQTLLEPSKDGQVP-PITVLVGASGMGKTMTIRKVMMEWVEGTLCT-QFDYV
XP_005030015.2   257 ADTTSAVTAQTLFKPDEDGQTP-QTVVLVGAPGMGKTMMVRKMMVEWVEGALYM-QFDYV
XP_013053801.1   156 ADTTTAVTVQTLFKPDEDGQTP-QTVVLVGAPGMGKTMMVRKMMVEWVEGALYM-QFDYV
AAI43363.1       197 ESPVSPIKMELLFDPDDEHSEPVHTVVFQGAAGIGKTILARKMMLDWASGTLYQDRFDYL
```

```
XP_025006433.1  203 FCIDCKELSFSKEVSMVDLISKCCPQQRMPAGRILGNPEKILFIFDSFEALGLPLAQPKD
XP_005030015.2  315 FCIDCKELSLSKQVSVLDLVSKCCPHQRIPAGSILDNQEKVLFIFDGFEALGFPLAQPKD
XP_013053801.1  214 FCIDCKELSLSKQVSVLDLVSKCCPHQRIPAGSILDNQEKVLFIFDGFEALGFPLAQPKD
AAI43363.1      257 FYIHCREVSLVTQRSLGDLIMSCCPDPNPPIHKIVRKPSRILFLMDGFDELQGAFDEHIG

XP_025006433.1  263 ELSTDPTEAKPLETTLLSLLRRTVLPESSVLIATRPAALQSLGQCLEGKHYVEILGFSPA
XP_005030015.2  375 ELSSDPREAKPLETTLMSLLKRTVLPASSLLITTRPMALQNLGRCLEGECYVEILGFSAA
XP_013053801.1  274 ELSSDPREAKPLETTLMSLLKRTVLPASSLLITTRPTALQNLGQCLEGECYVEILGFSAA
AAI43363.1      317 PLCTDWQKAERGDILLSSLIRKKLLPEASLLITTRPVALEKLQHLLDHPRHVEILGFSEA

XP_025006433.1  323 AREEYFHRYFGNDSKADVAFRFTRGNEVLYSLCVIPVMSWTVCTVLERELYERNQLLACS
XP_005030015.2  435 AREEYFHRYFKNDNKADVAFRFARGNESLYSLCVIPIMSWTICTILEQELYKKNNILECS
XP_013053801.1  334 AREEYFHRYFKNDNKADVAFRFTRGNETLYSLCVIPIMSWTICTILEQELYKKNNLLECS
AAI43363.1      377 KRKEYFFKYFSDEAQARAAFSLIQENEVLFTMCFIPLVCWIVCTGLKQQMESGKSLAQTS

XP_025006433.1  383 KTTTQMIMFYLSWLMKHRVSNAWQNLQQFLHKLCSLAADGIWKHKVLFEEKEIEDQGLNQ
XP_005030015.2  495 KATTWMGMFYLSWLMKCRGSNAQQDLQQFLHKLCSLAADGIWKHKVLFEEKEVKDCGLDW
XP_013053801.1  394 KATTWMGMFYLSWLMKCRGSNAQQYLQQFLRRLCSLAADGIWKHKVLFEEREIKDCGLDW
AAI43363.1      437 KTTTAVYVFFLSSLLQPRGGSQEHGLCAHLWGLCSLAADGIWNQKILFEESDLRNHGLQK

XP_025006433.1  443 PQLLSLFLNEKGLEKGTDHVNVYSFSHLHLQELFAAMFYVLEDQDGMVSDS-----RILA
XP_005030015.2  555 PDLLSLFLNEKSLKKGIDQDNVYSFTHLHLQEFFAAMFYVLDDDEETVSDP-----EALK
XP_013053801.1  454 PDLLSLFLNEKSLKKGVDQGNVYSFTHLHLHEFFAAMFYVLDDDEETVSDP-----EALA
AAI43363.1      497 AD-VSAFLRMNLFQKEVDCEKFYSFIHMTFQEFFAAMYYLLEEEKEGRTNVPGSRLKLPS

XP_025006433.1  498 KDVNMLLESYHTSRMD-LNVTVRLLFGLVNPKSVEYAGEGIGCRISLQPQEDLLRWLQTR
XP_005030015.2  610 KNVNTLLESYSKSRKD-LNLTIRFLFGLVNPKSIEYAGERIGCRISPRAQEDLLRWLQTR
XP_013053801.1  509 KNVNTLLESYSKSRKD-LNLTIRFLFGLVNPKSIEYAGERIGCRISPRAREDLLRWLQTR
AAI43363.1      556 RDVTVLLENYGKFEKGYLIFVVRFLFGLVNQERTSYLEKKLSCKISQQIRLELLKWIEVK
```

```
XP_025006433.1  557 PRGTSHPREVMKIEDLDTFHLLFETNEKSFVQSVLGSFTGIALQDVKLTLYDQAALCFCI
XP_005030015.2  669 HRGLSHPSEALMIKDLDTFHFLFEMNEKSFAQNVLGCFTGIDLHDIKLTLYDQMALCFCI
XP_013053801.1  568 YRGLSHRSEVMMIKELDTFHFLFEMNEKSFAQKVLGCFTGIDLHDIKLTPYDQMALCFCI
AAI43363.1      616 AKAKK---LQIQPSQLELFYCLYEMQEEDFVQRAMDYFPKIEINL--STRMDHMVSSFCI

XP_025006433.1  617 KQWAGLLSVTLRSCSFHQQHHRQEPAKGLPRQSW--------------------------
XP_005030015.2  729 KQWDGVDSVTLRSCSFHQQQCREEPATVLPGQHP--------------------------
XP_013053801.1  628 KQWDGLDSVTLRSCSFRQQQCSEEPTTVLPWQHP--------------------------
AAI43363.1      671 ENCHRVESLSLGFLH---NMPKEEEEEEKEGRHLDMVQCVLPSSSHAACSHGLVNSHLTS

XP_025006433.1  651 ---------------------RQEELHSP-LHPLCQALGHPGSSLQSLRLQWCGLTEGDS
XP_005030015.2  763 ---------------------RQEELRSP-LHPLCQALGHTGSSLQNLRLQWCGLTEGGC
XP_013053801.1  662 ---------------------RQEELRSP-LHPLCQALGHAGSSLQNLRLQWCGLTEGGC
AAI43363.1      728 SFCRGLFSVLSTSQSLTELDLSDNSLGDPGMRVLCETLQHPGCNIRRLWLGRCGLSHECC

XP_025006433.1  689 GALGMLLATLPSLVHLELGDGALGDDGVRMLCAGLRQPGCQLRVLRLRYTHLTSACCQDL
XP_005030015.2  801 EALGTLLATHPSLACLELGDGALGDSGVRLLCTGLRQPGCHLRILRLRYTRLTSACCQDL
XP_013053801.1  700 KALGTLLATHPSLACLELGDGALGDSGVRLLCAGLRQPGCQLRILRLRYTRMTSACCQDL
AAI43363.1      788 FDISLVLSSNQKLVELDLSDNALGDFGIRLLCVGLKHLLCNLKKLWLVSCCLTSACCQDL

XP_025006433.1  749 AVALGTSTCLEELDLSFSA-----------------------------------------
XP_005030015.2  861 AAVLGTSPHLEELDLSFNT-----------------------------------------
XP_013053801.1  760 AAVLGTSPHLEELDLSFNT-----------------------------------------
AAI43363.1      848 ASVLSTSHSLTRLYVGENALGDSGVAILCEKAKNPQCNLQKLGLVNSGLTSVCCSALSSV

XP_025006433.1  768 ---------------GLRDDGVKLLCEGLQHSGCQLRVCRLGSCSLTGACCQALVAWLGH
XP_005030015.2  880 ---------------GLRDDGVQLLCEGLRHRACQLRVLRLGSCHLTGSCCQALATHIGE
XP_013053801.1  779 ---------------GLRDAGVQLLCEGLRDHACQLRVLRLGSCHLMGTCCQALATHLGE
AAI43363.1      908 LSTNQNLTHLYLRGNTLGDKGIKLLCEGLLHPDCKLQVLELDNCNLTSHCCWDLSTLLTS
```

```
XP_025006433.1  813 SHGLKCLDLSDTELGA-GATLLLQHLRHHSCTLQTLGLSTSTLSKDALQELAALRALKPS
XP_005030015.2  925 SCSLSCLDLSDTELGA-GAVLLLRQLRHPACPLQALGLSVSALNEDALQELVALRALKPS
XP_013053801.1  824 NCSLSCLDLSDTELGA-GAVLLLRQLRHPACPLQALGLSVSALNEDALQELVALRELKPS
AAI43363.1      968 SQSLRKLSLGNNDLGDLGVMMFCEVLKQQSCLLQNLGLSEMYFNYETKSALETLQEEKPE

XP_025006433.1  872 LKITDLLEHEAPETGAMAR-----------------------------------------
XP_005030015.2  984 LKIGDLLEHDTPQEGAMSR-----------------------------------------
XP_013053801.1  883 LKIGDLLEHDTPQELVASLQRPAAMDGSSGWACALCRHGQDDVAYLMPCLHQLCLGCVLR
AAI43363.1     1028 LTVV--FEP---------------------------------------------------

XP_025006433.1  891 --------------L-TFQRSVWAGRGAA-------------------------------
XP_005030015.2 1003 --------------L-PFQRGVWEGKGR--------------------------------
XP_013053801.1  943 WAKEKPSCPLYGGRLQSSKYSVWSADDYLECPIPELAEQLGDGQQDEQGAAGLVLRTPEH
AAI43363.1     1035 ------------------------------------------------------------

XP_025006433.1  905 -----------------------------------------------------VRGR---
XP_005030015.2 1016 -----------------------------------------------------LGVR---
XP_013053801.1 1003 SFPPQLWAAFFQEQLADARPLLAWLQEELRRIYGSQWWDVAVVQGIVLASLCIFGLDQQA
AAI43363.1     1035 ------------------------------------------------------------

XP_025006433.1  909 ------------------------------------------------------------
XP_005030015.2 1020 ------------------------------------------------------------
XP_013053801.1 1063 LVRQLQPSLHSHTVPFVALLVTVAAELYRTGVHWQRERRDARAAGGKEDSPAATPVTAEE
AAI43363.1     1035 ------------------------------------------------------------

XP_025006433.1  909 ---------------KGLPSSRAAPPHSRSLC-------------
XP_005030015.2 1020 ---------------KTLLSSRVAPPSNRNHC-------------
XP_013053801.1 1123 EGSHHRRGRDRQRQQGRLPG--AAGPAPTSLAGAGTTRLGGANIP
AAI43363.1     1035 --------------------------SW-----------------
```

Figure S4: Schematic alignment of NLRP3 protein sequences of chicken, duck goose and human comprising the N-terminal PYD domain, NACHT, and the C-terminal LRR.

```
XP_015150161.2    1 MGWWWVQAEVCTAVNTVLIAPGRRCHGTPLMEEAWISRYRRQLMRSISPQFLEEIIGYLQ
XP_027324583.1    1 ------------------------------MEEAWISRYQKQLVRSISPQFLEEIICHLR
XP_013032636.1    1 ------------------------------MEEAWINRYQKQLVRSISPQFLEEIICYLR
NP_849172.2       1 ----------------------------------------------------------MR

XP_015150161.2   61 RLDLLTVEEAGRAQEASSLPEQVRAVVDILAGKGSHASQTLQSFIETTNSQLYLHITVYE
XP_027324583.1   31 RLDLLTAEEAGRAQEASSLPEQVRAVVDVLAGKGSYASQCLQTFIETTNSQLYLHITVYE
XP_013032636.1   31 RLDLLTAEEAGRVQEASSLPEQVRAVVDVLAGKGSYASQCLQTFIETTNSQLYLHITVYE
NP_849172.2       3 KQEVRTGREAGQGHGTGSPAEQVKALMDLLAGKGSQGSQAPQALDRTPDAPLG--PCSND

XP_015150161.2  121 PMVQKHLESLQSFYGNSLEI-GSLQRLTNLLLVEGLTDIQQKEHDILQMEMTKGLRNVSK
XP_027324583.1   91 PMVQKHLESLQSRYGNGLET-GPVPRLMNLLLVEGLTDIQQKEHDILQIETTKGLRNVSK
XP_013032636.1   91 PMVQKHLESLQSYYGNGLET-GPLQRLTNLLLVEGLTDIQQKEHDILQIETTKGLRNVSK
NP_849172.2      61 SRIQRHRKALLSKVGGGPELGGPWHRLASLLLVEGLTDLQLREHDFTQVEATRGGGHPAR

XP_015150161.2  180 SIPLEKLFLPLSKVSIPPRISVTTGVAGIGKSTLVKLFVGRWTKGLINRDIMFVLPLTFR
XP_027324583.1  150 SIPLEKLFLPLSKVSIPPRISVTVGVAGIGKSTLVKLFVSSWTKGEITRDIMLGLPLTFR
XP_013032636.1  150 SIPLEKLFLPLSKVSIPPRISVTVGVAGIGKSTLVKLFVYSWAKGEINRDIMLVLPLTFR
NP_849172.2     121 TVALDRLFLPLSRVSVPPRVSITIGVAGMGKTTLVRHFVRLWAHGQVGKDFSLVLPLTFR

XP_015150161.2  240 ELNTYEKLSAERLIRSSFPHITEPNCISTGAARTLLILDGLDEFKTPLDFSNTVACTDPK
XP_027324583.1  210 ELNTYEKLSAERLIRLALPHATEPSCISAGAARVLLILDGLDEFKTPLDFSNTVVCTDPK
XP_013032636.1  210 ELNTYEKLSAERLIRLAFPHITEPSCISAGAARTLLILDGLDEFKTPLDFSNTVVCTDPK
NP_849172.2     181 DLNTHEKLCADRLICSVFPHVGEPSLAVAVPARALLILDGLDECRTPLDFSNTVACTDPK

XP_015150161.2  300 KEIQVDNLITNIIRGNLLQEASIWVTSRPTAASQIPGGLVDRMTEIRGFRATEMKDFLDQ
XP_027324583.1  270 KEIQVDNLITNIIRGNLLQEASVWVTSRPTAASQIPGGLVDRMTEIRGFGAAEMKEFLDQ
XP_013032636.1  270 KEIQVDNLITNIIRGNLLQEASVWVTSRPTAASQIPGGLVDRMTEIRGFGAAEMKEFLDQ
NP_849172.2     241 KEIPVDHLITNIIRGNLFPEVSIWITSRPSASGQIPGGLVDRMTEIRGFNEEEIKVCLEQ
```

```
XP_015150161.2  360 MFLDNKDLSGQVLHHIKANRSLHIMCTVPGFCRIYGSSIGYYLKNSTGQPQEMTVAPKTL
XP_027324583.1  330 MFLDNRDLSSQVLHHIKANRSLHIMCTVPSFCRISGSSIGYYLKTSTDQSQEMTAAPKTL
XP_013032636.1  330 MFLDNRDLSSQVLHHIKANRSLHIMCTVPGFCWISGSSIGYYLKNSTDQSQEMTVVPKTL
NP_849172.2     301 MFPEDQALLGWMLSQVQADRALYLMCTVPAFCRLTGMALGHLWRSRTGPQDAELWPPRTL

XP_015150161.2  420 SEIYSYYFKMALSSDWPERQRETLRIEQAVNNSKKVMGSLGRLAFYGLLKRKHVFYEQDM
XP_027324583.1  390 SEIYSYYFKMALSSDWPEKQRETLRIEQAVNNSKKILGSLGRLAFYGLLKRKYVFYEQDM
XP_013032636.1  390 SEIYSYYFKMTLSCDWPEKQRETLRIEQAVNNSKKIMGSLGRLAFYGLLKRKYVFYEQDM
NP_849172.2     361 CELYSWYFRMALSGEGQEKGKASPRIEQVAHGGRKMVGTLGRLAFHGLLKKKYVFYEQDM

XP_015150161.2  480 KAYGIDLSLLHSSLCSRLLLKED-MQSTTAYYFSHLTIQEFLAAIYYYTAAKRAIFDLFT
XP_027324583.1  450 KTYGIDLSLLQSSLCSRLLLKEE-VQSSTAYYFSHLTIQEFLAAIYYYTAAKRAIFDLFT
XP_013032636.1  450 KTYGIDLSLLQSSLCSRLLLKEE-MQSSTAYYFSHLTIQEFLAAIYYYTAAKRAIFDLFT
NP_849172.2     421 KAFGVDLALLQGAPCSCFLQREETLASSVAYCFTHLSLQEFVAAAYYYGASRRAIFDLFT

XP_015150161.2  539 ESGMSWPKLGFLNHFKSAVQRSLQAEDGQLDIFVRFLSGLLSPQVNQLLSGWLLVKDEHN
XP_027324583.1  509 ENGMSWPKLGFLNHFKSAVQRSLQAEDGQLDIFVRFLSGLLSPQVNKLLSGWLLAKDEHN
XP_013032636.1  509 ENGMSWPKLGFLNHFKSAVQRSLQAEDGQLDIFVRFLSGLLSPQVNKLLSGWLLAKDEHN
NP_849172.2     481 ESGVSWPRLGFLTHFRSAAQRAMQAEDGRLDVFLRFLSGLLSPRVNALLAGSLLAQGEHQ

XP_015150161.2  599 SYRSQAISFLQGCLNTNYVISSQTVNTVHCLHEIQHTEIAKAVEEAMKNESLAGMLTPVN
XP_027324583.1  569 SFRSQAISFLQGCLNTDYVISSRTVNTVHCLQEIQHMEIAKSVEEAMKNESLAGMLTPVN
XP_013032636.1  569 SFRSQAISFLQGCLNTDYVISSRTVNTVHCLYEIQHMEIAKSVEEAMKNESLAGMLTPVN
NP_849172.2     541 AYRTQVAELLQGCLRPDAAVCARAINVLHCLHELQHTELARSVEEAMESGALARLTGPAH

XP_015150161.2  659 CSALAYLLQVSDVCVEETNLSNCLTYNICKSLLPQLLFCHSLRLDNNQFKDNVMELLGSM
XP_027324583.1  629 CSVLAYLLQVSDVCVEETNLSNCLTYNVCKSLLPQLLFCHNLRLDNNQFKDNVMELLGSV
XP_013032636.1  629 CSVLAYLLQVSNVCMEETNLSNCLTYNVCKSLLPQLLFCHNLRLDNNQFKDNVMELLGSV
NP_849172.2     601 RAALAYLLQVSDACAQEANLSLSLSQGVLQSLLPQLLYCRKLRLDTNQFQDPVMELLGSV
```

```
XP_015150161.2  719 LSVKDCQIQRLSLAENQICNKGAKALARSLMVNRSLTVLDLRSNSIGPSGAKALADALKK
XP_027324583.1  689 LSVKDCQIQKLSLAENQISNKGAKALARSLMVNRSLMVLDLRSNSIGPSGAKALADALKK
XP_013032636.1  689 LSVKDCQIQKLSLAENQISNKGAKALARSLMVNRSLMVLDLRSNSIGPSGAKALADALKK
NP_849172.2     661 LSGKDCRIQKISLAENQISNKGAKALARSLLVNRSLTSLDLRGNSIGPQGAKALADALKI

XP_015150161.2  779 NQVLLSLSLQHNVIKEEGAAALAEALLTNRRLITLHLQKNSIGAHGARKLAEALAQNCSL
XP_027324583.1  749 NQVLLSLNLQHNVIKEDGATFLAEALLTNHKLTTLHLQKNSIGAQGARKIAEALKRNCSL
XP_013032636.1  749 NQVLLSLNLQHNVIKEDGAAFLAEALLTNHKLTTLHLQKNSIGAQGARKIAEALKRNCSL
NP_849172.2     721 NRTLTSLSLQGNTVRDDGARSMAEALASNRTLSMLHLQKNSIGPMGAQRMADALKQNRSL

XP_015150161.2  839 KELMLSSNSVGDNGSVALAEALKVNHSLQSLDLQSNSISNTGVSALTAALCSNKGLTDLN
XP_027324583.1  809 KELMLSSNSVGDNGSVALAEALKVNHSLQSLDLQSNSISSAGVAALTAALCSNKGLVNLN
XP_013032636.1  809 KELMLSSNSVGDNGSVALAEALKVNHSLQSLDLQSNSISSAGVAALTVALCSNKGLINLN
NP_849172.2     781 KELMFSSNSIGDGGAKALAEALKVNQGLESLDLQSNSISDAGVAALMGALCTNQTLLSLS

XP_015150161.2  899 LRENSISKEGGPAIAHALRTNCTLRKLDLAANLLHDEGGKAIALAMKENRALTSLHLQWN
XP_027324583.1  869 LRENSISKEGGPAIARALRTNNTLRRLDLAANLLYDEGGKAIALAMKENRALTSLHLQWN
XP_013032636.1  869 LRENSISKEGGPAIARALRTNSTLRRLDLAANLLYDEGGKAIALAMKENRALTSLHLQWN
NP_849172.2     841 LRENSISPEGAQAIAHALCANSTLKNLDLTANLLHDQGARAIAVAVRENRTLTSLHLQWN

XP_015150161.2  959 FIQTQAAVALAQALQSNDSLASLDLQENAIGDEGMAALAAALKVNTTLADLHLQVASISA
XP_027324583.1  929 FIQANAATALAQALKSNSSLASLDLQENAIGDEGMAALSAALKVNTTLADLHLQVASIGA
XP_013032636.1  929 FIQANAATALAQALKSNSSLASLDLQENAIGDEGMAALSAALKVNTTLADLHLQVASVGA
NP_849172.2     901 FIQAGAAQALGQALQLNRSLTSLDLQENAIGDDGACAVARALKVNTALTALYLQVASIGA

XP_015150161.2 1019 AGAQALAEALMVNNSLQVLDLRGNSISVAGAKAMANALKVNRSLRWLNLQENSLGMDGAI
XP_027324583.1  989 AGAQALAEALMVNKSLQILDLRGNSIGVAGAKAMANALKVNRSLRRLNLQENSLGMDGAI
XP_013032636.1  989 AGAQALAEALMVNKSLQILDLRGNFIGVAGAKAMANALKVNRSLRRLNLQENSLGMDGAI
NP_849172.2     961 SGAQVLGEALAVNRTLEILDLRGNAIGVAGAKALANALKVNSSLRRLNLQENSLGMDGAI

XP_015150161.2 1079 CIATALRGNHGLTYVNLQGNRIGQSGAKMISDAIRTNSPDCVVVV
XP_027324583.1 1049 CIATALKGNHGLTYVNLQGNRIGQSGAKMISDAIRTNAPDCIVEV
XP_013032636.1 1049 CIATALKGNHGLTYINLQGNRIGQSGAKMISDAIRTNSPDCVVEV
NP_849172.2    1021 CIATALSGNHRLQHINLQGNHIGDSGARMISEAIKTNAPTCTVEM
```

Figure S5: Schematic alignment of NLRC3 protein sequences of chicken, duck goose and human comprising the N-terminal CARD domain, NBD, and the C-terminal LRR.

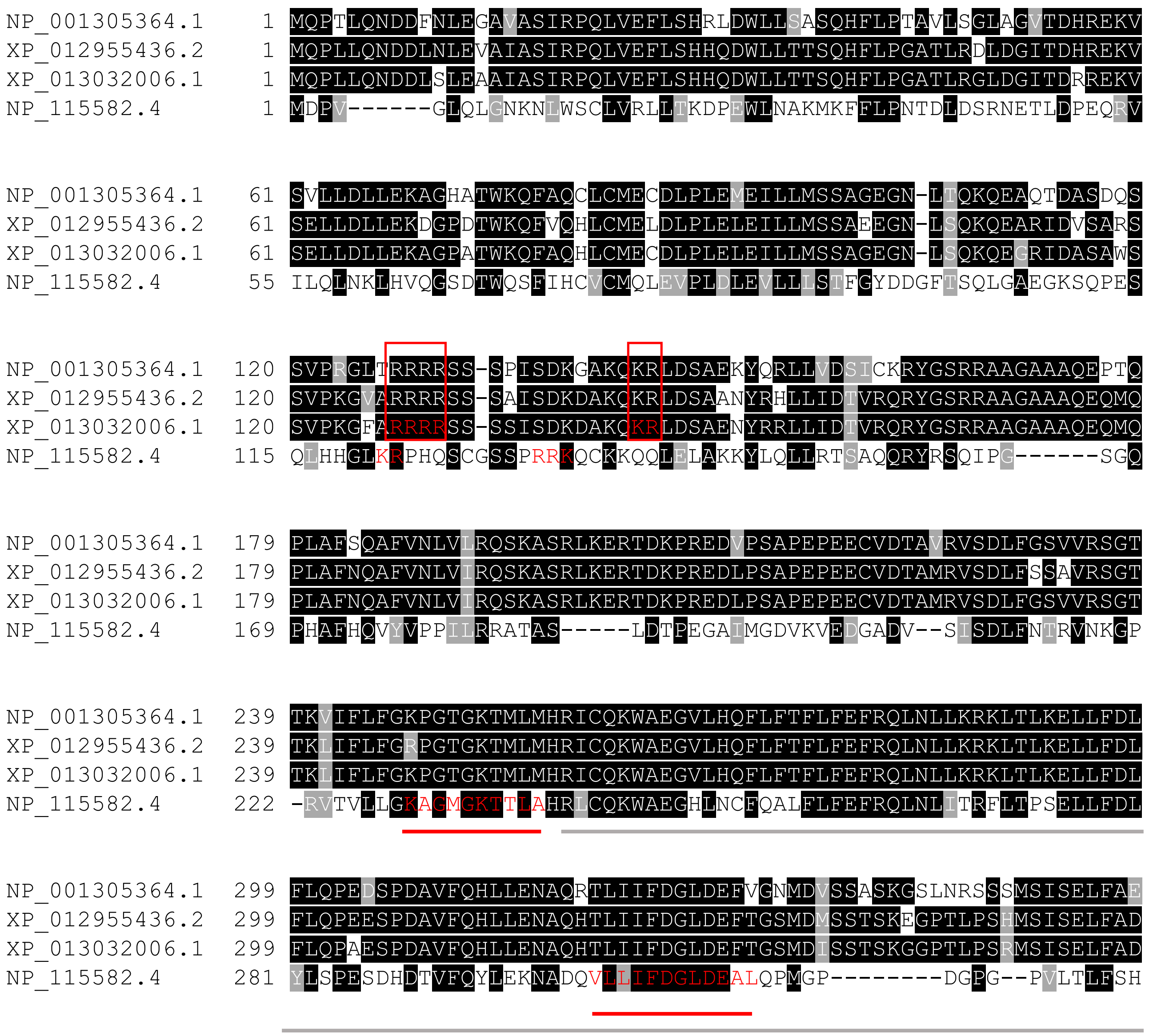
NP_001305364.1 1 MQPTLQNDDFNLEGAVASIRPQLVEFLSHRLDWLLSASQHFLPTAVLSGLAGVTDHREKV
XP_012955436.2 1 MQPLLQNDDLNLEVAIASIRPQLVEFLSHHQDWLLTTSQHFLPGATLRDLDGITDHREKV
XP_013032006.1 1 MQPLLQNDDLSLEAAIASIRPQLVEFLSHHQDWLLTTSQHFLPGATLRGLDGITDRREKV
NP_115582.4 1 MDPV------GLQLGNKNLWSCLVRLLTKDPEWLNAKMKFFLPNTDLDSRNETLDPEQRV
NP_001305364.1 61 SVLLDLLEKAGHATWKQFAQCLCMECDLPLEMEILLMSSAGEGN-LTQKQEAQTDASDQS
XP_012955436.2 61 SELLDLLEKDGPDTWKQFVQHLCMELDLPLELEILLMSSAEEGN-LSQKQEARIDVSARS
XP_013032006.1 61 SELLDLLEKAGPATWKQFAQHLCMECDLPLELEILLMSSAGEGN-LSQKQEGRIDASAWS
NP_115582.4 55 ILQLNKLHVQGSDTWQSFIHCVCMQLEVPLDLEVLLLSTFGYDDGFTSQLGAEGKSQPES
NP_001305364.1 120 SVPRGLTRRRRSS-SPISDKGAKQKRLDSAEKYQRLLVDSICKRYGSRRAAGAAAQEPTQ
XP_012955436.2 120 SVPKGVARRRRSS-SAISDKDAKQKRLDSAANYRHLLIDTVRQRYGSRRAAGAAAQEQMQ
XP_013032006.1 120 SVPKGFARRRRSS-SSISDKDAKQKRLDSAENYRRLLIDTVRQRYGSRRAAGAAAQEQMQ
NP_115582.4 115 QLHHGLKRPHQSCGSSPRRKQCKKQQLELAKKYLQLLRTSAQQRYRSQIPG------SGQ
NP_001305364.1 179 PLAFSQAFVNLVLRQSKASRLKERTDKPREDVPSAPEPEECVDTAVRVSDLFGSVVRSGT
XP_012955436.2 179 PLAFNQAFVNLVIRQSKASRLKERTDKPREDLPSAPEPEECVDTAMRVSDLFSSAVRSGT
XP_013032006.1 179 PLAFNQAFVNLVIRQSKASRLKERTDKPREDLPSAPEPEECVDTAMRVSDLFGSVVRSGT
NP_115582.4 169 PHAFHQVYVPPILRRATAS-----LDTPEGAIMGDVKVEDGADV--SISDLFNTRVNKGP
NP_001305364.1 239 TKVIFLFGKPGTGKTMLMHRICQKWAEGVLHQFLFTFLFEFRQLNLLKRKLTLKELLFDL
XP_012955436.2 239 TKLIFLFGRPGTGKTMLMHRICQKWAEGVLHQFLFTFLFEFRQLNLLKRKLTLKELLFDL
XP_013032006.1 239 TKLIFLFGKPGTGKTMLMHRICQKWAEGVLHQFLFTFLFEFRQLNLLKRKLTLKELLFDL
NP_115582.4 222 -RVTVLLGKAGMGKTTLAHRLCQKWAEGHLNCFQALFLFEFRQLNLITRFLTPSELLFDL
NP_001305364.1 299 FLQPEDSPDAVFQHLLENAQRTLIIFDGLDEFVGNMDVSSASKGSLNRSSSMSISELFAE
XP_012955436.2 299 FLQPEESPDAVFQHLLENAQHTLIIFDGLDEFTGSMDMSSTSKEGPTLPSHMSISELFAD
XP_013032006.1 299 FLQPAESPDAVFQHLLENAQHTLIIFDGLDEFTGSMDISSTSKGGPTLPSRMSISELFAD
NP_115582.4 281 YLSPESDHDTVFQYLEKNADQVLLIFDGLDEALQPMGP--------DGPG--PVLTLFSH

```
NP_001305364.1  359 LCHGNLLPGCTVLVTSRPKRVPDFLINTVDLLAEVWGFDHEKVEEYVSHYFRHHSFKEQA
XP_012955436.2  359 LCHGKLLPGCTVLVTSRPKRLPDFLLNTVDVLAEVWGFDHKKVEEYVSHYFHHHSFKEQA
XP_013032006.1  359 LCHGKLLPGCTVLVTSRPKRLPDLLLNTVDVLAEVWGFDHKKVEEYVSHYFHHHSFKEQA
NP_115582.4     331 LCNGTLLPGCRVMATSRPGKLPACLPAE-AAMVHMLGFDGPRVEEYVNHFFSAQPSREGA

NP_001305364.1  419 IAHLKNNTKLLSMCQIPALCYVVCICLEYLLLKHQ--TSVELPQTMTQFYIKMLLIFINK
XP_012955436.2  419 IAHLKNNTKLLSMCLIPALCYVVCICLEYLLLKHQ--MSVELPQTMTQFYIKMLLIFINK
XP_013032006.1  419 LAHLKNNTKLLSMCLIPALCYVVCICLEYLLLKHQ--MSVELPQTMTQFYIKMLLIFINK
NP_115582.4     390 LVELQTNGRLRSLCAVPALCQVACLCLHHLLPDHAPGQSVALLPNMTQLYMQMVLALSPP

NP_001305364.1  477 QQGEHAGDEEAQLNSNKKAILGLCDLALKGLEAKKLVFYVGDIPEHVKEFASLHGLLTVF
XP_012955436.2  477 QQGEHAVDEETQLNCNKKAILGLCDLALKGLEDKKLVFYVSDIPEDVKEFASLHGLLTVF
XP_013032006.1  477 QQGEHAVDEETQLNCNKKAILGLCDLALKGLEDKKLVFYVSDIPEHVKEFASLHGLLTVF
NP_115582.4     450 ----------GH--LPTSSLLDLGEVALRGLETGKVIFYAKDIAPPLIAFGATHSLLTSF

NP_001305364.1  537 EVKTNSAHPETGYAFVHLSLQEFFAALCLMISKSVDKSHLKRKLSLKSKWTLRNEAKTGF
XP_012955436.2  537 EVKTSGTHPEAGYAFVHLSLQEFFAALCLMINKRVDKSYLKKKFSLKSKWTLKNEAKTEF
XP_013032006.1  537 EVKTSGTHPEAGYAFVHLSLQEFFAALCLMINKRVDKSHLKKKFSLKSKWTLKNEAKTEF
NP_115582.4     498 CVCTGPGHQQTGYAFTHLSLQEFLAALHLMASPKVNKDTLTQYVTLHSRWVQRTKARLGL

NP_001305364.1  597 MESFHIFLSGLSSKECRTFIMLLAEQSEAWVQDKQDAILQSLKKLAATQLTGPKVIELCH
XP_012955436.2  597 IESFHIFLSGLSSKECRTFLMLLAEQNEAWVQDKQDAILQSLKKLAATHLTGPKVIELCH
XP_013032006.1  597 IESFHIFLSGLSSKECRTFLMLLAEQNEAWVQDKQDAILQSLKKLAATHLTGPKVIELCH
NP_115582.4     558 SDHLPTFLAGLASCTCRPFLSHLAQGNEDCVGAKQAAVVQVLKKLATRKLTGPKVVELCH

NP_001305364.1  657 CTFETQGLEVAQHIGSLLNFKYEFKNFRLTPLDMSALVFVINSGQDVTHLDFAGCPLDTD
XP_012955436.2  657 CTFETQDLKVAQHIGSLLNFKYEFKNFRLTPLDMSALVFVINSGQDLTHLDFAGCLMDAG
XP_013032006.1  657 CTFETQDLKVAQHIGSLLNFKYEFKNFRLTPLDMSALVFVINSGQDLTHLDFAGCLMDAG
NP_115582.4     618 CVDETQEPELASLTAQSLPYQLPFHNFPLTCTDLATLTNILEHREAPIHLDFDGCPLEPH
```

```
NP_001305364.1  717 CLEMLASCRNVEHLSFRSRRFGDDFAAALSKGLGEMGSLKKLEVTGGSITAAGLTDMVQA
XP_012955436.2  717 CLEVLASCKNVEHLSFRSRRFGDDFAAALSKSLREMGSLKKLELTGGNITAEGLTNLVQA
XP_013032006.1  717 CLEVLASCKNVEHLSFRSRRFGDDFAAALSKSLREMGSLKKLELTGGNITAEGLTNLVQA
NP_115582.4     678 CPEALVGCGQIENLSFKSRKCGDAFAEALSRSLPTMGRLQMLGLAGSKITARGISHLVKA

NP_001305364.1  777 LSHCLQLEEINLQDNRIQNPDVKTVMELFSRMEKLKKIDLSKNSLSLNAVLLLAKEFIAC
XP_012955436.2  777 SSQCLQLEEINLQDNRIRDLEVKRVLDLFSRMEKLKKVDLSNNNLSLNAVLILAKEVVTC
XP_013032006.1  777 SSQCLQLEEINLQDNRIRDLEVKRVLDLFSRMEKLKKIDLSNNNLSLNAVLILAKEVITC
NP_115582.4     738 LPLCPQLKEVSFRDNQLSDQVVLNIVEVLPHLPRLRKLDLSSNSICVSTLLCLARVAVTC

NP_001305364.1  837 QNAAELHVRKDTVIISFS---GPSGKVPRSLDLKREQNKECVTPTRHLRLCLQARCLSSQ
XP_012955436.2  837 QNATELHVRKDIVIINFS---GTSGKVQRSLDLRWEENKECVIPTRHVKLCLQDRGLSLQ
XP_013032006.1  837 QNATELHVRKDTVIISFS---GTSGKVPRSLDLRWEENKECVIPTRHMKLCLRDCSLSLQ
NP_115582.4     798 PTVRMLQAREADLIFLLSPPTETTAELQRAPDLQESDGQRKGAQSRSLTLRLQKCQLQVH

NP_001305364.1  894 HAKEIVSILQSCPHLSEVDLSDNKLGDEGCSFLLENLSWISISKQLNLSHNLLSVTGIYS
XP_012955436.2  894 HAKEIVSILQSCPHLSEVDLSGNKLGDEGCSCLLESLPWISISKQLDLSHNLLSINGIYS
XP_013032006.1  894 HAKEIVSILQSCPHLSEVDLSGNKLGDEGCSCLLESLPWISISKQLDLSHNLLSINGIYS
NP_115582.4     858 DAEALIALLQEGPHLEEVDLSGNQLEDEGCRLMAEAASQLHIARKLDLSNNGLSVAGVHC

NP_001305364.1  954 LLKAVNTCQRVVEVEVSLYHNTAVLRFTEDGEFASPPASREEPLYPTDDQWDDKENQTPV
XP_012955436.2  954 LLKSVNTCQKTMEVEVSLCHKTAVLRFTEDKEFASP--HREEPLYPTDDQQVGEEKQTPT
XP_013032006.1  954 LLKSVNTCQKTMEVEASLCHKTAVLRFTEDREFASPP-HREEPLYPTDDQQVGEEKQTPT
NP_115582.4     918 VLRAVSACWTLAELHISLQHKTVIFMFAQEPEEQKGPQERAAF---LDSLMLQMPSELPL

NP_001305364.1 1014 PSKKIRLTYCRFQASDLEKLCAVLKECGSIS--ELDLSNNYLGDEGLAQLLQFLPNLKTL
XP_012955436.2 1012 LSKKIRLTDCYFQASDLEKLCAVLKECSSIS--ELDLSNNSLGDEGLSRLFQFLPNLKML
XP_013032006.1 1013 LSKKIRLTDCHFQASDLEKLCAVLQECSSIS--ELDLSNNSLGDEGLSRLFQFLPNLKML
NP_115582.4     975 SSRRMRLTHCGLQEKHLEQLCKALGGSCHLGHLHLDFSGNALGDEGAARLAQLLPGLGAL
```

```
NP_001305364.1 1072 RSLKLNNNHLSLSSVFCLAQSLCTLEHLETMDLSLGRMQVVHLTFGDRIRLR-RTSRWRR
XP_012955436.2 1070 RSLKLDNNRISLNSVFCLAQSLSTLERIKTMNLSLGHMQVVHLTFWERIRDR-STGRLRR
XP_013032006.1 1071 RSLKLDNNRISLNSVFCLAQSLSTLEHIKTMNLSLGHMQVVHLSFWERIRDR-STGRLRR
NP_115582.4    1035 QSLNLSENGLSLDAVLGLVRCFSTLQWLFRLDISFESQHIL-LRGDKTSRDMWATGSL-P

NP_001305364.1 1131 SFLVHPKHVT-----NGQCFRLRNCTVGPEDVTRLCQMLTQCTQLTEIDLCGNALNDQSI
XP_012955436.2 1129 NFLVHPKHIE-----NGQCFRLRDCTMGPEDVTRLCQILVHCTQLTEINLSGNPLSDQSI
XP_013032006.1 1130 NFLVHPKHIA-----NGRCFRLRDCTMGPEDMTRLCQILAHCTQLTEIDLSGNSLSDQSI
NP_115582.4    1093 DFPAAAKFLGFRQRCIPRSLCLSECPLEPPSLTRLCATLKDCPGPLELQLSCEFLSDQSL

NP_001305364.1 1186 ERLLSFLPHFCQLTLLSIRNNTFSPCCAVLFANSINLCERIRRVEVRSSQNAFLHLRTST
XP_012955436.2 1184 ERLLSFLPYLCHLTLLSIKNSTFSPRCAILFTNSIGLCERIRKVEVRSNQNAFLHLGTST
XP_013032006.1 1185 ERLLSFLPYLCHLTLLSIKNSTFSPHCTILFTNSIGLSERIRKVEVRSNQNAFLHLGTSA
NP_115582.4    1153 ETLLDCLPQLPQLSLLQLSQTGLSPKSPFLLANTLSLCPRVKKVDLRSLHHATLHFRSNE

NP_001305364.1 1246 QSQKTSC-RLTDCAIGQRQIEKLCRVLEQHGCLAELDLSRNQLGDEVLRFLLDHLHRVPV
XP_012955436.2 1244 HSQKTSC-SLTDCAISQGQIEELCGVLEQHGRLAEVDLSRNQLGDEGLRFLLDHLHRVHV
XP_013032006.1 1245 RSQKTSC-RLTDCAISQGQIEELCRVLEQHGRLAEVDLSRNQLGDEGLRFLLDHLHRVHV
NP_115582.4    1213 EEEGVCCGRFTGCSLSQEHVESLCWLLSKCKDLSQVDLSANLLGDSGLRCLLECLPQVPI

NP_001305364.1 1305 TCSLNLSHNRISQRGVLHLINTFTTSGNTTEVQVSLCSKATLIIKMTSRDDPRKILRLTE
XP_012955436.2 1303 TCSLNLSHNRISQDGVLHLINAFATSGNVTEVLVSLCSKATLLMELTSRDDPRKILSLTE
XP_013032006.1 1304 TCSLNLSHNRISQDGVLHLINAFATSRNITEVVVSLCSKATLLMKLTSRDDPRKILSLTE
NP_115582.4    1273 SGLLDLSHNSISQESALYLLETLPSCPRVREASVNLGSEQSFRIHFSREDQAGKTLRLSE

NP_001305364.1 1365 CSFQPEHLEKLCLVLENCTNLTECISSNNNVTVDAAGGLLRSLSKTPGPLKISIEEPWVC
XP_012955436.2 1363 CNFQAEHLEKLWLVLEKCTSLTEYISSSNNLTVHAAERLLHSLRKTSGPLKISIKEPWVC
XP_013032006.1 1364 CNFQPEHLEKLWLALEKCTSLTEYISSNNNMTVHAAERLLHSLRKTPGPLKISIKEPWVC
NP_115582.4    1333 CSFRPEHVSRLATGLSKSLQLTELTLTQCCLGQKQLAILLSLVGRPAGLFSLRVQEPWAD
```

```
NP_001305364.1 1425 KLSVTSLLELAVQAHGNITAIMICKEKNLFQLGVRLPHCLEKVGSVVSRLNLNEPEIKQA
XP_012955436.2 1423 KKSVMNLLELAVQACGNITAITICKDKSLFQLGVSFPCCLEKVESVVSRLNLHEPEIKRA
XP_013032006.1 1424 KKSVTNLLELAVQACGNITAIMICKDKSLFQLGVRFPCCLEKVESVVSRSNLHEPEIKRA
NP_115582.4    1393 RARVLSLLEVCAQASGSVTEISISETQQQLCVQLEFPRQEENPEAVALRLAHCDLGAHHS

NP_001305364.1 1485 CFYQRVHDKCTQLQELRWSHVELHDD----TEMLVSILLPLPDLKKFELTSCSFTPTGID
XP_012955436.2 1483 CFYQRVLEKCIQLQELRWSHVELHDDD---TKILVTVLLPLPKLKKFELTSCSIMPTGID
XP_013032006.1 1484 CFYQRVLEKCIQLQELRWSHVELHDDD---TKILVTVLLPLPKLKKFELTSCSIMPTGID
NP_115582.4    1453 LLVGQLMETCARLQQLSLSQVNLCEDDDASSLLLQSLLLSLSELKTFRLTSSCVSTEGLA

NP_001305364.1 1541 CLITGLQRCQAIEELN----------------------------LGHMKLGDAAIPKLVF
XP_012955436.2 1540 YLITGLQKCQAIEELS----------------------------LGHMKLSDAAIPMLVL
XP_013032006.1 1541 YLITGLQKCQAIEELN----------------------------LGHMKLSDAAIPKLVL
NP_115582.4    1513 HLASGLGHCHHLEELDLSNNQFDEEGTKALMRALEGKWMLKRLDLSHLLLNSSTLALLTH

NP_001305364.1 1573 GLCEMPSLKRLILNHNSIGDDGCSRLAEALSSMHCMEEINLGHNKIGDLGLINIAAVLLE
XP_012955436.2 1572 GLCKMPSLKRLILNHNSIGNDGCSRLAEALRNMHCMEEINLAHNKIGDPGLINIATVLLE
XP_013032006.1 1573 GLCKMPSLKRLILNHNSIGDDGCSRLAEALRNMHCMEEINLAHNKIGDPGLINIATALLE
NP_115582.4    1573 RLSQMTCLQSLRLNRNSIGDVGCCHLSEALRAATSLEELDLSHNQIGDAGVQHLATILPG

NP_001305364.1 1633 MQNLKRIDLSGNCPSPAGGEKLMEALANCKHLEELILSRNDFGDGTAVKLALCLPHMNRL
XP_012955436.2 1632 MQNLKRINLSGNSPSPTGGEKLMEALACCKHIEELLLSRNVFGDGVAVKLALCLPHVSSL
XP_013032006.1 1633 MQNLKRINLSGNSPSPAGGEKLMEALAYCKHIEELLLSRNVFGDGTAVKLALCLPHVSSL
NP_115582.4    1633 LPELRKIDLSGNSISSAGGVQLAESLVLCRRLEELMLGCNALGDPTALGLAQELPQ--HL

NP_001305364.1 1693 KILHLQHNNIGPAGGTELARALMACELLEEISLSENNLGEGGIRALSEGLPRFEHLRKIE
XP_012955436.2 1692 KILHLQKNNIGPTGGTELARALVACRLLEEISLSENDLGEGSIHALSEGLPRLEHLRKID
XP_013032006.1 1693 KILHLQKNNIGPTGGTELARALVVCGLLEEISLSENDLGEGSIHALSEGLPHLEHLRKID
NP_115582.4    1691 RVLHLPFSHLGPGGALSLAQALDGSPHLEEISLAENNLAGGVLRFCME-LP---LLRQID
```

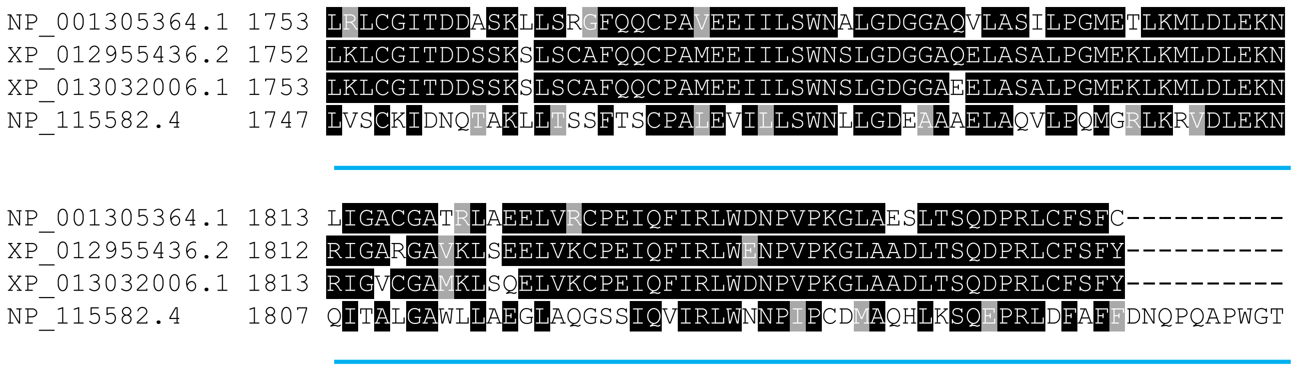

Figure S6: Schematic alignment of NLRC5 protein sequences of chicken, duck goose and human comprising the N-terminal CARD domain, NBD, and the C-terminal LRR. NBD and LRR domains were underscored in grey and blue respectively. Putative bipartite NLS and Walker A/B motifs were box highlighted and underscored in red respectively. The residues conforming to bipartite NLS and Walker A/B motif consensus were highlighted in red for human.

```
NP_989894.1       1 MAAFSEMGVMPEIAQAVEEMDWLLPTDIQAESIPLILGGGDVLMAAETGSGKTGAFSIPV
XP_027311055.1    1 MAAFSEMGVMPEIAQAVEEMDWLLPTDIQAESIPLILGGGDVLMAAETGSGKTGAFSIPV
XP_013028107.1    1 MAAFSEMGVMPEIAQAVEEMDWLLPTDIQAESIPLILGGGDVLMAAETGSGKTGAFSIPV
NP_004930.1       1 MAAFSEMGVMPEIAQAVEEMDWLLPTDIQAESIPLILGGGDVLMAAETGSGKTGAFSIPV

NP_989894.1      61 IQIVYETLKDQMEGKKGKATIKTGGAVLNKWQMNPYDRGSAFAIGSDGLCCQSREVKEWH
XP_027311055.1   61 IQIVYETLKDQMEGKKGKATIKTGGAVLNKWQMNPYDRGSAFAIGSDGLCCQSREVKEWH
XP_013028107.1   61 IQIVYETLKDQMEGKKGKATIKTGGAVLNKWQMNPYDRGSAFAIGSDGLCCQSREVKEWH
NP_004930.1      61 IQIVYETLKDQQEGKKGKTTIKTGASVLNKWQMNPYDRGSAFAIGSDGLCCQSREVKEWH

NP_989894.1     121 GCRATRGVTKGKYYYEVSCHDQGLCRVGWSTMQASLDLGTDKFGFGFGGTGKKSHNKQFD
XP_027311055.1  121 GCRATRGVTKGKYYYEVSCHDQGLCRVGWSTMQASLDLGTDKFGFGFGGTGKKSHNKQFD
XP_013028107.1  121 GCRATRGVTKGKYYYEVSCHDQGLCRVGWSTMQASLDLGTDKFGFGFGGTGKKSHNKQFD
NP_004930.1     121 GCRATKGLMKGKHYYEVSCHDQGLCRVGWSTMQASLDLGTDKFGFGFGGTGKKSHNKQFD

NP_989894.1     181 SYGEEFTMHDTIGCYLDIDKGQIKFSKNGKDLGLAFEFPPHIRNQALFAACVLKNAELKF
XP_027311055.1  181 SYGEEFTMHDTIGCYLDLDKGQIKFSKNGKDLGLAFEIPPHIRNQALFAACVLKNAELKF
XP_013028107.1  181 SYGEEFTMHDTIGCYLDIDKGQIKFSKNGKDLGLAFEIPPHIRNQALFAACVLKNAELKF
NP_004930.1     181 NYGEEFTMHDTIGCYLDIDKGHVKFSKNGKDLGLAFEIPPHMKNQALFPACVLKNAELKF

NP_989894.1     241 NFGEEDFKFPPKDGYIGLCKAPDGNVVKSQHTGNAQVVQTQNLPNAPKALIVEPSRELAE
XP_027311055.1  241 NFGEEDFKFPPKDGYIGLCKAPDGSVVKSQHAGNAQVVQTQNLPNAPKALIVEPSRELAE
XP_013028107.1  241 NFGEEDFKFPPKDGYIGLCKAPDGSVVKSQHAGNAQVVQTQNLPNAPKALIVEPSRELAE
NP_004930.1     241 NFGEEEFKFPPKDGFVALSKAPDGYIVKSQHSGNAQVTQTKFLPNAPKALIVEPSRELAE

NP_989894.1     301 QTLNNVKQFKKYIDNPKLRELLIIGGVAARDQLSVLEQGVDIVVGTPGRLDDLVSTGKLN
XP_027311055.1  301 QTLNNVKQFKKYVDNPKLRELLIIGGVAARDQLSVLEQGVDIVVGTPGRLDDLVSTGKLN
XP_013028107.1  301 QTLNNVKQFKKYVDNPKLRELLIIGGVAARDQLSVLEQGVDIVVGTPGRLDDLVSTGKLN
NP_004930.1     301 QTLNNIKQFKKYIDNPKLRELLIIGGVAARDQLSVLENGVDIVVGTPGRLDDLVSTGKLN
```

```
NP_989894.1      361 LSQVRFLVIDEADGLLLQGYSDFINRIHSQIPQITSDGKRLQVIVCSATLHSFDVKKLSE
XP_027311055.1   361 LSQVRFLVIDEADGLLLQGYSDFINRIHSQIPQITSDGKRLQVIVCSATLHSFDVKKLSE
XP_013028107.1   361 LSQVRFLVIDEADGLLLQGYSDFINRIHSQIPQITSDGKRLQVIVCSATLHSFDVKKLSE
NP_004930.1      361 LSQVRFLVIDEADGLLSQGYSDFINRMHNQIPQVTSDGKRLQVIVCSATLHSFDVKKLSE

NP_989894.1      421 KIMHFPTWVDLKGEDSVPETVHHVVVIVNPKTDKLWERLGKNHIRTDEVHAKDNTLPGAN
XP_027311055.1   421 KIMHFPTWVDLKGEDSVPETVHHVVVPVNPKADKLWERLGKNHIRTDEVHAKDNTRPGAN
XP_013028107.1   421 KIMHFPTWVDLKGEDSVPETVHHVVVPVNPKADKLWERLGKNHIRTDEVHAKDNTRPGAN
NP_004930.1      421 KIMHFPTWVDLKGEDSVPDTVHHVVVPVNPKTDRLWERLGKSHIRTDDVHAKDNTRPGAN

NP_989894.1      481 TPEMWSEAIKILKGEYTVRAIKEHKMDQAIIFCRTKIDCDNMEQYFIQQGGGPDRKGHQF
XP_027311055.1   481 TPEMWSEAIKILKGEYTVRAIKEHKMDQAIIFCRTKIDCDNMEQYFIQQGGGPDRKGHQF
XP_013028107.1   481 TPEMWSEAIKILKGEYTVRAIKEHKMDQAIIFCRTKIDCDNMEQYFIQQGGGPDRKGHQF
NP_004930.1      481 SPEMWSEAIKILKGEYAVRAIKEHKMDQAIIFCRTKIDCDNLEQYFIQQGGGPDKKGHQF

NP_989894.1      541 SCVCLHGDRKPQERKQNLERFKRGDVRFLICTDVAARGIDIHGVPYVINVTLPDEKQNYV
XP_027311055.1   541 SCVCLHGDRKPQERKQNLERFKRGDVRFLICTDVAARGIDIHGVPYVINVTLPDEKQNYV
XP_013028107.1   541 SCVCLHGDRKPQERKQNLERFKRGDVRFLICTDVAARGIDIHGVPYVINVTLPDEKQNYV
NP_004930.1      541 SCVCLHGDRKPHERKQNLERFKKGDVRFLICTDVAARGIDIHGVPYVINVTLPDEKQNYV

NP_989894.1      601 HRIGRVGRAERMGLAISLVAKEKEKVWYHVCSSRGKGCYNTRLKEEGGCTIWYNEMQLLG
XP_027311055.1   601 HRIGRVGRAERMGLAISLVAKEKEKVWYHVCSSRGKGCYNTRLKEEGGCTIWYNEMQLLG
XP_013028107.1   601 HRIGRVGRAERMGLAISLVAKEKEKVWYHVCSSRGKGCYNTRLKEEGGCTIWYNEMQLLG
NP_004930.1      601 HRIGRVGRAERMGLAISLVATEKEKVWYHVCSSRGKGCYNTRLKEDGGCTIWYNEMQLLS

NP_989894.1      661 EIEEHLNCTISQVEPDIKVPVDDFDGKVTYGQKRALGGGLYKGHVDILAPTVQELAALEK
XP_027311055.1   661 EIEEHLNCTIAQIEPDIKVPVDDFDGKVTYGQKRALGGGLYKGHVDILAPTVQELAALEK
XP_013028107.1   661 EIEEHLNCTIAQIEPDIKVPVDDFDGKVTYGQKRALGGGLYKGHVDILAPTVQELAALEK
NP_004930.1      661 EIEEHLNCTISQVEPDIKVPVDEFDGKVTYGQKRAAGGGSYKGHVDILAPTVQELAALEK

NP_989894.1      721 EAQTSFLHLGYLPNQLFRTF
XP_027311055.1   721 EAQTSFLHLGYLPNQLFRTF
XP_013028107.1   721 EAQTSFLHLGYLPNQLFRTF
NP_004930.1      721 EAQTSFLHLGYLPNQLFRTF
```

Figure S7: Schematic alignment of DDX1 protein sequences of chicken, duck, goose and human. Depicted are the DEAD box region, and HelicC domain in black, and red underlining respectively. The DEAD box is boxed in blue.

```
XP_001232052.1     1 MPEATRICAGS-GAESE-------------------------------------------
XP_027316557.1     1 MPEATRICAGS-GAEAG-------------------------------------------
XP_013030257.1     1 LEVSRLVCEPSLRAASV-------------------------------------------
NP_004719.2        1 MPGKLRSDAGL-ESDTAMKKGETLRKQTEEKEKKEKPKSDKTEEIAEEEETVFPKAKQVK

XP_001232052.1    17 --------------------PESPMESEAAPESSRRRRKKEKKEKKSKRHDKARKRKPQT
XP_027316557.1    17 --------------------SDSPMESEAATANGRRRRKKEKKEKKAKRRDKAPKRKSQT
XP_013030257.1    18 --------------------PRGSGDARVGTHAS-VVPQKEKKEKKAKRRDKARKRKSQT
NP_004719.2       60 KKAEPSEVDMNSPKSKKAKKKEEPSQNDISPKTKSLRKKKEPIEKK----VVSSKTKKVT

XP_001232052.1    57 DESEQSEEECSSPKLKKAKTGGKQNGHVREESPENARLSSVSAKSTHKKRHSNSSETSSG
XP_027316557.1    57 DESEQSEDELDPPKVKKAKSREKQNGDARGESPENARLSSLTLKAARKKQPSNSSETSSG
XP_013030257.1    57 DESEQSEDELDSPKVKKAKSREKQNGDAGGDSPENARLSSLTLKSAHKKRPRNASETSSG
NP_004719.2      116 KNEEPSEEEIDAPKPKKMKKEKEMNGETREKSPK------LKNGFPHPEPDCNPSEAASE

XP_001232052.1   117 DGDSDQEQEMTEEQKEGAFSNFPISKGTIQLLQARGVTYLFPVQVKTFNPVYSGKDVIAQ
XP_027316557.1   117 DCDSDQEQEMTEEQKEGAFSNFPISKGTVELLQARGVTYLFPVQVKTFHPVFSGKDVIAQ
XP_013030257.1   117 DCDSDQEQEMTEEQKEGAFSNFPISKVTVELLQARGVTYLFPVQVKTFHPVFSGKDVIAQ
NP_004719.2      170 ESNSEIEQEIPVEQKEGAFSNFPISEETIKLLKGRGVTFLFPIQAKTFHHVYSGKDLIAQ

XP_001232052.1   177 ARTGTGKTFSFAIPLIEKLQADSQERRRGRSPKVLVLAPTRELANQVAKDFKDITRKLTV
XP_027316557.1   177 ARTGTGKTFSFAIPLIEKLQADSQEKRRGRAPKVLVLAPTRELANQVAKDFKDITRKLTV
XP_013030257.1   177 ARTGTGKTFSFAIPLIEKLQADSQERKRGRSPKVLVLAPTRELANQVAKDFKDITRKLTV
NP_004719.2      230 ARTGTGKTFSFAIPLIEKLHGELQDRKRGRAPQVLVLAPTRELANQVSKDFSDITKKLSV

XP_001232052.1   237 ACFYGGTPYNGQIDLIRSGIDILVGTPGRIKDHLQNGKLDLTKVKHVVLDEVDQMLDMGF
XP_027316557.1   237 ACFYGGTPYNGQIDLIRSGIDILVGTPGRIKDHLQNGKLDLSKVKHVVLDEVDQMLDMGF
XP_013030257.1   237 ACFYGGTPYNGQIDLIRSGIDILVGTPGRIKDHLQNGKLDLSKVKHVVLDEVDQMLDMGF
NP_004719.2      290 ACFYGGTPYGGQFERMRNGIDILVGTPGRIKDHIQNGKLDLTKLKHVVLDEVDQMLDMGF
```

```
XP_001232052.1  297  AEQVEDILRVAYKKDSEDNPQTLLFSATCPNWVYDVAKKYMKSKYEQVDLIGRKTQKAAT
XP_027316557.1  297  AEQVEDILRVAYKKDSEDNPQTLLFSATCPHWVYDVAKKYMKSKYEQIDLIGKRTQKAAT
XP_013030257.1  297  AEQVEDILRVAYKKDSEDNPQTLLFSATCPHWVYDVAKKYMKSKYEQIDLIGKRTQKAAT
NP_004719.2     350  ADQVEEILSVAYKKDSEDNPQTLLFSATCPHWVFNVAKKYMKSTYEQVDLIGKKTQKTAI

XP_001232052.1  357  TVEHLAIECHWSQRAAVIGDVIQVYSGSYGRTIVFCETKKDANELALNASIKQDCQSLHG
XP_027316557.1  357  TVEHLAIACHWSQRAAVIGDVIQVYSGSHGRTIVFCETKKDANELALNASIKQDCQSLHG
XP_013030257.1  357  TVEHLAIACHWSQRAAVIGDVIQVYSGSLGRTIVFCETKKDANELALNASIKQDCQSLHG
NP_004719.2     410  TVEHLAIKCHWTQRAAVIGDVIRVYSGHQGRTIIFCETKKEAQELSQNSAIKQDAQSLHG

XP_001232052.1  417  DIPQKQREITLKGFRNGAFKVLVATNVAARGLDIPEVDLVVQSSPPKDVESYIHRSGRTG
XP_027316557.1  417  DIPQKQREITLKGFRNGAFKVLVATNVAARGLDIPEVDLVVQSSPPKDVESYIHRSGRTG
XP_013030257.1  417  DIPQKQREITLKGFRNGAFKVLVATNVAARGLDIPEVDLVVQSSPPKDVESYIHRSGRTG
NP_004719.2     470  DIPQKQREITLKGFRNGSFGVLVATNVAARGLDIPEVDLVIQSSPPKDVESYIHRSGRTG

XP_001232052.1  477  RAGRTGICICFYQRKEENQLRYVEVKAGITFKRVGVPTATDIIKASSKDAIRCLDSVPQT
XP_027316557.1  477  RAGRTGICICFYQRKEENQLRYVEQKAGISFKRVGVPTATDIIKASSKDAIRCLDSVPQS
XP_013030257.1  477  RAGRTGICICFYQRKEENQLRYVEQKAGIAFKRVGVPTATDIIKASSKDAIRCLDSVPQS
NP_004719.2     530  RAGRTGVCICFYQHKEEYQLVQVEQKAGIKFKRIGVPSATEIIKASSKDAIRLLDSVPPT

XP_001232052.1  537  AIEYFKESAQLLIEEKGPVNALAAALAHISGATSIEQRSL----LNSDVGFVTMVLRCSE
XP_027316557.1  537  AIDYFKESAQLLIKEKGPVNALAAALAHISGATSIEQRSL----LNSDAGFVTMILRCSE
XP_013030257.1  537  AIDYFKESAQLLIQEKGPVNALAAALAHISGATSIEQRSLPXXXLNSDAGFVTMILRCSE
NP_004719.2     590  AISHFKQSAEKLIEEKGAVEALAAALAHISGATSVDQRSL----INSNVGFVTMILQCSI

XP_001232052.1  593  EINNMSYVWRRLREQLGGDVDRKVNRMRFIKGRMGVCFDIPVADQKDIEERWEDSKQCSL
XP_027316557.1  593  EINNMSYAWRKLREQLGDDVDRKVNRMCFIKGRMGVCFDIPVADQKEIEGKWEDSKQCRL
XP_013030257.1  597  EINNMSYAWRRLREQLGDDVDRKVNRMCFIKGRMGVCFDIPVADQKEIEGKWEDSKQCRL
NP_004719.2     646  EMPNISYAWKELKEQLGEEIDSKVKGMVFLKGKLGVCFDVPTASVTEIQEKWHDSRRWQL
```

```
XP_001232052.1  653 CVANELPELEESQRGGGGGGGGGGGN-----NRS----------FSGSRNG-RRG-----
XP_027316557.1  653 CVANELPELEESQRGGGG------GN-----GRS----------FSGSRSGGRRG-----
XP_013030257.1  657 CVANELPELEETQRGGG--------N-----GRS----------FSSSRSG-RRG-----
NP_004719.2     706 SVATEQPELEGPREGYGG------FRGQREGSRGFRGQRDGNRRFRGQREG-SRGPRGQR

XP_001232052.1  692 ---GSGRNRFRSRGQKRSYDRAFDR
XP_027316557.1  687 ---GSGRNRFRGRGQKRSYDRAFDR
XP_013030257.1  688 ---GSGRNRFRSRGQKRSYDRAFDR
NP_004719.2     759 SGGGNKSNRSQNKGQKRSFSKAFGQ
```

Figure S8: Schematic alignment of DDX21 protein sequences of chicken, duck, goose and human. Depicted are DEAD box region, GUCT and HelicC domain in black, green and red underlining respectively. The DEAD box is boxed in blue.

```
XP_015147310.1     1 MSYEQRRDWGRGRGRGGDGGS---------------SAASSGGGGH----GGRGGGRGRHP
XP_027320349.1     1 MSSEQRRGWARGGDGGPAASS---------------SSCSSSAGGHGGRGGGGGGGRGRHP
XP_013048508.1     1 ------------------------------------------------------------
NP_065916.2        1 MSYDYHQNWGRDGGP--RSSGGGYGGGPAGGHGGNRGSGGGGGGG----GGGRGGRGRHP

XP_015147310.1    43 SHLKGREIGLWYARKQGQKSKETDRQQRAVVRMDERREEQIVQLLNAVQPRAEKEQEA-M
XP_027320349.1    47 SHLKGREIGLWYARKQGQRSKETDRQQRAVVRMDERREEQIAQLLTAVQSRSEKEPDA-M
XP_013048508.1     1 --------------------------------MDERREEQIAQLLTAVQSRSEKEPDA-M
NP_065916.2       55 GHLKGREIGMWYAKKQGQKNKEAERQERAVVHMDERREEQIVQLLNSVQAKNDKESEAQI

XP_015147310.1   102 SWWSGDEEGHVPEQPPKVKPGAEKAEKAPVKR-----------RPILQKTFLDQDVEYLFE
XP_027320349.1   106 SWWSGDEDGNTPEQPAKVKPEVE---KAPIKQ-----------RPVLEKTFLDRDVEYLFE
XP_013048508.1    28 SWWSGDEDGNTPEQPAKVKPEAE---KAPIKQ-----------RPVLEKTFLDRDVEYLFE
NP_065916.2      115 SWFAPEDHGYGTEVSTKNTPCSEN--KLDIQEKKLINQEKKMFRIRNRSYIDRDSEYLLQ

XP_015147310.1   152 KNDQDADLDEQLKEDLRKKKSDPRYIEMQRFREKLPSYGMRQELVNLINNNRVTVISGET
XP_027320349.1   153 KNQQDTDLDEQLKEDLRKKKSDPRYIEMQRFREKLPSYGMRQELVNLINNNRVTVISGET
XP_013048508.1    75 KNQQDTDLDEQLKEDLRKKKSDPRYIEMQRFREKLPSYGMRQELVNLINNNRVTVISGET
NP_065916.2      173 ENEPDGTLDQKLLEDLQKKKNDLRYIEMQHFREKLPSYGMQKELVNLIDNHQVTVISGET

XP_015147310.1   212 GCGKTTQVTQFILDDYIERGKGSTCRIVCTQPRRISAISVAERVAAERAEACGNGKSTGY
XP_027320349.1   213 GCGKTTQVTQFILDDYIERGKGSTCRIVCTQPRRISAISVAERVAAERAEACGNGKSTGY
XP_013048508.1   135 GCGKTTQVTQFILDDYIERGKGSTCRIVCTQPRRISAISVAERVAAERAEACGNGKSTGY
NP_065916.2      233 GCGKTTQVTQFILDNYIERGKGSACRIVCTQPRRISAISVAERVAAERAESCGSGNSTGY

XP_015147310.1   272 QIRLQSRLPRKQGSILYCTTGIVLQWLQSDKHLSSISHVVLDEIHERNLQSDVLMSIIKD
XP_027320349.1   273 QIRLQSRLPRKQGSILYCTTGIVLQWLQSDKNLSSISHVVLDEIHERNLQSDVLMSIIKD
XP_013048508.1   195 QIRLQSRLPRKQGSILYCTTGIVLQWLQSDKHLSSISHVVLDEIHERNLQSDVLMSIIKD
NP_065916.2      293 QIRLQSRLPRKQGSILYCTTGIILQWLQSDPYLSSVSHIVLDEIHERNLQSDVLMTVVKD
```

```
XP_015147310.1  332 LLNVRLDLKVILMSATLNAEKFSEYFDNCPMIHIPGFTFPVVEYLLEDVIEKLRYTPENT
XP_027320349.1  333 LLNVRLDLKVILMSATLNAEKFSEYFDNCPMIHIPGFTFPVVEYLLEDVIEKLRYMPENT
XP_013048508.1  255 LLNVRLDLKVILMSATLNAEKFSEYFDNCPMVHIPGFTFPVVEYLLEDVIEKLRYMPENT
NP_065916.2     353 LLNFRSDLKVILMSATLNAEKFSEYFGNCPMIHIPGFTFPVVEYLLEDVIEKIRYVPEQK

XP_015147310.1  392 DRRPRWKKGFMQGHISRPEKEEKEEIYRERWPEYLRQLRGRYSAGTIDALEMMDDDKVDL
XP_027320349.1  393 DRRPRWKKGFMQGHISRPEKEEKEEIYRERWPEYLRQLRGRYSASTIDALEMMDDDKVDL
XP_013048508.1  315 DRRPRWKKGFMQGHISRPEKEEKEEIYRERWPEYLRQLRGRYSASTIDALEMMDDDKVDL
NP_065916.2     413 EHRSQFKRGFMQGHVNRQEKEEKEAIYKERWPDYVRELRRRYSASTVDVIEMMEDDKVDL

XP_015147310.1  452 DLIAALIRHIVLEEEDGAILVFLPGWDNISTLHDLLMSQVMFKSDRFIIIPLHSLMPTVN
XP_027320349.1  453 DLIAALIRHIVLEEEDGAILVFLPGWDNISTLHDLLMSQVMFKSDRFIIIPLHSLMPTVN
XP_013048508.1  375 DLIAALIRHIVLEEEDGAILVFLPGWDNISTLHDLLMSQVMFKSDRFIIIPLHSLMPTVN
NP_065916.2     473 NLIVALIRYIVLEEEDGAILVFLPGWDNISTLHDLLMSQVMFKSDKFLIIPLHSLMPTVN

XP_015147310.1  512 QTQVFKKTPPGVRKIVIATNIAETSITIDDVVFVIDGGKIKETHFDTQNNISTMAAEWVS
XP_027320349.1  513 QTQVFKKTPPGVRKIVIATNIAETSITIDDVVFVIDGGKIKETHFDTQNNISTMAAEWVS
XP_013048508.1  435 QTQVFKKTPPGVRKIVIATNIAETSITIDDVVFVIDGGKIKETHFDTQNNISTMAAEWVS
NP_065916.2     533 QTQVFKRTPPGVRKIVIATNIAETSITIDDVVYVIDGGKIKETHFDTQNNISTMSAEWVS

XP_015147310.1  572 KANAKQRKGRAGRVQPGHCYHLYNGLRASLLDDYQLPEILRTPLEELCLQIKILKLGGIA
XP_027320349.1  573 KANAKQRKGRAGRVQPGHCYHLYNGLRASLLDDYQLPEILRTPLEELCLQIKILKLGGIA
XP_013048508.1  495 KANAKQRKGRAGRVQPGHCYHLYNGLRASLLDDYQLPEILRTPLEELCLQIKILKLGGIA
NP_065916.2     593 KANAKQRKGRAGRVQPGHCYHLYNGLRASLLDDYQLPEILRTPLEELCLQIKILRLGGIA

XP_015147310.1  632 YFLSKLMDPPSRDAVMLAINHLMELNALDRQEELTPLGVHLARLPVEPHIGKMILFGALF
XP_027320349.1  633 YFLSKLMDPPSRDAVMLAINHLMELNALDRQEELTPLGVHLARLPVEPHIGKMILFGALF
XP_013048508.1  555 YFLSKLMDPPSRDAVMLAINHLMELNALDRQEELTPLGVHLARLPVEPHIGKMILFGALF
NP_065916.2     653 YFLSRLMDPPSNEAVLLSIRHLMELNALDKQEELTPLGVHLARLPVEPHIGKMILFGALF
```

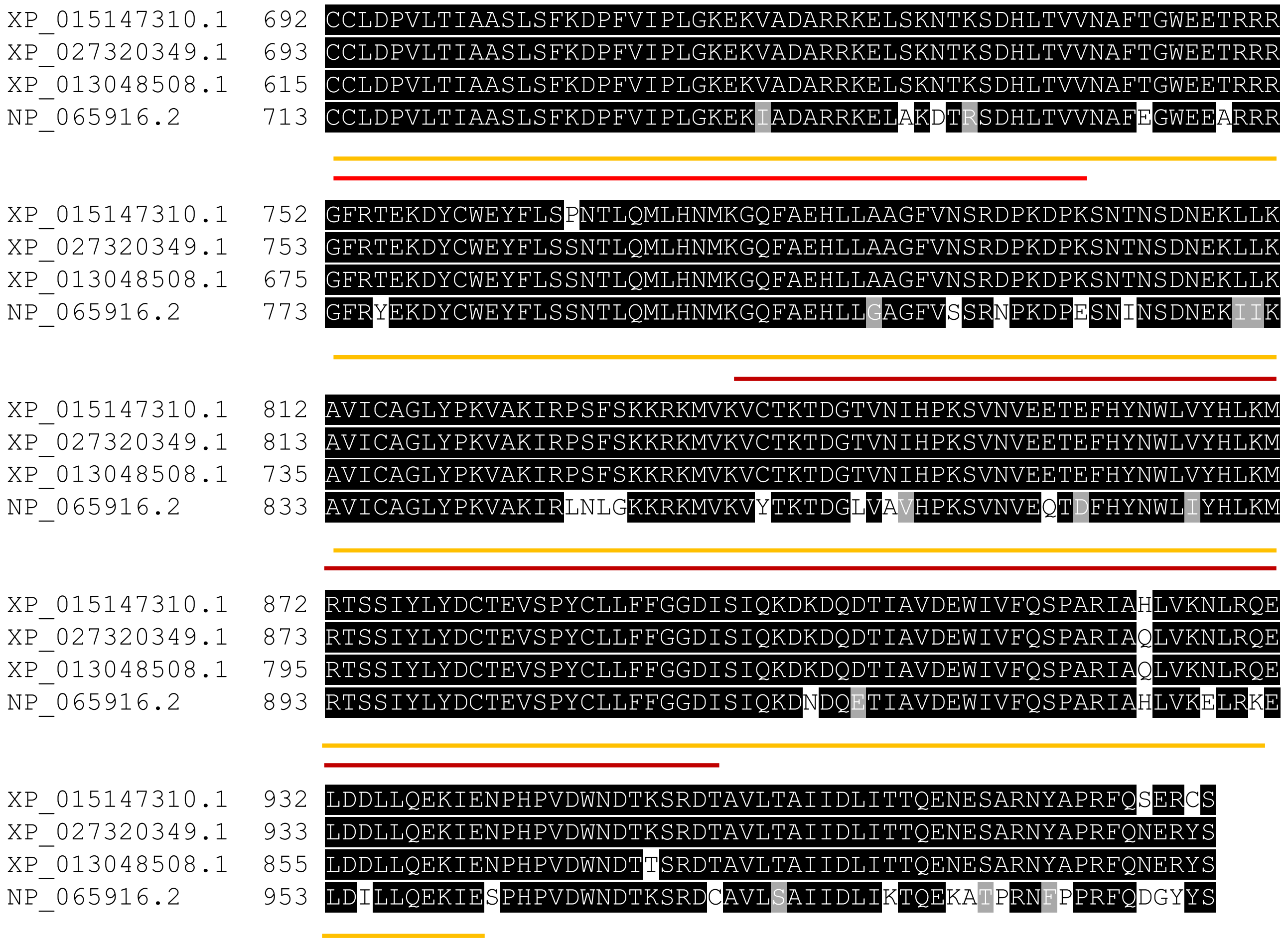

Figure S9: Schematic alignment of DHX36 protein sequences of chicken, duck, goose and human. Depicted are DEAD box helicase region, domain of unknown function (DUF), HA2-associated domain, HelicC domain in black, maroon, red and grey underlining respectively. The DExH box is boxed in blue.

```
NP_001025971.1    1 MSHVAVENALSLDQQFSGLDLNSSDSQSEGSATSKGRYIPPHLRNREASKQGF--DSGGW
XP_005012555.2    1 MEDLSR-------RMFSGLDLNSSDSQSEGSSTSKGRYIPPHLRNREASKQGF--DSGGW
XP_013046370.1    1 MEDLSR-------RMFSGLDLNSSDSQSEGSSTSKGRYIPPHLRNREASKQGF--DSGGW
NP_001347.3       1 MSHVAVENALGLDQQFAGLDLNSSDNQSGGSTASKGRYIPPHLRNREATKGFYDKDSSGW

NP_001025971.1   59 STSRDKDAYSSFGARSDRGAKSSFF-DRGNGSRGGRYEERGR------GSDYDRSGFGRF
XP_005012555.2   52 SSSRDKDAYSSFGVRSDRGAKSSFF-DRGNGSRGGRYEERGR------GSDYDRSGFGRF
XP_013046370.1   52 SSSRDKDAYSSFGVRSDRGAKSSFF-DRGNGSRGGRYEERGR------GSDYDRSGFGRF
NP_001347.3      61 SSSKDKDAYSSFGSRSDSRGKSSFFSDRGSGSRG-RFDDRGRSDYDGIGSRGDRSGFGKF

NP_001025971.1  112 DRGGNSRWCDKSDEDDWSKPLPPSERLEQELFSGSNTGINFEKYDDIPVEATGSNCPPHI
XP_005012555.2  105 DRGGNSRWSDKSDEDDWSKPLPPSERLEQELFSGSNTGINFEKYDDIPVEATGSNCPPHI
XP_013046370.1  105 DRGGNSRWSDKSDEDDWSKPLPPSERLEQELFSGSNTGINFEKYDDIPVEATGSNCPPHI
NP_001347.3     120 ERGGNSRWCDKSDEDDWSKPLPPSERLEQELFSGGNTGINFEKYDDIPVEATGNNCPPHI

NP_001025971.1  172 ESFSDVDMGEIIMGNIELTRYTRPTPVQKHAIPIIKEKRDLMACAQTGSGKTAAFLLPIL
XP_005012555.2  165 ESFSDVDMGEIIMGNIELTRYTRPTPVQKHAIPIIKEKRDLMACAQTGSGKTAAFLLPIL
XP_013046370.1  165 ESFSDVDMGEIIMGNIELTRYTRPTPVQKHAIPIIKEKRDLMACAQTGSGKTAAFLLPIL
NP_001347.3     180 ESFSDVEMGEIIMGNIELTRYTRPTPVQKHAIPIIKEKRDLMACAQTGSGKTAAFLLPIL

NP_001025971.1  232 SQIYADGPGDALRAMKENGRYGRRKQYPISLVLAPTRELAVQIYEEARKFAYRSRVRPCV
XP_005012555.2  225 SQIYADGPGDALRAMKENGRYGRRKQYPISLVLAPTRELAVQIYEEARKFAYRSRVRPCV
XP_013046370.1  225 SQIYADGPGDALRAMKENGRYGRRKQYPISLVLAPTRELAVQIYEEARKFAYRSRVRPCV
NP_001347.3     240 SQIYSDGPGEALRAMKENGRYGRRKQYPISLVLAPTRELAVQIYEEARKFSYRSRVRPCV

NP_001025971.1  292 VYGGADIGQQIRDLERGCHLLVATPGRLVDMMERGKIGLDFCKYLVLDEADRMLDMGFEP
XP_005012555.2  285 VYGGADIGQQIRDLERGCHLLVATPGRLVDMMERGKIGLDFCKYLVLDEADRMLDMGFEP
XP_013046370.1  285 VYGGADIGQQIRDLERGCHLLVATPGRLVDMMERGKIGLDFCKYLVLDEADRMLDMGFEP
NP_001347.3     300 VYGGADIGQQIRDLERGCHLLVATPGRLVDMMERGKIGLDFCKYLVLDEADRMLDMGFEP

NP_001025971.1  352 QIRRIVEQDTMPPKGVRHTMMFSATFPKEIQMLARDFLDEYIFLAVGRVGSTSENITQKV
XP_005012555.2  345 QIRRIVEQDTMPPKGVRHTMMFSATFPKEIQMLARDFLDEYIFLAVGRVGSTSENITQKV
XP_013046370.1  345 QIRRIVEQDTMPPKGVRHTMMFSATFPKEIQMLARDFLDEYIFLAVGRVGSTSENITQKV
NP_001347.3     360 QIRRIVEQDTMPPKGVRHTMMFSATFPKEIQMLARDFLDEYIFLAVGRVGSTSENITQKV
```

```
NP_001025971.1  412 VWVEELDKRSFLLDLLNATGKDSLTLVFVETKKGADALEDFLYHEGYACTSIHGDRSQRD
XP_005012555.2  405 VWVEESDKRSFLLDLLNATGKDSLTLVFVETKKGADALEDFLYHEGYACTSIHGDRSQRD
XP_013046370.1  405 VWVEESDKRSFLLDLLNATGKDSLTLVFVETKKGADALEDFLYHEGYACTSIHGDRSQRD
NP_001347.3     420 VWVEESDKRSFLLDLLNATGKDSLTLVFVETKKGADSLEDFLYHEGYACTSIHGDRSQRD

NP_001025971.1  472 REEALHQFRSGKSPILVATAVAARGLDISNVKHVINFDLPSDIEEYVHRIGRTGRVGNLG
XP_005012555.2  465 REEALHQFRSGKSPILVATAVAARGLDISNVKHVINFDLPSDIEEYVHRIGRTGRVGNLG
XP_013046370.1  465 REEALHQFRSGKSPILVATAVAARGLDISNVKHVINFDLPSDIEEYVHRIGRTGRVGNLG
NP_001347.3     480 REEALHQFRSGKSPILVATAVAARGLDISNVKHVINFDLPSDIEEYVHRIGRTGRVGNLG

NP_001025971.1  532 LATSFFNERNINITKDLLDLLVEAKQEVPSWLENMAYEQHHKGGGSRGRSKS-RFTGGFG
XP_005012555.2  525 LATSFFNERNINITKDLLDLLVEAKQEVPSWLENMAYEQHHKGGGSRGRSKSSRFSGGFG
XP_013046370.1  525 KYGQGFV-------PNLLGVVLLAKWHTE-------------------------------
NP_001347.3     540 LATSFFNERNINITKDLLDLLVEAKQEVPSWLENMAYEHHYKG-SSRGRSKSSRFSGGFG

NP_001025971.1  591 ARDYRTSSGAGSSSFSSSRPASGRTGGSG---SRGFGGGGYGGFYNSDGYGGNYNSQGVD
XP_005012555.2  585 ARDYRTSSGSGSSSFSSSRSTSSRSGGSG---SRGFGGGGYGGFYNSDGYGGNYNSQGVD
XP_013046370.1  547 -----TTECLGKACVPS-----------V---TRQYC-----------------------
NP_001347.3     599 ARDYRQSSGASSSSFSSSRASSSRSGGGGHGSSRGFGGGGYGGFYNSDGYGGNYNSQGVD

NP_001025971.1  648 WWGN
XP_005012555.2  642 WWGN
XP_013046370.1      ----
NP_001347.3     659 WWGN
```

Figure S10: Schematic alignment of DDX3X protein sequences of chicken, duck, goose and human. Well conserved domains include DEAD box helicase region (blue box), HelicC and C-terminal arginine-serine (RS) rich region. Note variation in RS region in goose DDX3

```
XP_025001441.1    1 MA-----------------------------------GELTDKKDR---------------
XP_021135293.1    1 MDKTINVRLLLIEKIINWTDLGRWGIPGPAQGRPRPEEVADKKDR---------------
XP_013054789.1    1 MP-----------------------------------ASCPQWKTEILDYCPTTRVLLIGC
NP_004809.2       1 MA-----------------------------------GELADKKDR---------------

XP_025001441.1   12 ------DASPVKEERKRSRSPDRDRD-RDRDRKGSPPKDRKRHSRDRRR-GSRSRSRSR
XP_021135293.1   46 ------DASPVKEERKRSRSPDRD---RDRDRKGSPGKDRKRHSRDRAA--SCSRSRSR
XP_013054789.1   27 KTDLRTDLSTLMELSHQKQAPISYEQPPGLCSGGIPPVGGVGV------GQG--LRSVP-
NP_004809.2      12 ------DASPSKEERKRSRTPDRERD-RDRDRKSSPSKDRKRHSRDRRRGGSRSRSRSR

XP_025001441.1   64 SKSLDRDRRHKERD----RDRGKKDREREKDGHRRDKDRKRSSLSPSRGKDSKSRKERDS
XP_021135293.1   95 SKSVDS---------------------------RRDKDRKRSSLSPSRGKDSKSRKERDS
XP_013054789.1   78 ----------------------------------NFPPVLLGSLSPSRGKDSKSRKERDL
NP_004809.2      65 SKSAERERRHKERERDKERDRNKKDRDRDKDGHRRDKDRKRSSLSPGRGKDFKSRKDRDS

XP_025001441.1  120 RKAEEEEENALKKEKAQPLSLEELLAKKKAEEEAEAKPKFLSKAEREAEALRRRQQEVE-
XP_021135293.1  128 RKLEEEEENALKKEKAQPLSLEELLAKKKAEEEAEAKPKFLSKAEREAEALRRRQQEVEE
XP_013054789.1  104 RKAEEEEENALKKEKVR-------------------TPRSLPASPWGAGALLRSFPC---
NP_004809.2     125 KKDE-EDEHGDKKPKAQPLSLEELLAKKKAEEEAEAKPKFLSKAEREAEALKRRQQEVE-

XP_025001441.1  179 ERQRLLEEERKKRKQFQEMGRKMLEDPQERERRERRERMERETNGTEDEEGRQKIREEKD
XP_021135293.1  188 ERQRLLEEERKKRKQFQEMGRKMLEDPQERERRERRERMERETNGTEDEEGRQKIREEKD
XP_013054789.1  142 ----------------------SPEDPQERERRERRERMERETNGTEDEEGRQKIREEKD
NP_004809.2     183 ERQRMLEEERKKRKQFQDLGRKMLEDPQERERRERRERMERETNGNEDEEGRQKIREEKD

XP_025001441.1  239 KSKELHAIKERYLGGVKKRRRTRHLNDRKFVFEWDASEDTSIDYNPLYKERHQVQLLGRG
XP_021135293.1  248 KSKELHAIKERYLGGVKKRRRTRHLNDRKFVFEWDASEDTSIDYNPLYKERHQVQLLGRG
XP_013054789.1  180 KSKELHAIKERYLGGVKKRRRTRHLNDRKFVFEWDASEDTSIDYNPLYKERHQVQLLGRG
NP_004809.2     243 KSKELHAIKERYLGGIKKRRRTRHLNDRKFVFEWDASEDTSIDYNPLYKERHQVQLLGRG

XP_025001441.1  299 FIAGIDLKQQKREQSRFYGDLMEKRRTLEEKEQEEARLRKLRKKEAKQRWDDRHWSQKKL
XP_021135293.1  308 FIAGIDLKQQKREQSRFYGDLMEKRRTLEEKEQEEARLRKLRKKEAKQRWDDRHWSQKKL
XP_013054789.1  240 FIAGIDLKQQKREQSRFYGDLMEKRRTLEEKEQEEARLRKLRKKEAKQRWDDRHWSQKKL
NP_004809.2     303 FIAGIDLKQQKREQSRFYGDLMEKRRTLEEKEQEEARLRKLRKKEAKQRWDDRHWSQKKL
```

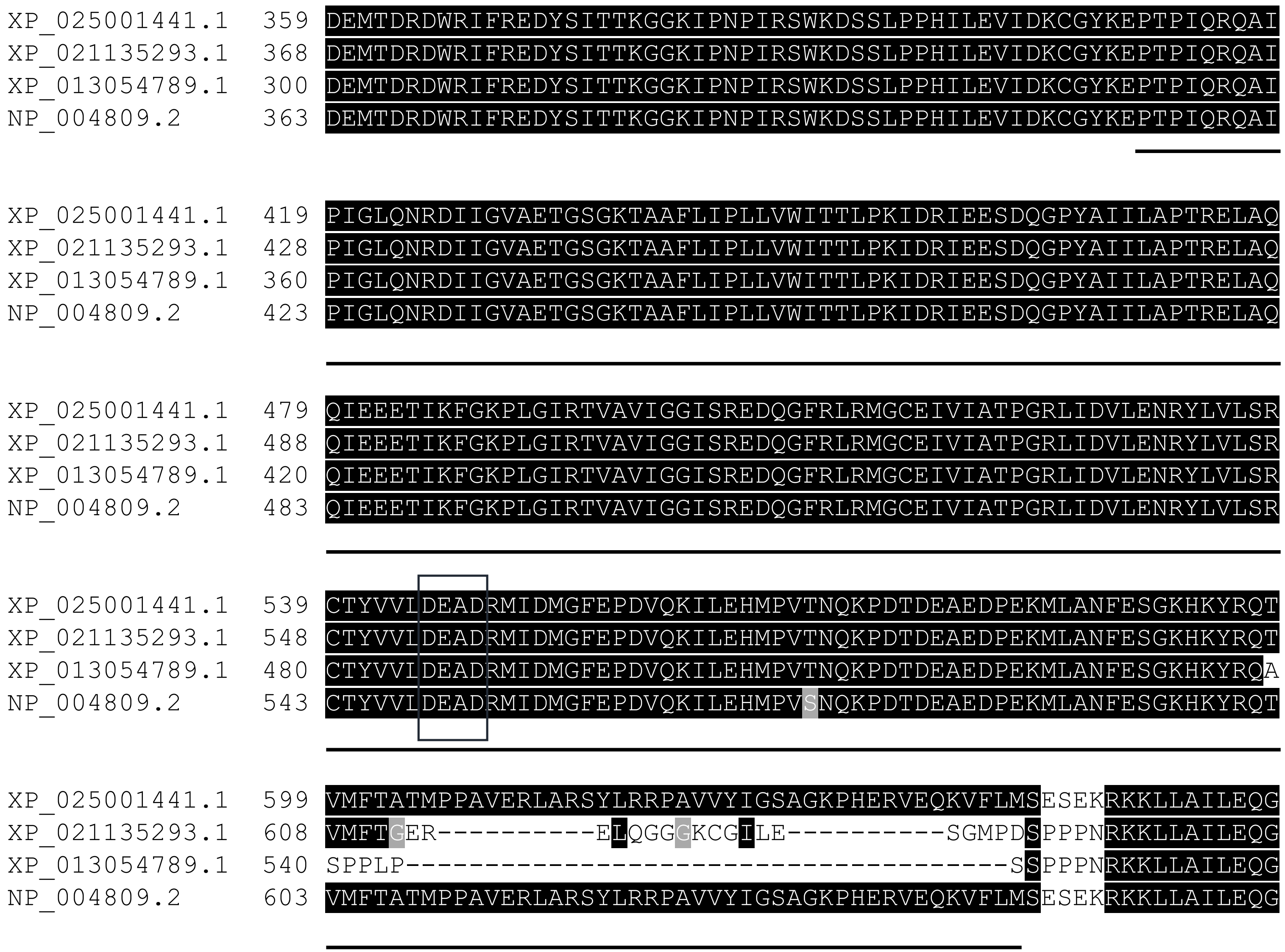

XP_025001441.1 359 DEMTDRDWRIFREDYSITTKGGKIPNPIRSWKDSSLPPHILEVIDKCGYKEPTPIQRQAI
XP_021135293.1 368 DEMTDRDWRIFREDYSITTKGGKIPNPIRSWKDSSLPPHILEVIDKCGYKEPTPIQRQAI
XP_013054789.1 300 DEMTDRDWRIFREDYSITTKGGKIPNPIRSWKDSSLPPHILEVIDKCGYKEPTPIQRQAI
NP_004809.2 363 DEMTDRDWRIFREDYSITTKGGKIPNPIRSWKDSSLPPHILEVIDKCGYKEPTPIQRQAI
XP_025001441.1 419 PIGLQNRDIIGVAETGSGKTAAFLIPLLVWITTLPKIDRIEESDQGPYAIILAPTRELAQ
XP_021135293.1 428 PIGLQNRDIIGVAETGSGKTAAFLIPLLVWITTLPKIDRIEESDQGPYAIILAPTRELAQ
XP_013054789.1 360 PIGLQNRDIIGVAETGSGKTAAFLIPLLVWITTLPKIDRIEESDQGPYAIILAPTRELAQ
NP_004809.2 423 PIGLQNRDIIGVAETGSGKTAAFLIPLLVWITTLPKIDRIEESDQGPYAIILAPTRELAQ
XP_025001441.1 479 QIEEETIKFGKPLGIRTVAVIGGISREDQGFRLRMGCEIVIATPGRLIDVLENRYLVLSR
XP_021135293.1 488 QIEEETIKFGKPLGIRTVAVIGGISREDQGFRLRMGCEIVIATPGRLIDVLENRYLVLSR
XP_013054789.1 420 QIEEETIKFGKPLGIRTVAVIGGISREDQGFRLRMGCEIVIATPGRLIDVLENRYLVLSR
NP_004809.2 483 QIEEETIKFGKPLGIRTVAVIGGISREDQGFRLRMGCEIVIATPGRLIDVLENRYLVLSR
XP_025001441.1 539 CTYVVLDEADRMIDMGFEPDVQKILEHMPVTNQKPDTDEAEDPEKMLANFESGKHKYRQT
XP_021135293.1 548 CTYVVLDEADRMIDMGFEPDVQKILEHMPVTNQKPDTDEAEDPEKMLANFESGKHKYRQT
XP_013054789.1 480 CTYVVLDEADRMIDMGFEPDVQKILEHMPVTNQKPDTDEAEDPEKMLANFESGKHKYRQA
NP_004809.2 543 CTYVVLDEADRMIDMGFEPDVQKILEHMPVSNQKPDTDEAEDPEKMLANFESGKHKYRQT
XP_025001441.1 599 VMFTATMPPAVERLARSYLRRPAVVYIGSAGKPHERVEQKVFLMSESEKRKKLLAILEQG
XP_021135293.1 608 VMFTGER----------ELQGGGKCGILE----------SGMPDSPPPNRKKLLAILEQG
XP_013054789.1 540 SPPLP--------------------------------------SSPPPNRKKLLAILEQG
NP_004809.2 603 VMFTATMPPAVERLARSYLRRPAVVYIGSAGKPHERVEQKVFLMSESEKRKKLLAILEQG

```
XP_025001441.1  659 FDPPIIIFVNQKKGCDVLAKSLEKMGYNACTLHGGKGQEQREFALSNLKAGAKDILVATD
XP_021135293.1  648 FDPPIIIFVNQKKGCDVLAKSLEKMGYNACTLHGGKGQEQREFALSNLKAGAKDILVATD
XP_013054789.1  562 FDPPIIIFVNQKKGCDVLAKSLEKMGYNACTLHGGKGQEQREFALSNLKAGAKDILVATD
NP_004809.2     663 FDPPIIIFVNQKKGCDVLAKSLEKMGYNACTLHGGKGQEQREFALSNLKAGAKDILVATD

XP_025001441.1  719 VAGRGIDIHDVSMVVNYDMAKNIEDYIHRIGRTGRAGKSGVAITFLTKEDSTVFYDLKQA
XP_021135293.1  708 VAGRGIDIHDVSMVVNYDMAKNIEDYIHRIGRTGRAGKSGVAITFLTKEDSTVFYDLKQA
XP_013054789.1  622 VAGRGIDIHDVSMVVNYDMAKNIEGESGAWYFTYS-----V---LLSLLACSVFLH----
NP_004809.2     723 VAGRGIDIQDVSMVVNYDMAKNIEDYIHRIGRTGRAGKSGVAITFLTKEDSAVFYELKQA

XP_025001441.1  779 ILESPVSSCPPELANHPDAQHKPGTILTKKRREETIFA----------------------
XP_021135293.1  768 ILESPVSSCPPEXXXX-XXXXXPGTILTKKRREETIFA----------------------
XP_013054789.1  670 -------------------ISSIGKLLLMVAIEATYLVLVEGPQAALFDNADLLVVANAL
NP_004809.2     783 ILESPVSSCPPELANHPDAQHKPGTILTKKRREETIFA----------------------
```

Figure S11: Schematic alignment of DDX23 protein sequences of chicken, duck, goose and human. Depicted are the putative nuclear localization signal sequences (NLSs), DEAD box region, and HelicC domain in grey, black, and red underlining respectively. The DEAD box is boxed in blue.

```
XP_025006699.1    1  MKFRRG--R-RFTSSAKLRRKGIEVLGKWKTVEIDPNVFVDEQFKDVVCLEELTEYKLVS
XP_027314331.1    1  MKAKKG--R-RFTSSVKLKRKGIAVIGKWKTVEIDPNLFADEQFRDIVCLEELTEYKLVN
XP_013037286.1    1  MKAKKG--R-RFTSSVKLKRKGIEVIGKWKTVEIDPNLFADEQFRDIVCLEELTEYKLVS
NP_065147.1       1  MKLKDTKSRPKQSSCGKFQTKGIKVVGKWKEVKIDPNMFADGQMDDLVCFEELTDYQLVS

XP_025006699.1   58  SDKVGKG----NKQKRKSEDLSEEGSEEEEEEEEAVIPPKKKKKCKDLKTRADKSDDSN-
XP_027314331.1   58  SNKVGQV----SGQKRKVKSLSE----GSEEEEEPVVPPKKKKKGKDLKTKTDTGDDTN-
XP_013037286.1   58  SNKVGKV----NEQKRKAQSLSE----GSGEEEEPVVPPKKKKKSKDLKTRTDKSDDTN-
NP_065147.1      61  PAKNPSSLFSKEAPKRKAQAVSE----EEEEEEGKSSSPKKKIKLKKSKNVATEGTSTQK

XP_025006699.1  113  ATEVDVPVDEEKRCEEIVQGADDKVCGH-TAESTSS---TKNAAKKNKKKEKEKVVKNKA
XP_027314331.1  109  ATEVDVPVDEETRCEEIIEGANCEDSGH-TAGSTSS---TKSAVKKKK----KKVDKNKT
XP_013037286.1  109  ATEVDVPVDEETKCEEITEGANREDNGH-RAESTSS---TKSAVKKKK----KKVDKNKT
NP_065147.1     117  EFEVKDPE--------LEAQGDDMVCDDPEAGEMTSENLVQTAPKKKK----NKG---KK

XP_025006699.1  169  SQVQDASPPVTTSKKVKNWTTEVLSTSTDHKADVSAWKDLFVPQPVLKALSSLGFSAPTP
XP_027314331.1  161  SQDQEALPSVTTSKKLKNWSTEVLSASTDHKADVSAWKDLFVPKPVLKALSYLGFSAPTP
XP_013037286.1  161  SQDQEALPSVTTSKKVKNWSTEVLSASSDHKADVSAWKDLFVPKPVLKALSYLGFSAPTP
NP_065147.1     162  GLEPSQSTAAKVPKKAKTWIPEV----HDQKADVSAWKDLFVPRPVLRALSFLGFSAPTP

XP_025006699.1  229  IQALTLPSAIRDNMDILGAAETGSGKTLAFAIPMIHSVLEWQKSNSSASVSDSVSKKP--
XP_027314331.1  221  IQALALPSAIRDNMDVLGAAETGSGKTLAFAIPMIHSVLEWQKSNSSMSRNDSVSKES--
XP_013037286.1  221  IQALALPSAIRDNMDVLGAAETGSGKTLAFAIPMIHSVLEWQKSNSSMSRNDSVSKES--
NP_065147.1     218  IQALTLAPAIRDKLDILGAAETGSGKTLAFAIPMIHAVLQWQKRNAAPPPSNTEAPPGET

XP_025006699.1  287  -YQHHDEMRWENDDEAE-----------------KPN-----------HQQVEDSGDEDD
XP_027314331.1  279  -HQHHDETRWENEDEAE-----------------KLT-----------HQQVEDSGDEDD
XP_013037286.1  279  -HQHHDETRWENEDEAE-----------------KLT-----------HQQVEDSGDEDD
NP_065147.1     278  RTEAGAETRSPGKAEAESDALPDDTVIESEALPSDIAAEARAKTGGTVSDQALLFGDDDA

XP_025006699.1  318  ASFATGCVK------VQ------------ENTEYDSSNKEHTVGLHKKRPLLGLVLTPTR
XP_027314331.1  310  ASFATGCVK------VL------------ENAEFDSSDKGHAVGLHKKRPLLGLVLTPTR
XP_013037286.1  310  ASFATGCVK------VL------------ENAEFDSSDKGHTVGLHKKRPLLGLVLTPTR
NP_065147.1     338  GEGPSSLIREKPVPKQNENEEENLDKEQTGNLKQELDDKSATCKAYPKRPLLGLVLTPTR
```

```
XP_025006699.1  360 ELAVQVKHHIDAVAKFTGIKTAILVGGMAAQKQERVLNRKPEIVIATPGRLWELIKERHP
XP_027314331.1  352 ELAVQVKHHIDAVTKFTGIKTAILVGGMAAQKQERVLNRKPEIVIATPGRLWELVKAKHP
XP_013037286.1  352 ELAVQVKHHIDAVTKFTGIKTAILVGGMAAQKQERVLNRKPEIVIATPGRLWELVKAKHP
NP_065147.1     398 ELAVQVKQHIDAVARFTGIKTAILVGGMSTQKQQRMLNRRPEIVVATPGRLWELIKEKHY

XP_025006699.1  420 HLSNLRQLRCLVIDEADRMVERGHFLELSQLLEVLNDSQYNPKRQTFVFSATLTLVHQTP
XP_027314331.1  412 HLSNLRQLRCLVIDEADRMVERGHFLELSQLLELLNDSQYNPKRQTFVFSATLTLVHQTP
XP_013037286.1  412 HLSNLRQLRCLVIDEADRMVERGHFLELSQLLELLNDSQYNPKRQTFVFSATLTLVHQTP
NP_065147.1     458 HLRNLRQLRCLVVDEADRMVEKGHFAELSQLLEMLNDSQYNPKRQTLVFSATLTLVHQAP

XP_025006699.1  480 ARILQKKNAKKIDKKTKLEMLMEKVGIKGKPKVIDLTRKEATVETLMETRIHCDTNEKDY
XP_027314331.1  472 TRVLQKKNAKKMDKKTKLEMLMEKVGIKGKPKVIDLTRKEATVETLTETRIHCDTNEKDY
XP_013037286.1  472 TRVLQKKNAKKMDKKTKLEMLMEKVGIKGKPKVIDLTRKEATVETLTETRIHCDTNEKDY
NP_065147.1     518 ARILHKKHTKKMDKTAKLDLLMQKIGMRGKPKVIDLTRNEATVETLTETKIHCETDEKDF

XP_025006699.1  540 YLYYFLLQYPGRTMVFANSIDCIKRLSSLLTILNCDPFPLHANMHQKQRLKNLERFAERE
XP_027314331.1  532 YLYYFLLQYPGRTMVFANSIDCIKRLSSLLSILNCDPLPLHANMHQKQRLKNLERFAERE
XP_013037286.1  532 YLYYFLLQYPGRTMVFANSIDCIKRLSSLLSILNCDPLPLHANMHQKQRLKNLERFAERE
NP_065147.1     578 YLYYFLMQYPGRSIVFANSISCIKRLSGLLKVLDIMPLTLHACMHQKQRLRNLEQFARLE

XP_025006699.1  600 SCVLLTTDVAARGLDIPNVQHVIHYQVPRTSELYVHRSGRTARAANEGLSLLLIGPDDLI
XP_027314331.1  592 SCVLLTTDVAARGLDIPNVQHVIHYQVPRTSELYVHRSGRTARAANEGLSLLLIGPDDLI
XP_013037286.1  592 SCVLLTTDVAARGLDIPNVQHVIHYQVPRTSELYVHRSGRTARAANEGLSLLLIGPDDLI
NP_065147.1     638 DCVLLATDVAARGLDIPKVQHVIHYQVPRTSEIYVHRSGRTARATNEGLSLMLIGPEDVI

XP_025006699.1  660 NFRKIYKTLQKSEELPLFPVETKYMTAIKERVNLARQIEKAEFFNGRAKQHDSWLQQAAE
XP_027314331.1  652 NFRKIYKTLEKSEELPFFPVETKYMTSIKERMNLARQIEKAEFFNSRAKQHDSWLQQAAE
XP_013037286.1  652 NFRKIYKTLEKSEELPFFPVETKYMTSIKERMNLARQIEKAEFFNSRAKQHDSWLQQAAE
NP_065147.1     698 NFKKIYKTLKKDEDIPLFPVQTKYMDVVKERIRLARQIEKSEYRNFQACLHNSWIEQAAA

XP_025006699.1  720 ALEVDLDDDLLMGKKTSEQEESQKQKMLKGMKKQLKHMLSQPLFKVFMKTKYPTQSGKLL
XP_027314331.1  712 ALEVDLDDDLFMGRKTSEQEESQKQKMLKGMKKQLKHMLSQPLFKVFMKTKYPTQSGKLL
XP_013037286.1  712 ALEVDLDDDLFMGRKASEQEESQKQKMLKGMKKQLKHMLSQPLFKVFMKTKYPTQSGKLL
NP_065147.1     758 ALEIELEEDMYKGGKADQQEERRRQKQMKVLKKELRHLLSQPLFTESQKTKYPTQSGKPP
```

```
XP_025006699.1  780 LPQTSAGNSETALSIISKKQARRKK------------SVKSN
XP_027314331.1  772 LPQTSVGSSESALGTVSKQQAKKNK------------SKK-N
XP_013037286.1  772 LPQSSAGNSESALGTVSKQHAKKNK------------SKTIR
NP_065147.1     818 LLVSAPSKSESALSCLSKQKKKKTKKPKEPQPEQPQPSTSAN
```

Figure S12: Schematic alignment of DDX24 protein sequences of chicken, duck, goose and human. The DEAD box is boxed in blue.

```
XP_004940975.1    1 -----------------------------------------------MDSAEDNFK--IH
XP_027312235.1    1 -----------------------------------------------MDSAEDNFKKVVC
XP_013030722.1    1 MGGGSTLKEVCWQAQWRTGLCCRSQPGRALLEAACRVAAGAGESRSVMDSAEDNFKHVVS
NP_060101.3       1 -----------------------------------------------ME-----------

XP_004940975.1   12 DYANES---ADSDDKDSECVDSEKINFTATEEDKE--STSDRDEEDVTDHNEEAAVVQML
XP_027312235.1   14 DTTNGSTSKDDSDDMDSDVIDSEEIGCISTKKNKEGGSTSDHDEGDETSDKAEAAVVQML
XP_013030722.1   61 DNTNGSTSKDDSDDMDSECIDSEEIGFISRKKNKEGGSTSDHDEEDETNDNAEAAVMQML
NP_060101.3       3 ------------------------------------------------------------

XP_004940975.1   67 KEKEDREKEAEDPDYGEIYSILNTDEVIQKESQNEGRNFRVLQNKLNSQDPEVRKHLEIL
XP_027312235.1   74 KEKEDKEKEVEDPDYGKAYNILTTEEVAQKESQTEGKDFRVLQNEWDSKSPEIRKHLEFL
XP_013030722.1  121 KEKEDREKDVEDPDYDNVYNILTTEEV---ESHTAGKDFRFLQDEWDSQSPEIRKHLEFL
NP_060101.3       3 ------------------RNVLTT----------------------------------FS

XP_004940975.1  127 KKLKQSIWPSLSKASFVSLLKDFVESEFFVIDGDSLILTFLNE----EVQHLTLFYLIEC
XP_027312235.1  134 KILKLHVWPSLSRARYVSLLKDFVESEFFVIDGDSLILMLLNE----EVQYLTLFYRIEC
XP_013030722.1  178 KILKLHVWSSLSRARYVSLLKDFVESEFFVIDGDSLILMLLNE----EVQYLNLFYLIEC
NP_060101.3      11 QEMSQLILNEMPKAEYSSLFNDFVESEFFLIDGDSLLITCICEISFKPGQNLHFFYLVER

XP_004940975.1  183 FLHDFTQKGAKYIIVFFKDAEQMYFQNPQFLFLRTALVEHLKHNTNITIHTEFSNCFSQE
XP_027312235.1  190 FLQDFTQRGARYIVVFFKDAEQMYFRYPHFLFLRTALIEHLKHNTRTTVHTEFSNCLSPA
XP_013030722.1  234 FLQDFTQKGAKYIVVFFKDAEQMYFPYPHFLFLRTALIEHLKHNTRTTVHTEFSNCLSPA
NP_060101.3      71 YLVDLISKGGQFTIVFFKDAEYAYFNFPELLSLRTALILHLQKNTTIDVRTTFSRCLSKE

XP_004940975.1  243 WEIFLKKSYPYFIIISDIGLTSLQTNYLTIFIAHSLSKKINVVQAFGQECDTLRVYGYHL
XP_027312235.1  250 WEVFLKKSYPYFIIVSDIGLTSLQTNYLTIFIAHSLSKKITIVLASGQECDTLRVYGYHL
XP_013030722.1  294 WEIFLKKSYPYFIIVSDIGLTCPQTNYLTIFIAHSLSKKITIVLASGQEYDTLRVYGYHL
NP_060101.3     131 WGSFLEESYPYFLIVADEGLNDLQTQLFNFLIIHSWARKVNVVLSSGQESDVLCLYAYLL

XP_004940975.1  303 QSMWKHRIFFQKYGKDLQSAC----EAIVRYQELSLY-LYEHLKLSLKSIQNEVYQTVLL
XP_027312235.1  310 QSMAKHRLFFQKYEKDLRCVC----EAIVRYKEPSLY-LYGHLKLSLKSVQEEVCRTIPL
XP_013030722.1  354 QSIRKHRLFFQKYEKDLQCLC----EAIVRYKEPSLC-LYGHLRLSLKSVQEEVCQTIPL
NP_060101.3     191 PSMYRHQIFSWKNKQNIKDAYTTLLNQLERFKLSALAPLFGSLK--WNNITEEAHKTVSL
```

```
XP_004940975.1  358 LKKLWPEGSDIRRVICVLTCAVGLKLYSDALESAHASLGAKPEQSESERSKPLSPEEATD
XP_027312235.1  365 LKQLWPEGSDIRRVVCVLACAVGLKIYSDMLTNAHASRGTKTEKSESGRSKPPSPEAAAD
XP_013030722.1  409 LKQLWPEGSDIRRVVCVLACAVGLKIYSDMLKNAHASRGTKTEKSERGRSKPLSPEAAAD
NP_060101.3     249 LTQVWPEGSDIRRVFCVTSCSLSLRMYHRFLGNREPSSGQETEIQQ-VNSNCLTLQEMED

XP_004940975.1  418 LCRMQCLTMVFLLHMPLSQRAQIRIMKSCWTEQVLPLIKMQQLCAHFVLKQLSDINDWKL
XP_027312235.1  425 LCRMQCLTVVFLLHMPLSQRAQIRVMKSCWTKQVLPIMKMQQLCVHFVLKELSDKNDWKL
XP_013030722.1  469 LCRMQCLTVVFLLHMPLSQRAQIRVMKSCWTKQVLPIIKMQQLCAHFVLKQLSDKNDWKL
NP_060101.3     308 LCKLHCLTVVFLLHLPLSQRACARVITSHWAEDMKPLLQMKKWCEYFILRNIHTFEFWNL

XP_004940975.1  478 DLTYLPDLNDDSLLRNLAYYYEIEYSEGLEFQKELGKEMDKEYQFLWDTVTKLTVNYDFG
XP_027312235.1  485 DLTYLPDLNDESLLKNLAHYYQIEYTKGLEFQKELGKEMEDEYLSLWDAVTKLTVKYGFG
XP_013030722.1  529 DLTYLPDLSDDSLLINLAHYYEIEYSEGLEFQKELGKEMEDQYRSLWDAVTKLTVKYGFG
NP_060101.3     368 NLIHLSDLNDELLLKNIAFYYENENVKGLHL--NLGDTIMKDYEYLWNTVSKLVRDFEVG

XP_004940975.1  538 DAFPVRNTSQVFIDHQQTSFRV-CDEKIPDIGLIPLKSDLAEEYAGDVLKDLPVLESNDP
XP_027312235.1  545 DAFPIRTTSQVFLDQEQTSPRV-YEDKIPAIGLFPVKSDLVKEYAGDILKDLPVLDDNDP
XP_013030722.1  589 DAFPIRTTSQLFLDQEQTSFRV-YEDKIPAIGLFPVKSDLVKEYAGDILKDLPVLDDNDP
NP_060101.3     426 QPFPLRTTKVCFLEKKPSPIKDSSNEMVPNLGFIPTSSFVVDKFAGDILKDLPFLKSDDP

XP_004940975.1  597 AVTSLAKYKEFDELRHWHSGRPLSEEYERVRSNADAKSKDADERKQIQKLQTFYRLYGST
XP_027312235.1  604 AVSSLTKYKEFDELRHWHSGRPLTEEYERVHCNADAKSKDPYERKQIQKLQTFYRFYGST
XP_013030722.1  648 AVSSLTKYKEFDEHRHWHSGRPLTEEYERVHCNADAKSKDPYERKQIQKLQTFYRFYGST
NP_060101.3     486 IVTSLVKQKEFDELVHWHSHKPLSDDYDRSRCQFDEKSRDPRVLRSVQKYHVFQRFYGNS

XP_004940975.1  657 LEGSTSKFIVHQNDVPCSNTAVKKAKQIHKSKAEIIAEENNRRLKAKEEKKEEEQWKTLQ
XP_027312235.1  664 LQEGTSKFVVCQKNAPGSANNPVKKKKMQKSKAEIIAEENNKRLKIKEEMKEQEQWKTLQ
XP_013030722.1  708 LQEGTSKFIVCQKDAAGSANNPVKKKKMQKSKAEIIAEENNKRLKIKEEMKEQEQWKTLQ
NP_060101.3     546 LETVSSKIIVTQTIKSKKDFSGPKSKKAHETKAEIIARENKKRLFAREEQKEEQKWNALS

XP_004940975.1  717 ITTEKEIKANLTVGINKMEKFLKTVKSKSVKFSVEMSALSACVEAWKKHCRKQ-GNKPKD
XP_027312235.1  724 ISTEKEIKANLTVGINKLEKFLKNLKSKSVKFSVELSGLSACLEVWKEHCKKQ-DTKSKD
XP_013030722.1  768 MSTEKEIKENLTVGINKLEKFLKILKSKSVKFSVELSGLSACLEAWKEHCKEQ-ATKSKD
NP_060101.3     606 FSIEEQLKENLHSGIKSLEDFLKSCKSSCVKLQVEMVGLTACLKAWKEHCRSEEGKTTKD
```

```
XP_004940975.1  776 LSSAVQLMRRIHILLEKYQDLLEKSHLQKLSQYLQLLGFENLACSLTGQMREGRDD--QN
XP_027312235.1  783 LSKAVQLMRRIHILLEKYQDLLEKSHLQKLTQCLRLLGFENLACSLSGQVREKSDG--EN
XP_013030722.1  827 LSKAVQLMRRIHILLEKYQDLLEKPHLQKLTQCLRLLGFENLACSLSGQVREKSDG--EN
NP_060101.3     666 LSIAVQVMKRIHSLMEKYSELLQEDDRQLIARCLKYLGFDELASSLHPAQDAENDVKVKK

XP_004940975.1  834 RSNYDVEVGPARFQLQYMDYYLLREERNDPDPRVQHFIPDTWQRELLDAVDNNESAVIVA
XP_027312235.1  841 TSRYAIEVGPVRFQLQYMDYYLLREERNDPDPRVENFIPDTWQRELLDAVDNNESAVIVA
XP_013030722.1  885 TSRYAIEVGPVRFQLQYMDYYLLREERNDPDPRVENFIPDTWQRELLDAVDNNESAVIVA
NP_060101.3     726 RNKYSVGIGPARFQLQYMGHYLIRDERKDPDPRVQDFIPDTWQRELLDVVDKNESAVIVA

XP_004940975.1  894 PTSSGKTYASYYCMEKVLKMSDEGVVVYVAPTKALVNQVVGSVYSRFTKKLPDGLVVCGV
XP_027312235.1  901 PTSSGKTYASYYCMEKVLKTSDDGVVVYVAPTKALVNQVVGTVYSRFTKTLPDGLVVCGV
XP_013030722.1  945 PTSSGKTYASYYCMEKVLKTSDDGVVVYVAPTKALVNQVVGTIYSRFTKKLPDGLVVCGV
NP_060101.3     786 PTSSGKTYASYYCMEKVLKESDDGVVVYVAPTKALVNQVAATVQNRFTKNLPSGEVLCGV

XP_004940975.1  954 FTRDYRHDVMNSQILVTVPQCLEILMLSPRCQSWTKRIQYII-------FDEIHCLGGEI
XP_027312235.1  961 FTRDYRNDVMNSQILVTVPQCLXXXXVLX----XGRFITFLFFFLSSFPNIXVHCLGGEI
XP_013030722.1 1005 FTRDYRNDVMNSQILVTVPQCLEILMLSPRCQKWVKRIQYVI-------FDEVHCLGSEI
NP_060101.3     846 FTREYRHDALNCQVLITVPACFEILLLAPHRQNWVKKIRYVI-------FDEVHCLGGEI

XP_004940975.1 1007 GAEVWEHLLVTIRCPFLALSATISNPDHLTEWLQSVKRYWQCADNTIKGSSTNSEKNFAR
XP_027312235.1 1017 GAEVWEHLLVTIRCPFLALSATISNPEHLTEWLQSVKRYWQRAENMIEENSTNFEKNSTR
XP_013030722.1 1058 GAEVWEHLLVTIRCPFLALSATISNPEHLTEWLQSVKRYWQRAENTIEESPTSSEKNSTR
NP_060101.3     899 GAEIWEHLLVMIRCPFLALSATISNPEHLTEWLQSVKWYWKQEDKIIE-NNTASKRHVGR

XP_004940975.1 1067 KCKGTSTVKEQKKSYRVRLVLYEERYNDLEKYVCSVNGSDFGIEHFHPCAALTVNHIENY
XP_027312235.1 1077 KLKVKSKVKEPKKTYRVRLVLYGERYNDLEKYVCSSTGGDFLFEHYHPCAALTVNHIENY
XP_013030722.1 1118 KLKVKSKVKEPKKSYRVRLVLYGERYNDLEKYVCSSTGGDFLIEHYHPCAALTVNHIENY
NP_060101.3     958 QAGFPKDYLQVKQSYKVRLVLYGERYNDLEKHVCSIKHGDIHFDHFHPCAALTTDHIERY

XP_004940975.1 1127 GIPSDLSLSPRESIQLYDTMVKVWQKWPRAQEMDPEEFVSFKNNIVIKKMDVRKYEQELK
XP_027312235.1 1137 GIPADLSLSPKESVQLYDTMVEVWKEWPRAQELDPEEFVSFKNKVVIKKMDVRKYEQELK
XP_013030722.1 1178 GIPADLSLSPRESVQLYDTMVEVWNEWPRAQELDPEEFVSFKNKVVIKKTDVRKYEQELK
NP_060101.3    1018 GFPPDLTLSPRESIQLYDAMFQIWKSWPRAQELCPENFIHFNNKLVIKKMDARKYEESLK
```

```
XP_004940975.1 1187 KELSNWIAVGQRQKVNEVLENLKPQPVDCSEYEKWKRFARFVDKLHEMDKLPAIFFIFGL
XP_027312235.1 1197 KELSKWIELGQRQKVCELLDHFKPQPMDCSDQEKWMQFACFVEKLHEMDKLPAIIFIFGL
XP_013030722.1 1238 KELSKWIELGQRQKVYELLDHFKPQPVDCSDQEKWMQFACFVEKLHEMDKLPAIIFIFGL
NP_060101.3    1078 AELTSWIKNGNVEQARMVLQNLSPE-ADLSPENMITMFPLLVEKLRKMEKLPALFFLFKL

XP_004940975.1 1247 DSVENAAWNVFRNLLEKQDNERDPKMEKEKEDIKNKLRKLNKTLMKFEPTDIKKVKPSK-
XP_027312235.1 1257 DSVERAATNVFIHLMEKQKNEQDPKAKREIEDIKDKLRKVDKSLTKCKTNDTKKLKTSK-
XP_013030722.1 1298 DSVERAANNIFIHLMEKRKNEQDPKAKREIEDIKDKLRKVNKSLMKCKPNDTKKLKPSK-
NP_060101.3    1137 GAVENAAESVSTFLKKKQETKRPPKADKEAHVMANKLRKVKKSIEKQKIIDEKSQKKTRN

XP_004940975.1 1306 VENMVMLLTMKKQLQERLKKLTVIHSMCTYADPKAVDEDTLRKIFYRLRFQRRGYVHQTL
XP_027312235.1 1316 AESIIQLWTMKNELEKRLKKLSVTPSACTYADPKAVDEDTLRKIFYRLRFERKGYLQQML
XP_013030722.1 1357 MESIIQLLTMKKELQKRLEKLSVIPSACTYADPKAVDEDTLRKIFYRLRFERKGYLQEML
NP_060101.3    1197 VDQSLIHEAEHDNLVKCLEKNLEIPQDCTYADQKAVDTETLQKVFGRVKFERKGEELKAL
```

Figure S13: Schematic alignment of DDX60 protein sequences of chicken, duck, goose and human. The critical conserved residues of ATP binding and phosphorylation, lysine (K791) and tyrosine (Y793 and Y796) respectively are boxed in black

```
XP_015152165.1     1 M-----------------------------------------------------------
XP_027328665.1     1 MVPTRPRLCSMAATPPPFAQRHAVLQQRVRGAGPTLGRGHGCHRPAPRRPASRKGKRKRR
XP_013053947.1     1 ------------------------------------------------------------
NP_066363.1        1 MEERRPHL----------------------------------------------------

XP_015152165.1     2 -----------------------------------------DGAGHKPLFRQPRAAGRDH
XP_027328665.1    61 RRRPGRASGARGPAASGRFLPVLWGAGQPRRPAEEPAAGAMEGAGQGPGLRLPRAGRRGH
XP_013053947.1     1 ----------------------------------------------GPGLRLPRAGRRGH
NP_066363.1        9 -------------------------------------------------DARPRNSHTNH

XP_015152165.1    21 ---PDVAFIQRGRSGINNVSGQTNK-AVRQAGEDPQASNYTLPLGKREDSFAGTAKFFNQ
XP_027328665.1   121 AGRWDAGFIQQGRNRINNVSGQTHK-TVAQAGENPQASNYTLLLGQREERS---EYFFSQ
XP_013053947.1    15 ADHWDTGFIQRGRNRINSVSGQTHK-TVAQAGENPQTSNYTPLLGQREERS---ECFFSR
NP_066363.1       20 RGPVDGELPPRARNQANNPPANALRGGASHPGRHPRANNHPAAYWQREERFRAMGRNPHQ

XP_015152165.1    77 GRRNQGGPVTSGATGHGNEQ-NDGRGRRYQGGQAHERARGSWNVQENESRERRYQGGQAH
XP_027328665.1   177 GRRNQGGQTNSGATGHWNEQENEGRGKWYRGGQTHERARGPWNEQENEGRRRRYQGGQAY
XP_013053947.1    71 GRRNQGGQANSGATGHWNEQENEGRGKRYQGGQTHERARGPWNEQENEGRRRRHQGGQAY
NP_066363.1       80 GRRNQEGHASDEARDQRHDQENDTR--WRNGNQDCRNRRPPW------------------

XP_015152165.1   136 ERARGSQNVQENERKGKQHVSLGQAQGRARGLWNEQDDEVRGRRYQGGQGHERVRGPWNE
XP_027328665.1   237 DR--------------------------DRGLWNEQDNEGRVRRYQGGRACERAGGPWNM
XP_013053947.1   131 ER--------------------------DRGLWNEQDNEGRGRRYQGGQAYERARGPWNM
NP_066363.1      120 ----------------------------------------------------------S-

XP_015152165.1   196 QENEGKGRRYQGG-----QVQEDARGPRNREEIEGKGRRMRLAGSPHFNHNFTPGGTQP-
XP_027328665.1   271 QENEVRGRRYHSGQALSGQAQEDARGLRNGQENEGKGRSIQLRRSMHFYQKPTPGGAQLS
XP_013053947.1   165 QENEGRGKCYQGGQALSGQAQEDARGLRNGQENEGKGRRIQPGRNTHFYQKPTPGGAQL-
NP_066363.1      121 --------------------------------------------NDNFQQWRTPHQ-KP-
```

```
XP_015152165.1  250 -----SEQPQQVKKVGYKFLESLLQKDSSEVVITLASSSGFKELLSQTSMKPGFLQLICQ
XP_027328665.1  331 EQLQLSEQPQQVKKIGYKFLEGLLQKDPSEVVITLASSSGLKELLSQTAMKPSFVQLICQ
XP_013053947.1  224 -----SEQPQQVKKIGYKFLEGLLQKDPSEVVITLASSSGLKELLSQTAMKPSFLQLICQ
NP_066363.1     135 -----TEQPQQAKKLGYKFLESLLQKDPSEVVITLATSLGLKELLSHSSMKSNFLELICQ

XP_015152165.1  305 VLRKACGSRMDRQSVQQLLGMVKESNFLKICLPQYVSDMVTEAVPAVRHQYPEHIGNIIL
XP_027328665.1  391 VLRKACSSRMDRQSVQQLLGVVKESNFLKICLPQYVSDMVTEAVPAVRHQYPEHIGNIIL
XP_013053947.1  279 VLRKACSSRMDRQSVQQLLGVVKESNFLKICLPQYVSDMVTEAVPAVRHQYPEHIGNIIL
NP_066363.1     190 VLRKACSSKMDRQSVLHVLGIIKNSKFLKVCLPAYVVGMITEPIPDIRNQYPEHISNIIS

XP_015152165.1  365 LLQDLISIFPSSSVQKISMLLTVLPASINALRASGVQITEETEKNLSKVQMLVQHLQEKR
XP_027328665.1  451 LLQDLISIFPASSVQKISILLTVLPASINALRASGVDITEETEKNLNKVQMLVQHLQEKR
XP_013053947.1  339 LLQDLISIFPASSVQKISILLTVLPASINALRASGVDIMEETEKNLNKVQMLVQHLQEKR
NP_066363.1     250 LLQDLVSVFPASSVQETSMLVSLLPTSLNALRASGVDIEEETEKNLEKVQTIIEHLQEKR

XP_015152165.1  425 REGTLRADNYTLMQPQADGQEETYRTMTIYPTYDELHHDVKPFLRPNIVSGRYESTNIYL
XP_027328665.1  511 REGTLRADNYTLMQPQADGQEETYRTMTIYPTYNEVHHDEKPFLRPNIVSGRYESTSIYL
XP_013053947.1  399 REGTLRADNYTLMQLQADGQEETYRTMTIYPTYNEVHHDEKPFLRPNIVSGRYESTSIYL
NP_066363.1     310 REGTLRVDTYTLVQPEAEDHVESYRTMPIYPTYNEVHLDERPFLRPNIISGKYDSTAIYL

XP_015152165.1  485 DTHFRLLREDFIKPLREGILDLLQSFEDKSLRKKKFDDIRIYFDTRIITPLCSPSGVVYK
XP_027328665.1  571 DTHFRLLREDFVRPLREGISELLQSFEDKGLRKKKFDDIRIYFDTRIIAPLCSPTGVVYK
XP_013053947.1  459 DTHFRLLREDFVRPLREGISELLQSFEDKGLRKKKFDDIRIYFDTRIIAPLCSPTGVVYK
NP_066363.1     370 DTHFRLLREDFVRPLREGILELLQSFEDQGLRKRKFDDIRIYFDTRIITPMCSSSGIVYK

XP_015152165.1  545 VQFDIKPLKFVRWQNSRRLLYGSLVCMSRDHFETCLFATVSERDNAELANGIVQLSFNAQ
XP_027328665.1  631 VQFDTKPLKFVRWQNSRRLLYGSLVCMSKDHFETCLFATVSNRDSTDLAKGIVELCFNAQ
XP_013053947.1  519 VQFDTKPLKFVRWQNSRRLLYGSLVCMSKDHFETCLFATVSNRDSTDLAKGIVQLCFNAQ
NP_066363.1     430 VQFDTKPLKFVRWQNSKRLLYGSLVCMSKDNFETFLFATVSNREQEDLCRGIVQLCFNEQ
```

```
XP_015152165.1  605 SRALLAEVQPSDSFLMVETTAYFEAYRHVLEGLQEIQEEDIPFQKYIVECDPQLKEPAYL
XP_027328665.1  691 SQALLAEVRPSDSFLMVETTAYFEAYRHVLEGLQEIQEEDIPFQKYIVECDAQVKEPKYL
XP_013053947.1  579 SQALLAEVRPSDSFLMVETTAYFEAYRHVLEGLQEIQEEDIPFQKYIVECDAQVKEPTYL
NP_066363.1     490 SQQLLAEVQPSDSFLMVETTAYFEAYRHVLEGLQEVQEEDVPFQRNIVECNSHVKEPRYL

XP_015152165.1  665 KMDTKYNFAPLTEDTLSDEE--TYLDGLRRQNVRVLDPNQWPSMETLGLDESQMQALSLA
XP_027328665.1  751 TVDTTYNFAPLTEDPLADEE--RAPDGLRRQSVRVLDHNQWPSMETLGLDESQMEALRLA
XP_013053947.1  639 TVDTTYNFAPLTEDPLADEE--RAPDGLRRQSFHVLDPNRWPSMETLGLDESQMEALRLA
NP_066363.1     550 LMGGRYDFTPLIENPSATGEFLRNVEGLRHPRINVLDPGQWPSKEALKLDDSQMEALQFA

XP_015152165.1  723 LTKELAIIQGPPGTGKTYVGLKIVQALLTNQDAWQRTDQNSPILVVCYTNHALDQFLEGI
XP_027328665.1  809 LTKELAIIQGPPGTGKTYVGLKIVQALLTNKNVWQTTVQNSPILVVCYTNHALDQFLEGI
XP_013053947.1  697 LTKELAIIQGPPGTGKTYVGLKIVQALLTNKHVWQTTVQNSPILVVCYTNHALDQFLEGI
NP_066363.1     610 LTRELAIIQGPPGTGKTYVGLKIVQALLTNESVWQISLQKFPILVVCYTNHALDQFLEGI

XP_015152165.1  783 YTFQKRGIVRVGGRSNSEILKQFTLKELRKKSEFRHNLPMHLRRAYVNITCDMKHAEEEL
XP_027328665.1  869 YTFQKHGMVRVGGRSSSEVLKQFTLRELRKKCEFRHNLPMHLRRAYVNITSQMKQAEEEL
XP_013053947.1  757 YTFQKHGMVRVGGRSSSEVLKQFTLRELRKKCEFRHNLPMHLRRAYVNITSQMKQAEEEL
NP_066363.1     670 YNCQKTSIVRVGGRSNSEILKQFTLRELRNKREFRRNLPMHLRRAYMSIMTQMKESEQEL

XP_015152165.1  843 YKGAKHLECTTYGVLHERHLEAFIAPQHWDSLMSGL--DDDEFYYSASRHSMILEWLGLG
XP_027328665.1  929 HKGAKHLECTTHGVLHERHLEACIAPQHWDSLMKGL--DHEEFYYSAAQHSVILEWLGLG
XP_013053947.1  817 HKGAKHLECTTYGVLHERHLEACIAPQHWDSLMKGL--DHEEFYYSAAQHSVILEWLGLG
NP_066363.1     730 HEGAKTLECTMRGVLREQYLQKYISPQHWESLMNGPVQDSEWICFQHWKHSMMLEWLGLG

XP_015152165.1  901 FTAFTQSAARNIEAENLGDQQEEREEEQEGEAEEDLLEILEEADLIQADRVIDDEDSAKA
XP_027328665.1  987 VTVFTQSAAEDIEAENPGGQQ-EREEEQEGEAEE-LLEIPEEADLIQADRVIEDEEAAKP
XP_013053947.1  875 VTVFTQSAAEDIEAENPGGQQ-EREEEQEGEAEE-LLEIPEEADLIQADRVIEDEEAAKP
NP_066363.1     790 VGSFTQSVSPA-GPENT-AQA-EGDEEEEGEEESSLIEIAEEADLIQADRVIEEEEVVRP
```

```
XP_015152165.1  961 QRRKEEEHRAVHELAGMLLAMKLENEEEETVQPQ-QKSAQWEITSSQRKKMKHKMKVELH
XP_027328665.1 1045 QKRKEEEHRAVRELASVLLAMKLENEEEGTAQPQ-QKTVQWEITATQRKKMKQKMRVELC
XP_013053947.1  933 QKRKEEEHRAVRELASVLLAMKLENEEEGTAQPQ-QKTVQWEITASQRKKMKQKMRVELR
NP_066363.1     847 QRRKKEESGADQELAKMLLAMRLDHCGTGTAAGQEQATGEWQTQRNQKKKMKKRVKDELR

XP_015152165.1 1020 KLNAMSETEAYAIQDLWQLDMNSRWRLYRLWLQTYQGLIRRKILQHEQQYQAAAERLAEL
XP_027328665.1 1104 KLSAMTELEAKSIQDLWQLDLSSRWRLYRLWLQTYQGFIRRRILQHEQQYQAAAERLAEL
XP_013053947.1  992 KLSAMTELEAKSIQDLWQLDLSSRWRLYRLWLQTYQGFIRRRILQHEQQYQAAAERLAEL
NP_066363.1     907 KLNTMTAAEANEIEDVWQLDLSSRWQLYRLWLQLYQADTRRKILSYERQYRTSAERMAEL

XP_015152165.1 1080 RLQEDLCILKEAKVVGMTTTGAAKYRQILQKVEPRIVIVEEAAEVLEAHTITTLSKACQH
XP_027328665.1 1164 RLQEDLCILKEAKVVGMTTTGAAKYRQILQKVEPRIVIVEEAAEVLEAHTITTLSKACQH
XP_013053947.1 1052 RLQEDLCILKEAKVVGMTTTGAAKYRQILQKVEPRIVIVEEAAEVLEAHTITTLSKACQH
NP_066363.1     967 RLQEDLHILKDAQVVGMTTTGAAKYRQILQKVEPRIVIVEEAAEVLEAHTIATLSKACQH

XP_015152165.1 1140 LILIGDHQQLQPSANVYDLAKNFNLEVSLFERLVKVDFPFVCLKYQHRMRPEIAQLLSPH
XP_027328665.1 1224 LILIGDHQQLQPSANVYDLAKNFNLEVSLFERLVKVDFPFVRLKYQHRMRPEIAQLLSPH
XP_013053947.1 1112 LILIGDHQQLQPSANVYDLAKNFNLEVSLFERLVKVNFPFVRLKYQHRMRPEIAQLLSPH
NP_066363.1    1027 LILIGDHQQLRPSANVYDLAKNFNLEVSLFERLVKVNIPFVRLNYQHRMCPEIARLLTPH

XP_015152165.1 1200 IYQDLENHPSVLKYENVKGVLSNLFFVEHDFPEQVIQEGRSHQNIHEAQFVVELCKYFLC
XP_027328665.1 1284 IYQELENHPSVLKYENIKGVLSNLFFVEHDFPEQEIQEGKSHQNPHEARFVVELCKYFLC
XP_013053947.1 1172 IYQELENHPSVLKYENIKGVLSNLFFVEHDFPEQEIQEGKSHQNPHEAQFVVELCKYFLC
NP_066363.1    1087 IYQDLENHPSVLKYEKIKGVSSNLFFVEHNFPEQEIQEGKSHQNQHEAHFVVELCKYFLC

XP_015152165.1 1260 QDYQPSQITILTTYTGQLFCLRKLMPAKTFAGVKVYVVDKYQGEENDIILLSLVRSNKEE
XP_027328665.1 1344 QDYLPSQITILTTYTGQLFCLRKLMPAKTFAGVKVYVVDKYQGEENDIILLSLVRSNKEE
XP_013053947.1 1232 QDYLPSQITILTTYTGQLFCLRKLMPAKTFAGVKVYVVDKYQGEENDIILLSLVRSNKEE
NP_066363.1    1147 QEYLPSQITILTTYTGQLFCLRKLMPAKTFAGVRVHVVDKYQGEENDIILLSLVRSNQEG
```

```
XP_015152165.1 1320 RIGFLQIPNRICVALSRAKKGLYCIGNMRMLGKVPLWSKIIHTLREKGNIGRSLTLCCQN
XP_027328665.1 1404 RTGFLQIPNRICVALSRAKKGLYCIGNMRMLGKVPLWSKIIHTLREKGNIGRSLILCCQN
XP_013053947.1 1292 RTGFLQIPNRICVALSRAKKGLYCIGNMRMLGKVPLWSKIIHTLREKGNIGRSLMLCCQN
NP_066363.1    1207 KVGFLQISNRICVALSRAKKGMYCIGNMQMLAKVPLWSKIIHTLRENNQIGPMLRLCCQN

XP_015152165.1 1380 HPETITSVSTAADFSKVPEGGCSHPCEFRLDCGHVCTRACHPYDLQHKKYQCLKPCQKVL
XP_027328665.1 1464 HPETKTLVSTAADFSKVPEGGCSHPCEFRLSCGHVCTRACHPYDTQHKKYQCLKPCQKVL
XP_013053947.1 1352 HPETKTLVSTAADFSKVPEGGCSHPCEFRLSCGHVCTRACHPYDAQHKKYQCLKPCQKVL
NP_066363.1    1267 HPETHTLVSKASDFQKVPEGGCSLPCEFRLGCGHVCTRACHPYDSSHKEFQCMKPCQKVI

XP_015152165.1 1440 CTEGHRCPLLCYEPCGPCMVIVEKIVPKCGHLQMIPCSCPGSKFICQEPCKKKLNCGHTC
XP_027328665.1 1524 CAKGHRCQQSCYEPCGPCMVIVEKTISKCGHLQMVPCSCPDSEFVCQEPCQKKLNCGHTC
XP_013053947.1 1412 CTKGHRCLQSCYEPCGPCMVIVEKTISKCGHLQMVPCSCPDSEFVCQEPCQKKLNCGHTC
NP_066363.1    1327 CQEGHRCPLVCFQECQPCQVKVPKTIPRCGHEQMVPCSVPESDFCCQEPCSKSLRCGHRC

XP_015152165.1 1500 NKCCGQECTTRCPELVTVFLQCGHRQKVQCWITDATKHQ-KSVECMAKCSVKLECGHECS
XP_027328665.1 1584 NRFCGQECTTRCPELVKVTLQCGHTQKVKCWITEEMKHG-KPVECKTKCSVTLECGHACS
XP_013053947.1 1472 NRFCGQECTARCPELVEVTLQCGHSQQVKCWITEEMKHG-KPVECRTKCSVTLECGHVCS
NP_066363.1    1387 SHPCGEDCVQLCSEMVTIKLKCGHSQPVKCGHVEGLLYGGLLVKCTTKCGTILDCGHPCP

XP_015152165.1 1559 GSCHTCFGGRFHKPCKSPCKRYLICSHKCQQPCTTECPPCQLDCQNHCVHSRCKKKCGER
XP_027328665.1 1643 GSCHTCFEGRFHKPCSSPCKRFLVCSHKCQQPCTVECPPCQLDCQNHCVHSRCKKKCGER
XP_013053947.1 1531 GSCHTCFEGRFHKPCSSPCKRFLVCSHKCRQPCTVECPPCQLDCQNHCVHSRCKKKCGER
NP_066363.1    1447 GSCHSCFEGRFHERCQQPCKRLLICSHKCQEPCIGECPPCQRTCQNRCVHSQCKKKCGEL

XP_015152165.1 1619 CSPCAEPCEWRCKHYQCTNLCHEPCNRPRCNIPCTKLLRCGHPCIGFCGEPCPKKCLVCD
XP_027328665.1 1703 CFPCVEPCEWQCQHYQCTNLCSEPCNRPRCDVPCTKLLRCGHPCIGLCGEPCPNKCLVCD
XP_013053947.1 1591 CFPCVEPCEWRCQHYQCTNLCSEPCNRPRCNVPCTKLLRCGHPCIGLCGEPCPKKCLVCD
NP_066363.1    1507 CSPCVEPCVWRCQHYQCTKLCSEPCNRPPCYVPCTKLLVCGHPCIGLCGEPCPKKCRICH
```

```
XP_015152165.1 1679 REEVTQIFFGFEDDEDARFVQLEDCGHIFESQGLDRYMDED--DD-VIKLKVCPTCQTPV
XP_027328665.1 1763 HEEVTQIFFGFEEDPDARFVQLEDCGHVFESQGLDHYMDED--DD-VVKLKVCPVCQTPV
XP_013053947.1 1651 HEEVTQIFFGFEEDPDARFVQLEDCGHVFESQGLDHYMDED--DD-VIKLKVCPVCQTPV
NP_066363.1    1567 MDEVTQIFFGFEDEPDARFVQLEDCSHIFEVQALDRYMNEQKDDEVAIRLKVCPICQVPI

XP_015152165.1 1736 RKSLRYGTIVKKHLVEIEKVKEKIQGPAREIESSRRRLQAALTSKSVLKRNLLTKYLMLE
XP_027328665.1 1820 RKNLRYGTIVKRRLDEIEKVKEKIQGPAQEIESSRQRLQAALIGNAVLQRNLPHKYLMLE
XP_013053947.1 1708 RKNLRYGTIVKRRLDEIEKVKEKIQGPAQEIESSRRRLQAALIGNAVLQRNLPHKYLMLE
NP_066363.1    1627 RKNLRYGTSIKQRLEEIEIIKEKIQGSAGEIATSQERLKALLERKSLLHQLLPEDFLMLK

XP_015152165.1 1796 DQLNASDLSIKSIGVIENQLNFYERIADLTSSLSKIDEKEQKGMKKRLDEVQEWLDRPRL
XP_027328665.1 1880 DKLKASDLSIKSIGLIENQLNFYKRVADLTDSMSKIDVNERKGLKKRLDEVQEWLDMPRI
XP_013053947.1 1768 DKLKASDLSIKSIGLIENQLNFYKRVADLTDSMSKIDVNERKGLKKRLDEVQEWLDLPRI
NP_066363.1    1687 EKLAQKNLSVKDLGLVENYISFYDHLASLWDSLKKMHVLEEKRVRTRLEQVHEWLAKKRL

XP_015152165.1 1856 SFTRQELMDLQAEIQRLTYLQHLLSRCKGRSGMITTTLAAKIATVREILEGTKRFTEKEE
XP_027328665.1 1940 SFTGQELSDLQAEIQRLTLLQSLLSRCKSASGMITTALAAEIACVREILEGTKKFTEADE
XP_013053947.1 1828 SFTGQELSDLQAEIQRLTFLQSLLSRCKSASGMITAALAAEIASVREILEGTKKFTKADE
NP_066363.1    1747 SFTSQELSDLRSEIQRLTYLVNLLTRYKIAEKKVKDSIAVEVYSVQNILEKTCKFTQEDE

XP_015152165.1 1916 AAVKAELKHISAALPLSGLGITEAERVQIVSAIGCPRGHWFKCKNGHVYVIGDCGGAMER
XP_027328665.1 2000 AAVKAQLERLCTALPVSGLGISEAERVQIVRAIGCPRGHWFKCRNGHIYVIGECGGAMER
XP_013053947.1 1888 AAVKAELERLCTALPVSGLGISEAERVQIVRAIGCPRGHWFKCRNGHIYVIGECGGAMER
NP_066363.1    1807 QLVQEKMEALKATLPCSGLGISEEERVQIVSAIGYPRGHWFKCRNGHIYVIGDCGGAMER

XP_015152165.1 1976 SRCPECHEVIGGANHALDSTNSLAPEMDGATHAAWSETANNLLNFQDLQRLL
XP_027328665.1 2060 SRCPECHAVIGGTNHALDSTNSLAPEMDGATHAAWSETANNLLNFEELRLLL
XP_013053947.1 1948 SKCPECHAVIGGTNHTLDSTNSLAPEMDGATHAAWSEAANNLLNFEELRRLL
NP_066363.1    1867 GTCPDCKEVIGGTNHTLERSNQLASEMDGAQHAAWSDTANNLMNFEEIQGMM
```

Figure S14: The protein sequences of chicken, mallard, goose and human ZNFX1 aligned using the ClustalW program. The armadillo, P-loop and zinc finger domains are indicated below the sequence in black line, blue line, and red line respectively.

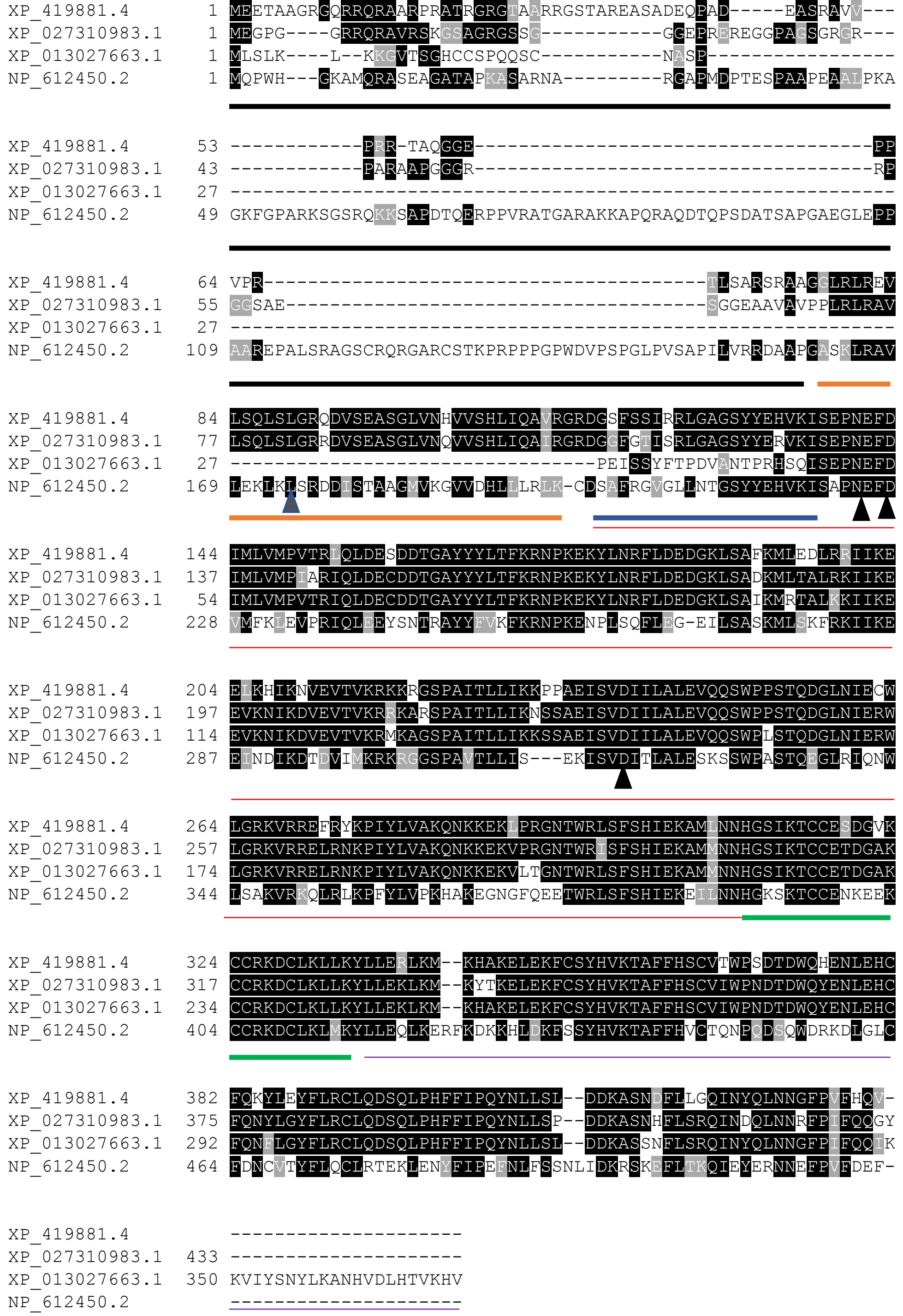
XP_419881.4 1 MEETAAGRGQRRQRAARPRATRGRGTAARRGSTAREASADEQPAD-----EASRAVV---
XP_027310983.1 1 MEGPG----GRRQRAVRSKGSAGRGSSG-----------GGEPREREGGPAGSGRGR---
XP_013027663.1 1 MLSLK----L--KKGVTSGHCCSPQQSC-----------NASP-----------------
NP_612450.2 1 MQPWH---GKAMQRASEAGATAPKASARNA---------RGAPMDPTESPAAPEAALPKA
XP_419881.4 53 ------------PRR-TAQGGE------------------------------------PP
XP_027310983.1 43 ------------PARAAPGGGR------------------------------------RP
XP_013027663.1 27 --
NP_612450.2 49 GKFGPARKSGSRQKKSAPDTQERPPVRATGARAKKAPQRAQDTQPSDATSAPGAEGLEPP
XP_419881.4 64 VPR--TLSARSRAAGGLRLREV
XP_027310983.1 55 GGSAE--------------------------------------SGGEAAVAVPPLRLRAV
XP_013027663.1 27 --
NP_612450.2 109 AAREPALSRAGSCRQRGARCSTKPRPPPGPWDVPSPGLPVSAPILVRRDAAPGASKLRAV
XP_419881.4 84 LSQLSLGRQDVSEASGLVNHVVSHLIQAVRGRDGSFSSIRRLGAGSYYEHVKISEPNEFD
XP_027310983.1 77 LSQLSLGRRDVSEASGLVNQVVSHLIQAIRGRDGGFGTISRLGAGSYYERVKISEPNEFD
XP_013027663.1 27 ---------------------------------PEISSYFTPDVANTPRHSQISEPNEFD
NP_612450.2 169 LEKLKLSRDDISTAAGMVKGVVDHLLLRLK-CDSAFRGVGLLNTGSYYEHVKISAPNEFD
XP_419881.4 144 IMLVMPVTRLQLDESDDTGAYYYLTFKRNPKEKYLNRFLDEDGKLSAFKMLEDLRRIIKE
XP_027310983.1 137 IMLVMPIARIQLDECDDTGAYYYLTFKRNPKEKYLNRFLDEDGKLSADKMLTALRKIIKE
XP_013027663.1 54 IMLVMPVTRIQLDECDDTGAYYYLTFKRNPKEKYLNRFLDEDGKLSAIKMRTALKKIIKE
NP_612450.2 228 VMFKLEVPRIQLEEYSNTRAYYFVKFKRNPKENPLSQFLEG-EILSASKMLSKFRKIIKE
XP_419881.4 204 ELKHIKNVEVTVKRKKRGSPAITLLIKKPPAEISVDIILALEVQQSWPPSTQDGLNIECW
XP_027310983.1 197 EVKNIKDVEVTVKRRKARSPAITLLIKNSSAEISVDIILALEVQQSWPPSTQDGLNIERW
XP_013027663.1 114 EVKNIKDVEVTVKRMKAGSPAITLLIKKSSAEISVDIILALEVQQSWPLSTQDGLNIERW
NP_612450.2 287 EINDIKDTDVIMKRKRGGSPAVTLLIS---EKISVDITLALESKSSWPASTQEGLRIQNW
XP_419881.4 264 LGRKVRREFRYKPIYLVAKQNKKEKLPRGNTWRLSFSHIEKAMLNNHGSIKTCCESDGVK
XP_027310983.1 257 LGRKVRRELRNKPIYLVAKQNKKEKVPRGNTWRISFSHIEKAMMNNHGSIKTCCETDGAK
XP_013027663.1 174 LGRKVRRELRNKPIYLVAKQNKKEKVLTGNTWRLSFSHIEKAMMNNHGSIKTCCETDGAK
NP_612450.2 344 LSAKVRKQLRLKPFYLVPKHAKEGNGFQEETWRLSFSHIEKEILNNHGKSKTCCENKEEK
XP_419881.4 324 CCRKDCLKLLKYLLERLKM--KHAKELEKFCSYHVKTAFFHSCVTWPSDTDWQHENLEHC
XP_027310983.1 317 CCRKDCLKLLKYLLEKLKM--KYTKELEKFCSYHVKTAFFHSCVIWPNDTDWQYENLEHC
XP_013027663.1 234 CCRKDCLKLLKYLLEKLKM--KHAKELEKFCSYHVKTAFFHSCVIWPNDTDWQYENLEHC
NP_612450.2 404 CCRKDCLKLMKYLLEQLKERFKDKKHLDKFSSYHVKTAFFHVCTQNPQDSQWDRKDLGLC
XP_419881.4 382 FQKYLEYFLRCLQDSQLPHFFIPQYNLLSL--DDKASNDFLLGQINYQLNNGFPVFHQV-
XP_027310983.1 375 FQNYLGYFLRCLQDSQLPHFFIPQYNLLSP--DDKASNHFLSRQINDQLNNRFPIFQQGY
XP_013027663.1 292 FQNFLGYFLRCLQDSQLPHFFIPQYNLLSL--DDKASSNFLSRQINYQLNNGFPIFQQIK
NP_612450.2 464 FDNCVTYFLQCLRTEKLENYFIPEFNLFSSNLIDKRSKEFLTKQIEYERNNEFPVFDEF-
XP_419881.4 ---------------------
XP_027310983.1 433 ---------------------
XP_013027663.1 350 KVIYSNYLKANHVDLHTVKHV
NP_612450.2 ---------------------

Figure S15: The protein sequences of chcGAS, ducGAS, gocGAS, hucGAS aligned using the ClustalW program. Conserved amino acids and synonymous substitutions are shadowed in black and gray, respectively. The N-terminal tail, spine, activation loop, zinc finger and conserved catalytic residues are indicated below the sequence in black line, orange line, blue line, green line and black triangles respectively. The red and purple lines underneath indicate α/β core and helix bundle respectively. Catalytic residues are highlighted with black triangles. Conserved leucine involved in dsDNA binding is highlighted with grey triangle.

```
XP_025010645.1     1 MPQDPST-----------------------------------------------------
XP_027323921.1     1 MSQEPQR-----------------------------------------------------
XP_013057484.1     1 MSQPAASPCAAQGRIPAGQGTSWKFSVGGLPRLHPLPAPAIAPFIFTSLLIGFSGTMRLC
NP_938023.1        1 MPHSSLH-----------------------------------------------------

XP_025010645.1     8 ---RSSPARLLIPEPRAGRARHAACVLLAVCFVVLFLSGEPLAPIIRSVCTQLAALQLGV
XP_027323921.1     8 ---RSSPAALLIPKPREGRAQRAVYVLLATCTVALCLAGEPLSPAAHRVSAHLAALQIGA
XP_013057484.1    61 PRHREQAQALLAAAASEGRRKRCLSGLGA-------------AHSARSARSPGSSQCRGR
NP_938023.1        8 ---------PSIPCPRGHGAQKAALVLLSACLVTLWGLGEPPEHTLRYLVLHLASLQLGL

XP_025010645.1    65 LL---------KGCCCLAEEIFHLHSRHHGSL-----------------WQ-VLCSCFP-
XP_027323921.1    65 LL---------KGCCCLAEEIFHLQSRYRGSF-----------------WR-ALSACFP-
XP_013057484.1   108 GRSDVSGTAAPEQPHCL----AHPQGQGRASAARGVRPPGHLHRGAVPGWGAPVTRCPP-
NP_938023.1       59 LL---------NGVCSLAEELRHIHSRYRGSY-----------------WR-TVRACLGC

XP_025010645.1    97 PRWYL---------ALLLVGGSAYLDPPEDNGHSPRLALTLSCLCQLLVLALGLQKLSAV
XP_027323921.1    97 PRWHL---------ALLLVGGSAYLDLQQGERLSP--HLALTCLCHLLALALGLQKPSAV
XP_013057484.1   163 PHRPPGSPADRDLAQGLLLPGRGDLP-------------------------------PPV
NP_938023.1       92 PLRRG---------ALLLLSIYFYYSLPNAVGPPFTWMLALLGLSQALNILLGLKGLAPA

XP_025010645.1   148 EVS-ELTESSKKNVAHGLAWSYYI----G-----------------------YLKVVLPR
XP_027323921.1   146 EIS-EMTERSQQNVAHGLAWSYYV----G-----------------------YLKIVLPR
XP_013057484.1   192 QIPRQLLEGAERVLPTTLAPGPAVHRWLSLPGPAAGRQAQPLPCPDLPVPSPRPRPRAPG
NP_938023.1      143 EIS-AVCEKGNFNVAHGLAWSYYI----G-----------------------YLRLILPE

XP_025010645.1   180 LKECMEELSRTNPMLRAHRDTWKLHILVPLGCDIWDDLEKADSNIQYLADLPETILTRAG
XP_027323921.1   178 IKECMEEISRANLNVWACRETWKLHILIPLSCNVWDDLEKADSNIQYLTDLTETTLTRGG
XP_013057484.1   252 IKESMEEISKANPNVQACRETWKLHILIPLSCNVWDDLEKADSNIQYLTDLTETTLTRGG
NP_938023.1      175 LQARIRTYNQHYNNLLRGAVSQRLYILLPLDCGVPDNLSMADPNIRFLDKLPQQTGDHAG

XP_025010645.1   240 IKRRVYKHSLYVIRDKDNKLRPCVLEFASPLQTLCAMSQDDCAAFSREQRLEQARLFYRS
XP_027323921.1   238 IKKRVYKHSLYAIRDGDNQARHCAVEFATPLQTLYAMSQDECAAFSRDDRLEQAKLFYRT
XP_013057484.1   312 IKKRVYKHSFYAIRDGDNQARHCAVEYATPLQTLYAMSQDECAAFSRDDRLEQAKLFYRT
NP_938023.1      235 IKDRVYSNSIYELLENGQRAGTCVLEYATPLQTLFAMSQYSQAGFSREDRLEQAKLFCRT

XP_025010645.1   300 LRDILGSSKECAGLYRLIAYEEPAEPESHFLSGLILWHLQQQQREEYMVQEELPL-----
XP_027323921.1   298 LEEILRGSKECAGTYRLIAYEELVEAEPHFLSREILWHLRQQQQEEFTVYEGSPPRTLAT
XP_013057484.1   372 LEEILRGSKECTGTYRLIAYEEPREAEPHFLSREILWHLRQQQQEEFTVYEGTPPRTLAT
NP_938023.1      295 LEDILADAPESQNNCRLIAYQEPADDSSFSLSQEVLRHLRQEEKEEVTVGSLKTSAVPST

XP_025010645.1   355 GTSSVELSLQVSSSDLPQPLRSDCP
XP_027323921.1   358 ALGSTDLSLQISASDLPQPLRSDHP
XP_013057484.1   432 ALGSTDLSLQISASDLPQPLRSDHP
NP_938023.1      355 STMSQEPELLISGMEKPLPLRTDFS
```

Figure S16: The protein sequences of chSTING, duSTING, goSTING and huSTING aligned using the ClustalW program. Conserved amino acids and synonymous substitutions are shadowed in black and gray, respectively. The TM domains 1, 2, 3, and 4 are indicated below the sequence in black lines. The cyclic dinucleotide binding domain and C-terminal tail are underscored by orange and grey lines respectively. Residues involved in cyclic dinucleotide binding are highlighted using black triangles. pLxIS motif is underscored in blue

```
NP_001185786.1    1 MNSLEQAEDLKAFERRLTEYIACLQPATGRWRMILIVVSVCTATGAWNWLIDPETQKVSF
XP_027323010.1    1 MNSLEQAEDLKAFERRLTEYIACLQPATGRWRTILIVVSVCTATGAWNWLIDPETQKVSF
XP_013042385.1    1 MI----IADLKAFERRLTEYIACLQPATGRWRMILIVVSVCTATGAWNWLIDPETQKVSF
NP_694993.2       1 MNSLEQAEDLKAFERRLTEYIHCLQPATGRWRMLLIVVSVCTATGAWNWLIDPETQKVSF

NP_001185786.1   61 FTSLWNHPFFTISCITLIGLFFAGIHKRVVAPSIIAARCRTVLAEYNMSCDDTGKLILKP
XP_027323010.1   61 FTSLWNHPFFTISCITLIGLFFAGIHKRVVAPSIIAARCRTVLAEYNMSCDDTGKLILKP
XP_013042385.1   57 FTSLWNHPFFTISCITLIGLFFAGIHKRVVAPSIIAARCRTVLAEYNMSCDDTGKLILKP
NP_694993.2      61 FTSLWNHPFFTISCITLIGLFFAGIHKRVVAPSIIAARCRTVLAEYNMSCDDTGKLILKP

NP_001185786.1  121 RPHVQ
XP_027323010.1  121 RPHVQ
XP_013042385.1  117 RPHVQ
NP_694993.2     121 RPHVQ
```

Figure S17: The ERADp protein sequences of chicken, duck, goose and human aligned using the ClustalW program. Conserved amino acids and synonymous substitutions are shadowed in black and gray, respectively. The transmembrane (TM) domains 1 and 2 are indicated below the sequence in black lines. Sequences preceding TM1 and after TM2 comprise N- and C-terminal regions respectively.

```
NP_001336637.1  355 TIFSYFKGQRQTLLFSATMPKKIQNFAKSALVKPITINVGRAGAASLDVVQEVEYVKEEA
XP_027324165.1  356 TIFSYFKGQRQTLLFSATMPKKIQNFAKSALVKPITINVGRAGAASLDVVQEVEYVKEEA
XP_013047316.1  234 TIFSYFKGQRQTLLFSATMPKKIQNFAKSALVKPITINVGRAGAASLDVVQEVEYVKEEA
NP_057306.2     360 TIFSYFKGQRQTLLFSATMPKKIQNFAKSALVKPVTINVGRAGAASLDVIQEVEYVKEEA

NP_001336637.1  415 KMVYLLECLQKTPPPVLIFAEKKADVDAIHEYLLLKGVEAVAIHGGKDQEERTKAIEAFR
XP_027324165.1  416 KMVYLLECLQKTPPPVLIFAEKKADVDAIHEYLLLKGVEAVAIHGGKDQEERTKAIEAFR
XP_013047316.1  294 KMVYLLECLQKTPPPVLIFAEKKADVDAIHEYLLLKGVEAVAIHGGKDQEERTKAIEAFR
NP_057306.2     420 KMVYLLECLQKTPPPVLIFAEKKADVDAIHEYLLLKGVEAVAIHGGKDQEERTKAIEAFR

NP_001336637.1  475 DGKKDVLVATDVASKGLDFPAIQHVINYDMPEEIENYVHRIGRTGRSGNTGIATTFINKA
XP_027324165.1  476 DGKKDVLVATDVASKGLDFPAIQHVINYDMPEEIENYVHRIGRTGRSGNTGIATTFINKA
XP_013047316.1  354 DGKKDVLVATDVASKGLDFPAIQHVINYDMPEEIENYVHRIGRTGRSGNTGIATTFINKA
NP_057306.2     480 EGKKDVLVATDVASKGLDFPAIQHVINYDMPEEIENYVHRIGRTGRSGNTGIATTFINKA

NP_001336637.1  535 CDESVLMDLKALLLEAKQKVPPVLQVLHCGDETMLDIGGERGCAFCGGLGHRITDCPKLE
XP_027324165.1  536 CDESVLMDLKALLLEAKQKVPPVLQVLHCGDETMLEIGGERGCAFCGGLGHRITDCPKLE
XP_013047316.1  414 CDESVLMDLKALLLEAKQKVPPVLQVLHCGDETMLEIGGERGCAFCGGLGHRITDCPKLE
NP_057306.2     540 CDESVLMDLKALLLEAKQKVPPVLQVLHCGDESMLDIGGERGCAFCGGLGHRITDCPKLE

NP_001336637.1  595 AMQTKQVSNIGRKDYLAHSSMDF
XP_027324165.1  596 AMQTKQVSNIGRKDYLAHSSMDF
XP_013047316.1  474 AMQTKQVSNIGRKDYLAHSSMDF
NP_057306.2     600 AMQTKQVSNIGRKDYLAHSSMDF
```

Figure S18: Schematic alignment of DDX41 protein sequences of chicken, duck, goose and human. Depicted are DEAD box region, HelicC and zinc finger domains in black, red and green underlining respectively. The DEAD box is boxed in blue.

```
XP_025002966.1    1 MPRG----DRESDWREKEQFRKLFIGGLSFETTEESLRNYYEQWGKLTDCVVMRDPASKR
XP_021129883.1    1 MET--------SKEREKEQFRKLFIGGLSFETTEESLRNYYEQWGKLTDCVVMRDPASKR
XP_013027335.1    1 MET--------SKEREKEQFRKLFIGGLSFETTEESLRNYYEQWGKLTDCVVMRDPASKR
NP_112533.1       1 MEKTLETVPLERKKREKEQFRKLFIGGLSFETTEESLRNYYEQWGKLTDCVVMRDPASKR

XP_025002966.1   57 SRGFGFVTFSSMAEVDAAMAARPHTIDGRVVEPKRAVAREESGKPGAHVTVKKLFVGGIK
XP_021129883.1   53 SRGFGFVTFSSMAEVDAAMAARPHTIDGRVVEPKRAVAREESGKPGAHVTVKKLFVGGIK
XP_013027335.1   53 SRGFGFVTFSSMAEVDAAMAARPHTIDGRVVEPKRAVAREESGKPGAHVTVKKLFVGGIK
NP_112533.1      61 SRGFGFVTFSSMAEVDAAMAARPHSIDGRVVEPKRAVAREESGKPGAHVTVKKLFVGGIK

XP_025002966.1  117 EDTEEHHLRDYFEEYGKIDTIEIITDRQSGKKRGFGFVTFDDHDPVDKIVLQKYHTINGH
XP_021129883.1  113 EDTEEHHLRDYFEEYGKIDTIEIITDRQSGKKRGFGFVTFDDHDPVDKIVLQKYHTINGH
XP_013027335.1  113 EDTEEHHLRDYFEEYGKIDTIEIITDRQSGKKRGFGFVTFDDHDPVDKIVLQKYHTINGH
NP_112533.1     121 EDTEEHHLRDYFEEYGKIDTIEIITDRQSGKKRGFGFVTFDDHDPVDKIVLQKYHTINGH

XP_025002966.1  177 NAEVRKALSRQEMQEVQNSRSGRGGNFGFGDARGGGGNFGPGPGSNFRGGAGKTDGYGSG
XP_021129883.1  173 NAEVRKALSRQEMQEVQNSRSGRGGNFGFGDARGGGGNFGPGPGSNFRGGAGKTDGYGSG
XP_013027335.1  173 NAEVRKALSRQEMQEVQNSRSGRGGNFGFGDARGGGGNFGPGPGSNFRGGAGKTDGYGSG
NP_112533.1     181 NAEVRKALSRQEMQEVQSSRSGRGGNFGFGDSRGGGGNFGPGPGSNFRGG---SDGYGSG

XP_025002966.1  237 RGFGDGYNGYGGGPGGGNFGGSPGYGGGRGGYGGGGPGYGNQGGGYGGGYDNYGGGNYGS
XP_021129883.1  233 RGFGDGYNGYGGGPGGGNFGGSPGYGGGRGGYGGGGPGYGNQGGGYGGGYDNYGGGNYGS
XP_013027335.1  233 RGFGDGYNGYGGGPGGGNFGGSPGYGGGRGGYGGGGPGYGNQGGGYGGGYDNYGGGNYGS
NP_112533.1     238 RGFGDGYNGYGGGPGGGNFGGSPGYGGGRGGYGGGGPGYGNQGGGYGGGYDNYGGGNYGS

XP_025002966.1  297 GNYNDFGNYNQQPSNYGPMKSGNFGGSRNMGGPYGGGNYGPGGSGGSGGYGGRSRY
XP_021129883.1  293 GNYNDFGNYNQQPSNYGPMKSGNFGGSRNMGGPYGGGNYGPGGSGGSGGYGGRSRY
XP_013027335.1  293 GNYNDFGNYNQQPSNYGPMKSGNFGGSRNMGGPYGGGNYGPGGSGGSGGYGGRSRY
NP_112533.1     298 GNYNDFGNYNQQPSNYGPMKSGNFGGSRNMGGPYGGGNYGPGGSGGSGGYGGRSRY
```

Figure S19: Schematic alignment of hnRNPA2B1 protein sequences of chicken, duck, goose and human. Depicted are RNA recognition motif domains 1 and 2 in black, and prion-line domain in grey underlining respectively. Note: Amino acids critical for hnRNPA2B1 dimerization (P93, K94, R95, V184, R185, K186; human numbering) and methylation (R238) are conserved across human and avian
